# Active Templating by Mechanochemistry

**DOI:** 10.1021/acs.orglett.6c02540

**Published:** 2026-07-15

**Authors:** Oliver N. Evans, Ana M. Belenguer, Christopher A. Hunter

**Affiliations:** Yusuf Hamied Department of Chemistry, 2152 University of Cambridge, Lensfield Road, Cambridge CB2 1EW, United Kingdom

## Abstract

In this paper, we demonstrate that the active-template synthesis of mechanically interlocked molecules can be achieved under mechanochemical conditions. The active-template copper-catalyzed azide-alkyne cycloaddition (CuAAC) synthesis of a [2]-rotaxane afforded yields of up to 95% and reached completion more rapidly than analogous solution-phase reactions. These results show the compatibility of active templating with mechanochemical synthesis.

## Graphical abstract

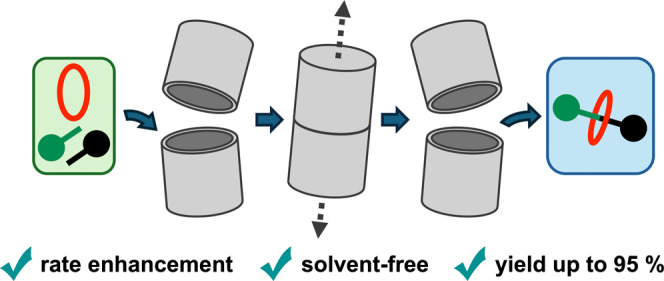

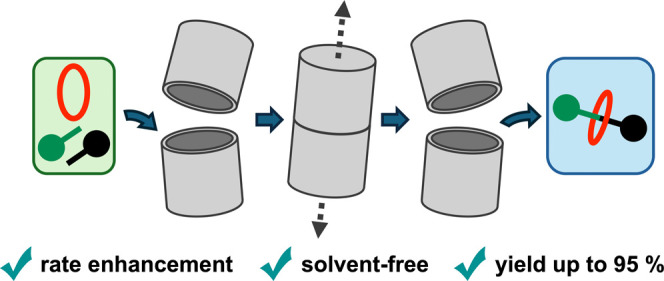

Active templating (AT) is a useful tool for the synthesis of mechanically interlocked molecules.^1–3^ In the first example reported by Leigh,^4^ a pyridine-functionalized macrocycle was used to bind a Cu­(I) ion, which catalyzed the reaction between an alkyne and an azide through the macrocyclic cavity, resulting in a [2]-rotaxane. The active templating approach has been expanded to include multiple reactions and has been used to synthesize a diverse range of complex mechanically interlocked structures, such as rotaxanes,^5–12^ catenanes,^13–16^ and knots.^17^ The molecules synthesized by AT have had a wide variety of applications, such as molecular machines,^18–25^ organocatalysts,^26–28^ and molecular sensors.^29–35^

However, there are limitations:

1. AT reactions often require long reaction times.
2. Metal-mediated AT reactions require noncoordinating solvents to ensure that the metal catalyst is bound within the macrocyclic cavity. These nonpolar solvents can lead to problems with the solubility of the components. Furthermore, the chlorinated solvents that are commonly used for AT are both toxic and environmentally damaging, which limits the potential for industrial applications.
3. Degassed solvents and inert atmospheres are often required to prevent the oxidation of catalytically active metal species.

Mechanochemistry has emerged as a powerful platform for sustainable synthetic chemistry.^36–38^ The application of mechanochemistry to metal-catalyzed reactions, such as copper-catalyzed azide-alkyne cycloaddition (CuAAC), is well reported in the literature.^39^ While there are a few examples in the literature,^40–44^ the application of mechanochemistry to the synthesis of mechanically interlocked molecules remains underexplored. Here, we demonstrate that mechanochemistry can be used as a solvent-free method for the synthesis of [2]-rotaxanes by active templating.^1,7,8^

The milling of Cu­(I), **1**, **2**, and **3** (entry 1) resulted in the formation of **4**, **5**, and **6** (Scheme 1), but a significant proportion of the starting materials **1**, **2**, and **3** remained after 4 h. The products **4**, **5**, and **6** were identified by their mass spectra (Figure 1), and the structure of **5** was further confirmed by NMR (see Supporting Information). No change was observed on continued milling of this reaction mixture. Additional Cu­(I) was added to the jar after 6 h of milling, and the resulting mixture was then milled for a further 2 h; however, no further change was observed in the reaction mixture. When the reaction mixture was milled in the presence of a reducing agent (entry 2; Figure 2), near-complete conversion of the starting materials to products **4**, **5**, and **6** was observed.

**Figure 1:**
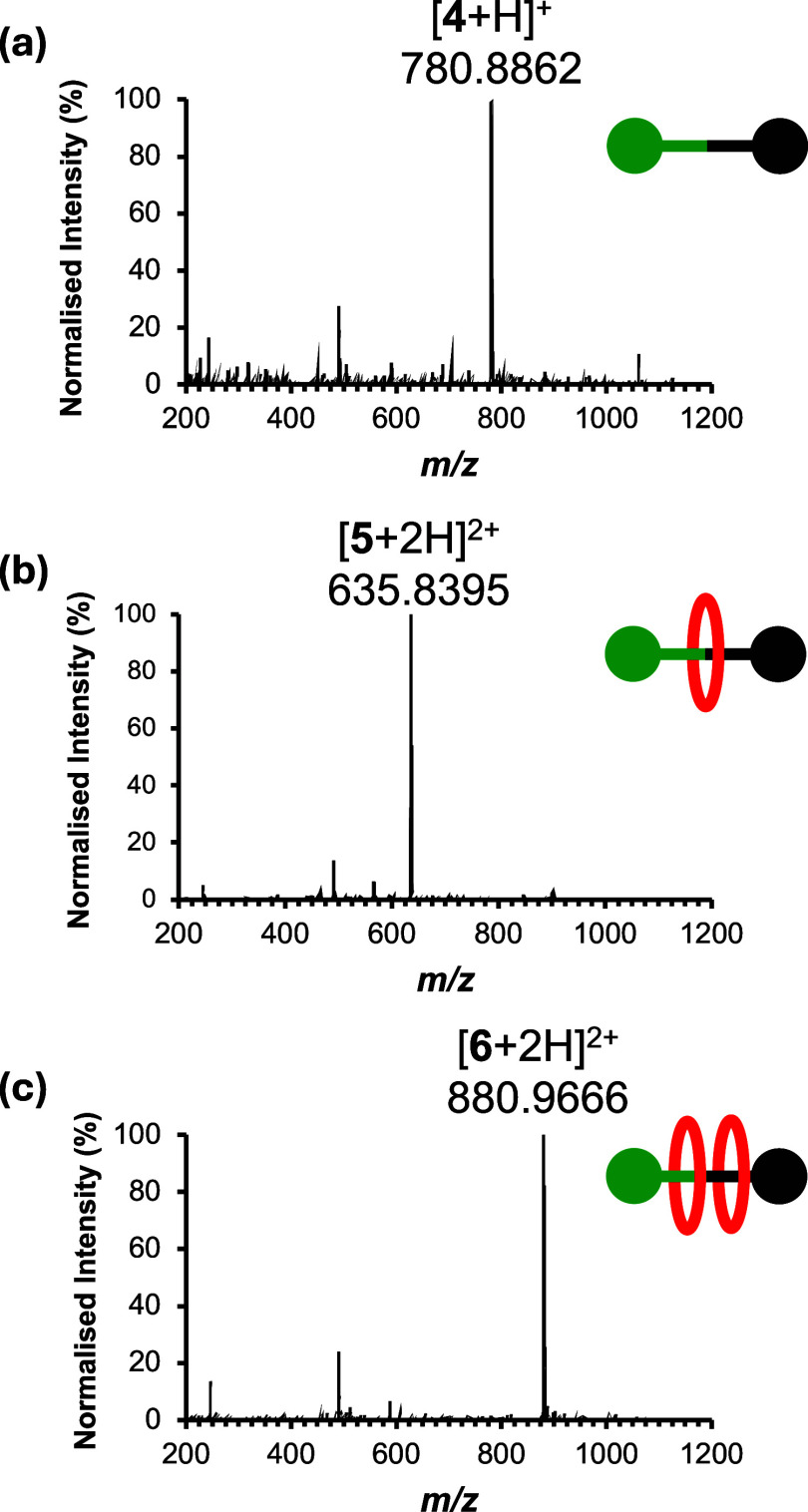
ESI­(+) mass spectra of (a) unthreaded axle **4**. Calculated mass (ESI^+^): 780.3590 [**4** + H]^+^; (b) [2]-rotaxane **5**. Calculated mass (ESI^+^): 634.8223 [**5** + 2H]^2+^; (c) [3]-rotaxane **6**. Calculated mass (ESI^+^): 878.9629 [**6** + 2H]^2+^.

**Figure 2:**
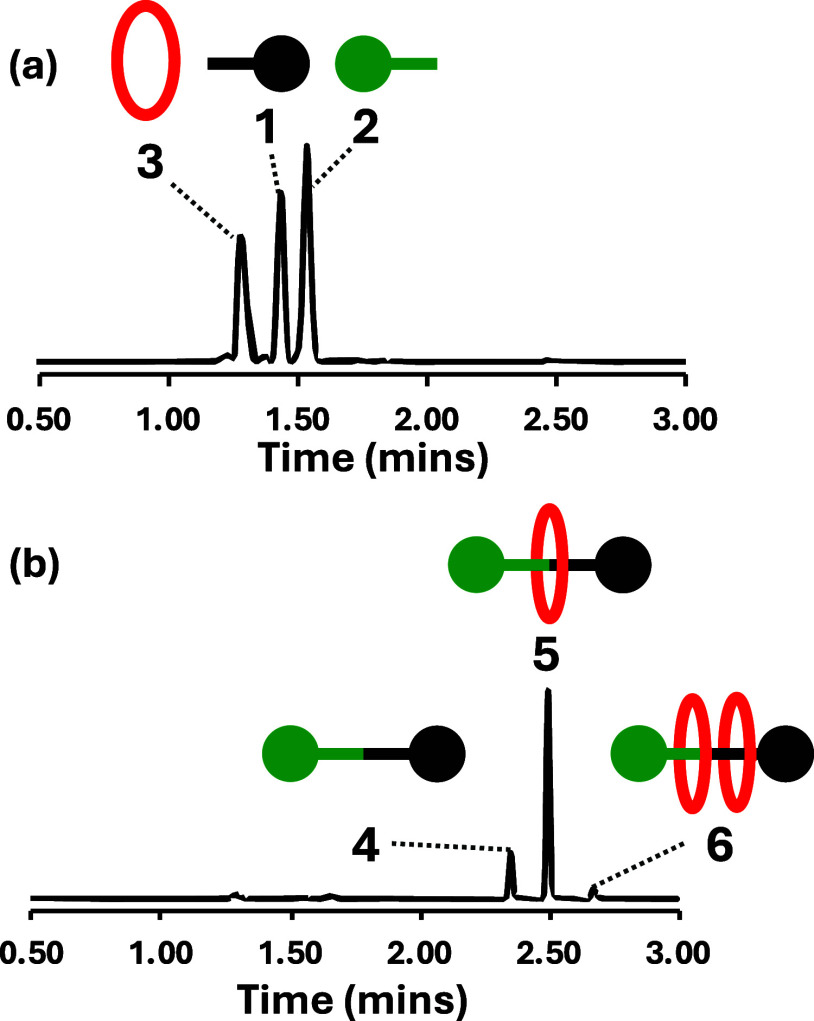
UPLC traces of the crude reaction mixture (entry 2) of azide **1** (1 equiv), alkyne **2** (1 equiv), macrocycle **3** (1 equiv), [Cu­(I)­(MeCN)_4_]­PF_6_ (1 equiv), and sodium ascorbate (5 equiv). (a) Before milling; (b) after milling at 30 Hz for 4 h. UPLC conditions: C4 column at 40 °C using a gradient of 45–65% of MeCN/formic acid (0.1%) in water/formic acid (0.1%) over 1.5 min, then 65–100% MeCN/formic acid (0.1%) over 0.1 min, and then 100% MeCN/formic acid (0.1%) for 1.5 min. The UV–vis absorbance at 254 nm is plotted.

**Scheme 1:**
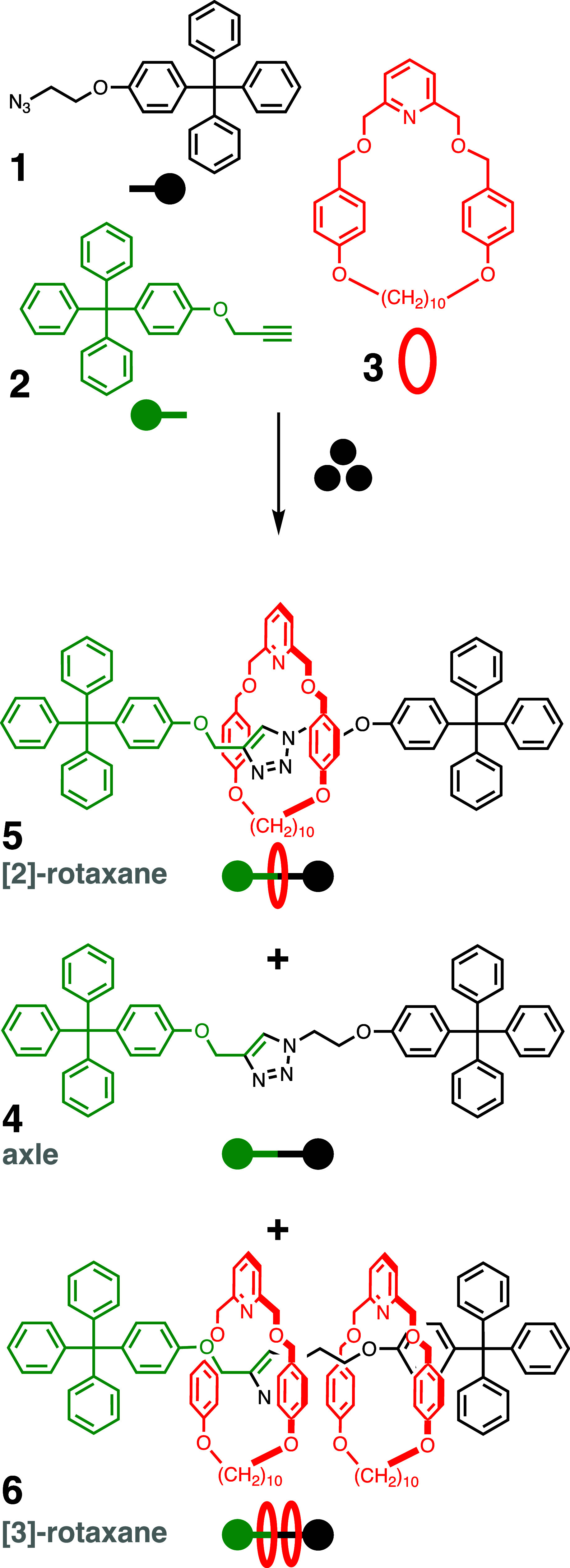
Reaction Scheme for the AT-CuAAC Reaction Investigated in This Study

These results suggest that the oxidation of Cu­(I) to catalytically inactive Cu­(II) led to incomplete conversion of the starting materials (entry 1). Since Cu­(II) forms stronger coordinate bonds with the ligands, the addition of further Cu­(I) was unable to displace the catalytically inactive species, resulting in no further reaction.

The salt used as a source of Cu­(I) was found to have an effect on the product distribution. The use of Cu­(I)I in place of [Cu­(I)­(MeCN)_4_]­PF_6_ (entry 3) resulted in a poorer overall triazole yield. However, the reaction resulted in [2]-rotaxane **5** and [3]-rotaxane **6** as major products and significantly less of axle **4**. It was found that changing the ratio of macrocycle **3** relative to the alkyne and azide affected the product distribution (entry 4). Increasing the ratio of macrocycle **3** promoted active templating, increasing the proportion of both rotaxanes **5** and **6**, while decreasing the proportion of axle **4**. Liquid-assisted grinding (LAG)^37^ with MeCN (entry 5) resulted in excellent conversion to the triazole, giving [2]-rotaxane **5** as the major product. Surprisingly, no [3]-rotaxane **6** was observed.

The CuAAC reaction has been shown to occur in the absence of copper salts by using a milling jar and ball made from copper metal.^45^ Collisions between the ball and the wall of the jar generate copper nanoparticles, which, in turn, act as catalysts for the CuAAC reaction. We hoped to reproduce this result for the AT-CuAAC reaction (entry 6); however, after 5 h of milling, only starting materials **1**, **2**, and **3** were observed and the products **4**, **5**, and **6** were not detected.

The reactions in Table 1 were carried out at an analytical scale (*loading* ∼ 40 mg). The optimized reaction (entry 2) was then scaled up, and reproducible results were achieved at a preparatory scale (*loading* ∼ 0.3 g). The products were isolated, and their identity was confirmed by ^1^H and ^13^C NMR (see Supporting Information). These results demonstrate that the reaction can be carried out at a preparatory scale, offering a green and efficient route to the laboratory-scale synthesis of mechanically interlocked molecules.

**Table 1:** Variations in the Reactant Stoichiometry and Experimental Conditions in the Synthesis of [2]-Rotaxane **5** ^a^

Entry	**3** [eq]	*T* [h]	Conversion **1** → **4** + **5** + **6** [%]	Conversion **3** → **5** [%]	Product selectivity **4**:**5**:**6**
1^a^	1	4	71	26	1.0:4.0:0.0
2^a^ ^,^ ^b^	1	4	93	83	1.0:3.0:0.2
3^b^ ^,^ ^c^	1	4	31	19	1.0:4.6:2.7
4^a^ ^,^ ^b^	5	4	84	–^e^	1.0:12.0:1.0
5^a^ ^,^ ^b^ ^,^ ^d^	1	5	91	95	1.0:5.4:0.0
6^f^	1	5	0	0	

a In all reactions, azide **1** (1 equiv) and alkyne **2** (1 equiv) were present. A general experimental procedure is provided in the Supporting Information.

b With [Cu­(I)­(MeCN)_4_]­PF_6_ (1 equiv) as the copper salt.

c With sodium ascorbate (5 equiv).

d With Cu­(I)I (1 equiv) as the copper salt.

e Liquid-assisted grinding (LAG)^37^ with MeCN (η = 0.1 μL/mg).

f Yield has been excluded because **3** is not a limiting reagent.

g The reaction was carried out using a copper ball and copper milling jar in the absence of copper salt.

A kinetic study of the reaction was conducted, and the results are listed in Figure 3. A mixture of [Cu­(I)­(MeCN)_4_]­PF_6_, **1**, **2**, and **3** was reacted in a vibrational ball-mill grinder. At regular time intervals, the milling was stopped, and a small portion of the reaction mixture was analyzed by UPLC (see Supporting Information). The kinetic profile for the formation of unthreaded axle **4** showed three distinct phases: an induction period in which the rate is limited by particle mixing and molecular diffusion; a rapid reaction regime; and a saturation regime.^46^ In contrast, **6** was observed after 10 min of milling, and the percentage of **6** did not change significantly with time. Similarly, **5** was observed after 10 min, but the percentage of **5** increased over the next 90 min after which point no change was observed. The induction period observed for **4** was mirrored in the powder morphology (Figure 3b). The starting materials were crystalline solids, but after 10 min of milling, a waxy morphology was observed. After 50 min of milling, a transition to a crystalline morphology was observed, coinciding with the start of the rapid reaction regime.

**Figure 3:**
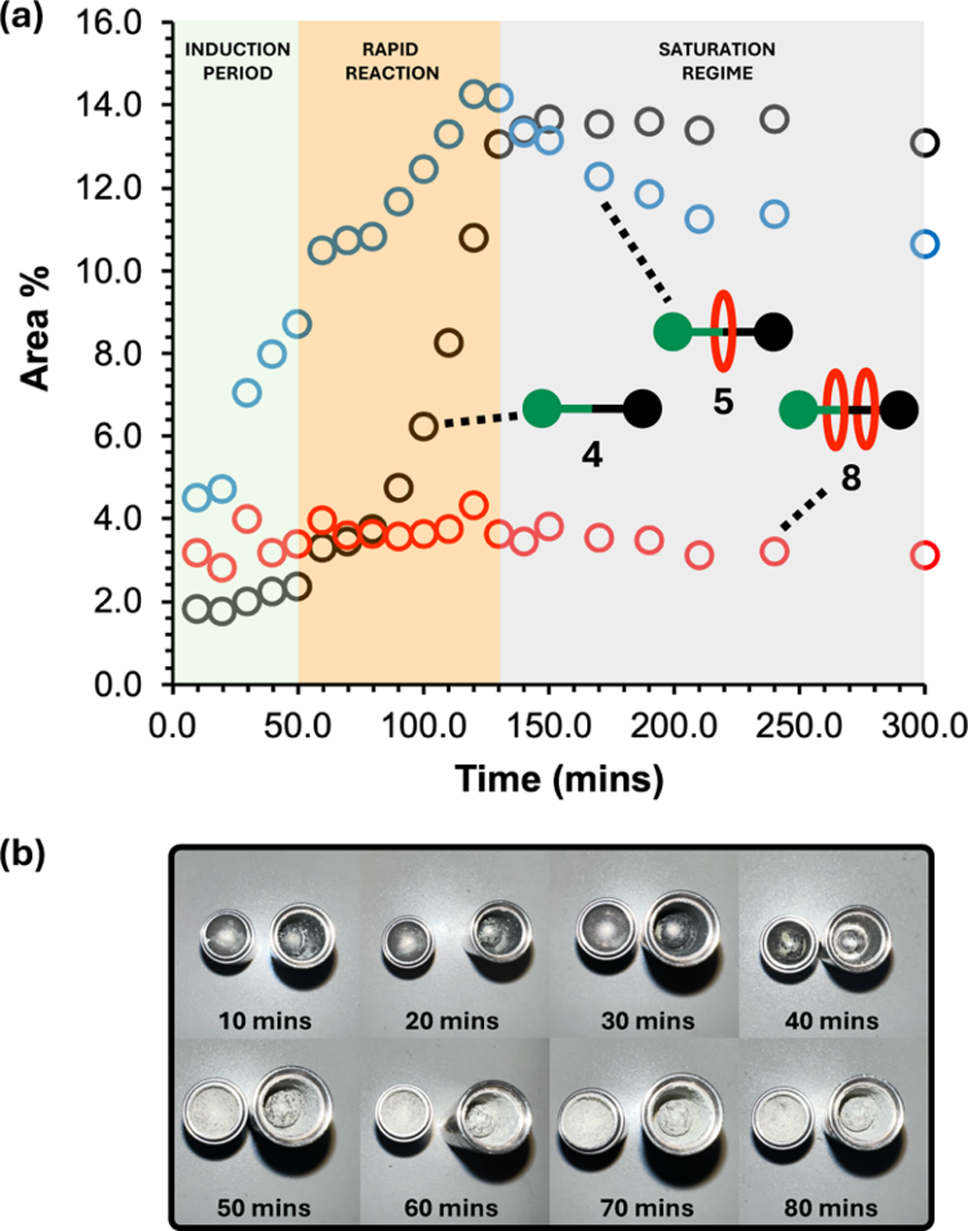
(a) UPLC peak integrals of products axle **4**, [2]-rotaxane **5**, and [3]-rotaxane **6** in the crude reaction mixture (Table 1; entry 1) shown as a percentage of total peak area. Peaks were assigned to axle **4** (black), [2]-rotaxane **5** (blue), and [3]-rotaxane **6** (red). The kinetic profile for the formation of axle **4** occurred in three phases: 1. an induction period (green background); 2. a rapid reaction (orange background), and 3. a saturation regime (gray background).^46^ (b) Photographs taken of the milling jar during the kinetic study. A distinct transition in powder morphology was observed between 40 and 50 min.

This work shows that mechanochemistry is a useful tool for the high-yielding synthesis of mechanically interlocked molecules using active templating (AT). The high concentrations used in the mechanochemical approach result in fast reaction rates, and the range of potential substrates that can be used is not limited by solubility in nonpolar solvents. Furthermore, this approach offers a green alternative to traditional methods that rely on toxic and environmentally damaging solvents.

## Supplementary Material



## Data Availability

The data underlying this study are available in the published article and its Supporting Information.

## Notes

Experimental procedures, NMR spectra, and characterization data of compounds (PDF)
