# Supplementary material for: Active Templating by Mechanochemistry
Source: Org Lett. 2026 Jul 15;28(30):9702–6. doi: 10.1021/acs.orglett.6c02540 (PMC13435305; doi:10.1021/acs.orglett.6c02540)
Supplement: Supplementary file 1 [file ol6c02540_si_001.pdf]

Active Templating by Mechanochemistry

Oliver N. Evans*, Ana M. Belenguer and Christopher A. Hunter

Yusuf Hamied Department of Chemistry, University of Cambridge, Lensfield Road, Cambridge CB2 1EW, UK.

Email: 02oevans@gmail.com

Supplementary Information

Table of contents

1 General experimental methods....2

1.1 Ball Mill Grinder and Milling Jars.... 3

1.2 Handling Sodium Azide[2].... 4

1.3 Low Molecular Weight Organic Azides[2].... 4

2 Compound synthesis and characterisation....5

2.1 Synthesis of 1, 2 and 3.... 5

2.1.1 Compound 7.... 6

2.1.2 Compound 1.... 10

2.1.3 Compound 2.... 14

2.1.4 Compound 8.... 18

2.1.5 Compound 3.... 22

2.2 AT-CuAAC Product Characterisation....27

2.2.1 Compound 4 (axle).... 27

2.2.2 Compound 5 ([2]-rotaxane).... 30

3 General Procedures for AT-CuAAC by milling....38

3.1 Optimised reaction procedure....38

3.2 Reaction procedure for analytical AT-CuAAC by milling....38

3.3 Reaction procedure for kinetic study of AT-CuAAC by milling....39

4 References.... 41

1 General experimental methods

Commercial reagents were purchased from Sigma-Aldrich, Alfa Aesar, Fisher Scientific, Fluorochem or Acros Organics and were used without further purification unless otherwise specified. All air and moisture-sensitive manipulations were carried out using standard vacuum line and Schlenk techniques. Anhydrous THF and CH_2Cl_2 were obtained from a Grubbs PS-MD-5 solvent purification system. Thin-layer chromatography was carried out using silica gel 60F (Merck) on glass plates and visualised using UV light (254 or 365 nm) and/or developed using potassium permanganate. Flash chromatography was carried out on an automated system (Combi*Flash*® Rf$^+$ or Combi*Flash* Rf$^+$ Lumen) using prepacked cartridges of silica (25 µm or 50 µm PuriFlashâ columns). All NMR spectroscopy was carried out on a Bruker 400 MHz DPX400, 400 MHz AVIII400, 500 MHz DCH cryoprobe, 500 MHz TCI Cryoprobe or 700 MHz TXOCryo spectrometer using the residual solvent as the internal standard. All chemical shifts (δ) are quoted in ppm and coupling constants given in Hz. UPLC analysis of samples was performed using Waters Acquity H-class UPLC coupled with a single quadrupole Waters SQD2. Three different columns were used: Acquity UPLC CSH C18 column (130 Å, 1.7 µm, 2.1 mm x 50 mm), Acquity UPLC BEH C8 column (130 Å, 1.7 µm, 2.1 mm x 50 mm) and Acquity UPLC BEH C4 column (300 Å, 1.7 µm, 2.1 mm x 50 mm). Analytical reversed-phase HPLC was performed on an Agilent HP-1100 Series HPLC system, using a XBridge® BEH C8 (2.5 µm, 4.6 x 75 mm) column. Preparative reversed- phase HPLC was performed on an Agilent HP-1100 Series preparative HPLC system, using an XBridge® BEH C8 (5 µm, 19 x 250 mm) column. HRMS analysis was performed in an Agilent 1100 pump and autosampler with a Waters LCT Premier TOF (Time of Flight) mass spectrometer.

1.1 Ball Mill Grinder and Milling Jars

Analytical scale reaction was performed in a milling jar (440C stainless steel; snap closure with no spacer; 11 x 24 mm; 1.9 mL) with a single milling ball (440C stainless steel; 5 mm; 0.5 g). Preparative scale reaction was performed in a milling jar (440C stainless steel; screw closure with a teflon spacer; 18 x 50.5 mm; 14.5 mL) with a single milling ball (440C stainless steel; 10 mm; 4.0 g). Copper analytical scale reaction was performed in a milling jar (copper; snap closure with no spacer; 11 x 24 mm; 1.9 mL) with a single milling ball (copper; 8 mm; 2.4 g). Milling was performed in a horizontal vibrational ball mill grinder (Retsch, MM400;).

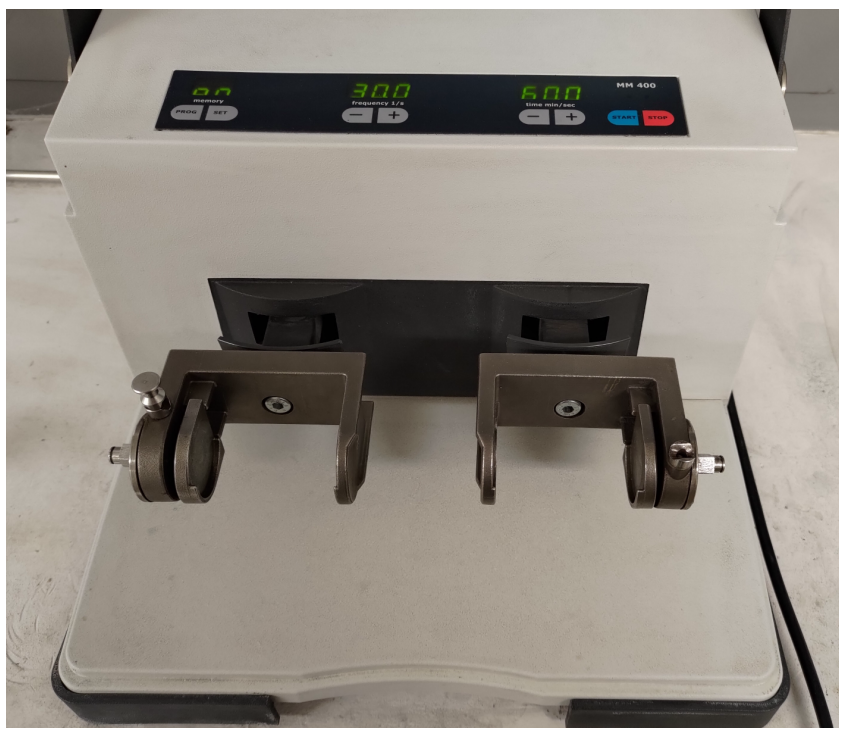

Figure S1 Horizontal vibrational ball mill grinder used in this study (Retsch, MM400).

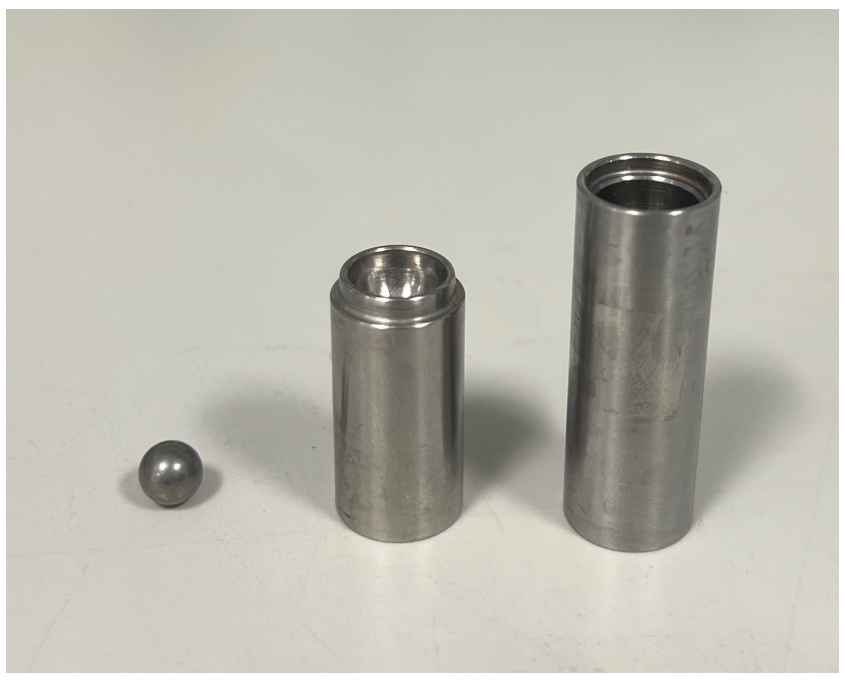

Figure S2 Analytical scale reaction was performed in a milling jar (440C stainless steel; snap closure with no spacer; 11 x 24 mm; 1.9 mL) with a single milling ball (440C stainless steel; 5 mm; 0.5 g).

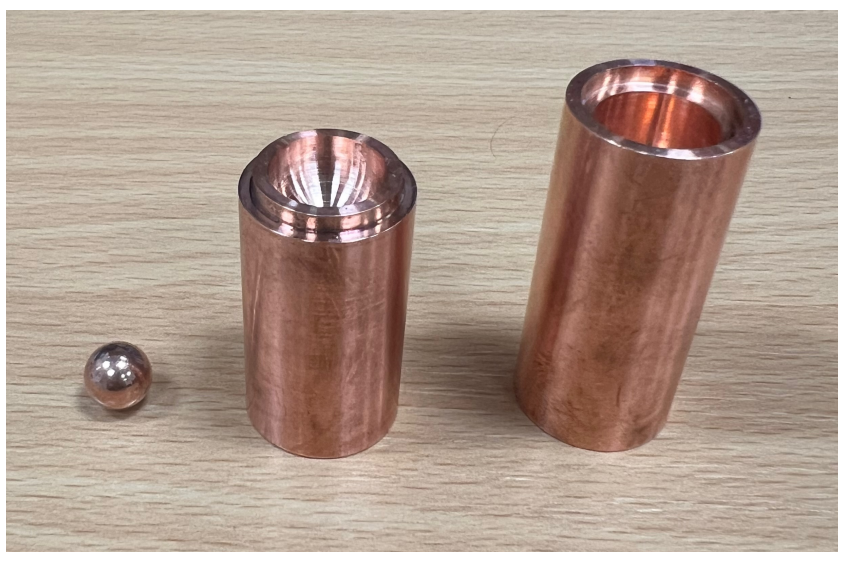

Figure S3 Copper analytical scale reaction was performed in a milling jar (copper; snap closure with no spacer; 11 x 24 mm; 1.9 mL) with a single milling ball (copper; 8 mm; 2.4 g).

1.2 Handling Sodium Azide

Sodium azide is highly toxic (LC_{50} Inhalation = 37 mg/m3 for rats, LD50 Dermal = 20mg/kg for rabbits) and highly soluble in water (>30 g/100 mL at 0 °C). Sodium azide can be absorbed dermally and, therefore must be handled with appropriate personal protection equipment. Sodium azide decomposes above 275 °C, generating highly reactive sodium metal. Sodium azide is not compatible with any acid as it forms highly explosive hydrazoic acid on contact, even in dilute solution.

This warning has been adapted from the literature.[1]

1.3 Low Molecular Weight Organic Azides

Low molecular weight organic azides are potentially explosive substances that can decompose with a slight input of external energy (heat, friction, pressure etc). Although we have not experienced any explosions thus far, any organic azides where the weight attributed to the azido group exceeds 25% of the molecular weight should be handled with caution. Use of a blast shield and avoidance of very large-scale reactions when dealing with these substances is highly recommended.

This warning has been adapted from the literature.[1]

2 Compound synthesis and characterisation

2.1 Synthesis of 1, 2 and 3

Compounds **2**, **3**, **7** and **8** have been previously reported in the literature.[1–5]

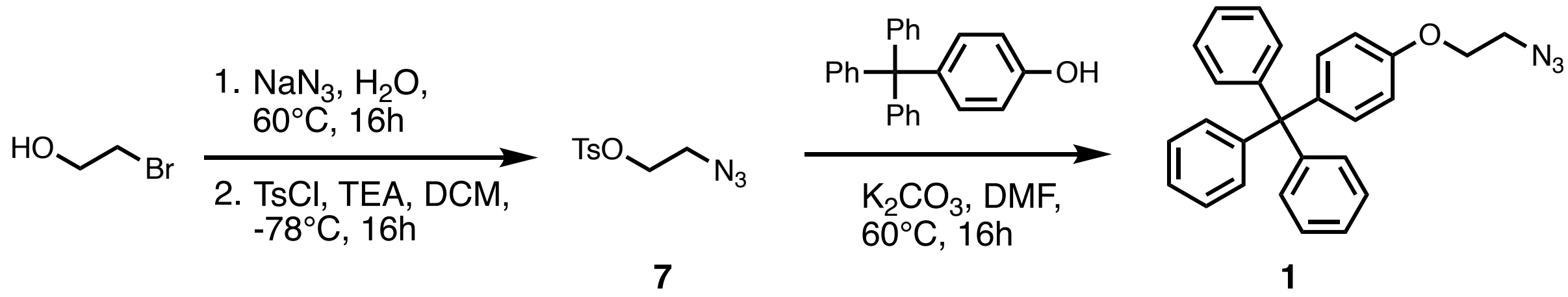

Scheme 1 Synthesis of azide **1**.

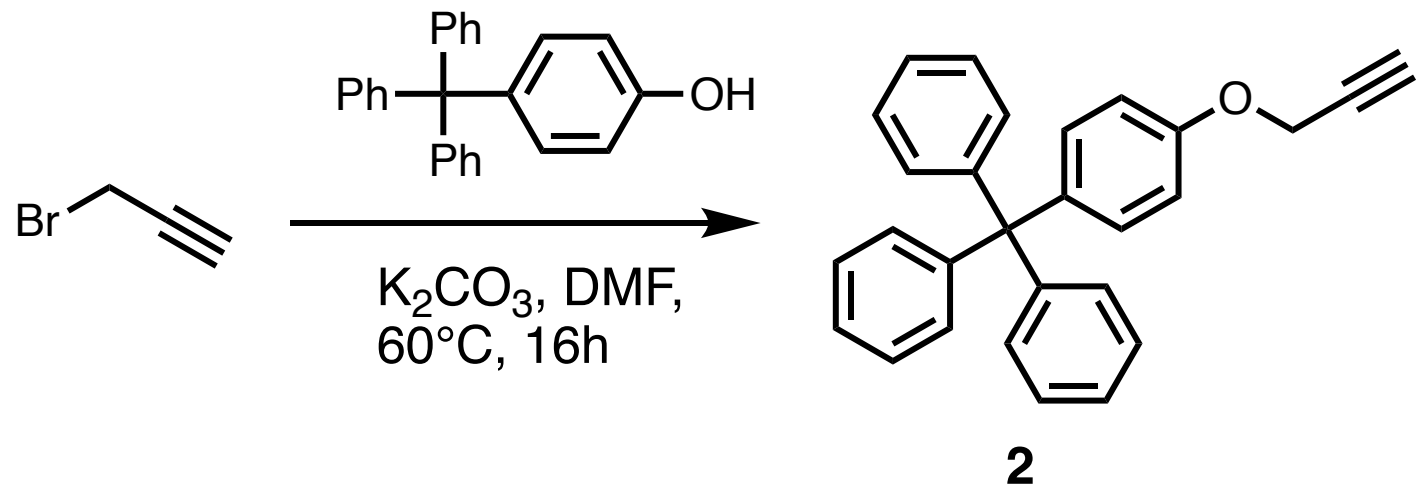

Scheme 2 Synthesis of alkyne **2**.

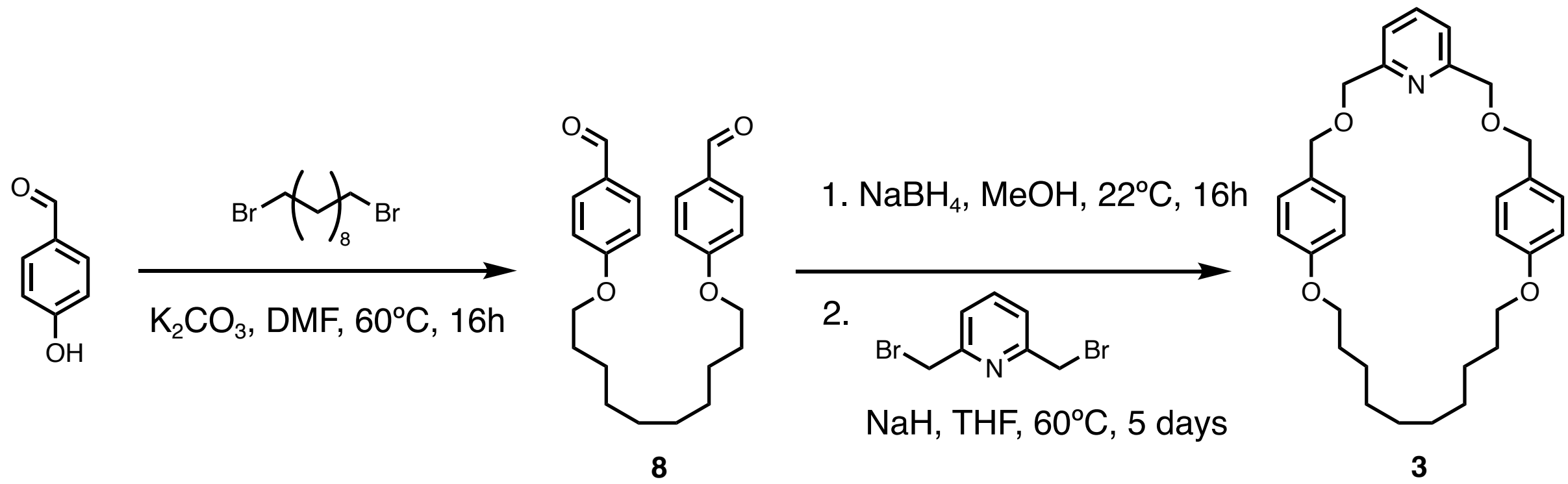

Scheme 3 Synthesis of macrocycle **3**.

2.1.1 Compound 7

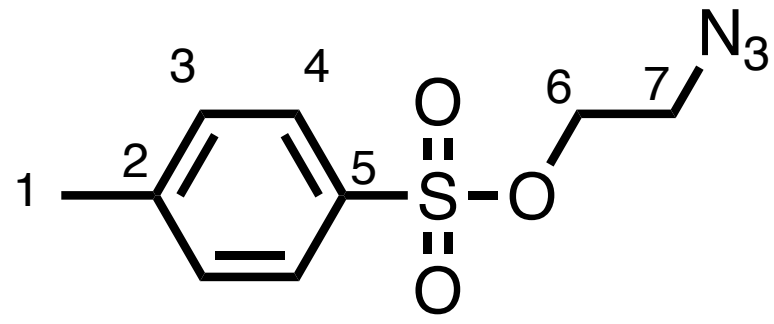

This procedure makes use of ***sodium azide*** *which is both highly toxic and an explosive hazard. In this procedure a* ***low molecular weight organic azide*** *is generated as an intermediate, this compound is potentially an explosive substance. Please read both 1.2 and 1.3 for further details about safe handling.*

Sodium azide (1.32 g, 20.3 mmol, 1 eq) and 2-bromoethan-1-ol (1.69 g, 13.5 mmol, 1 eq) were dissolved in H_2O/THF (1:1, 10 mL). The resulting mixture was stirred for 18 hours at 50 °C, using a heating mantle. The reaction was then cooled to 20 °C and extracted with Et_2O (3 × 20 mL). The combined organic extracts were dried ($MgSO_4$) and the solvent was removed *in vacuo*. The crude mixture was used in the next step without further purification.

The residue and tosyl chloride (3.86 g, 20.2 mmol, 1.5 eq) were dissolved in dry DCM (70 mL) under an N_2 atmosphere, followed by the addition of triethylamine (2.05 g, 20.2 mmol, 1.5 eq). The solution was stirred for 16 hours at 20 °C and quenched with H_2O (50 ml). The organic layer was separated and the aqueous layer was extracted with DCM (2 × 50 mL). The combined organic extracts were dried ($MgSO_4$) and the solvent was removed *in vacuo*. The compound was purified by flash column chromatography (SiO_2, 0-5% EtOAc in PetEther) to afford **7** as a colourless oil (2.2764 g, 9.44 mmol, 70 % over two steps).

Characterisation agreed with the literature data.[1]

^{1}H NMR (400 MHz, $CDCl_3$) δ 7.86 – 7.78 (m, 2H, C4H), 7.41 – 7.33 (m, 2H, C3H), 4.20 – 4.13 (m, 2H, C6H_2), 3.48 (t, *J* = 5.1 Hz, 2H, C7H_2), 2.46 (s, 3H, C1H_3).

^{13}C NMR (101 MHz, $CDCl_3$) δ 145.4 (C5), 132.7 (C2), 130.1 (C3), 128.1 (C4), 68.2 (C6), 49.7 (C7), 21.8 (C1).

FT-IR (ATR): ν_{max}/cm^{-1} 2927, 2108, 1598, 1442, 1359, 1294, 1189, 1173, 1096, 1017, 909, 814, 767, 661, 576, 551, 447.

HRMS (ES$^+$): calculated for $C_9H_{12}N_3O_3S + H^+$ is 242.0599, found 242.0599.

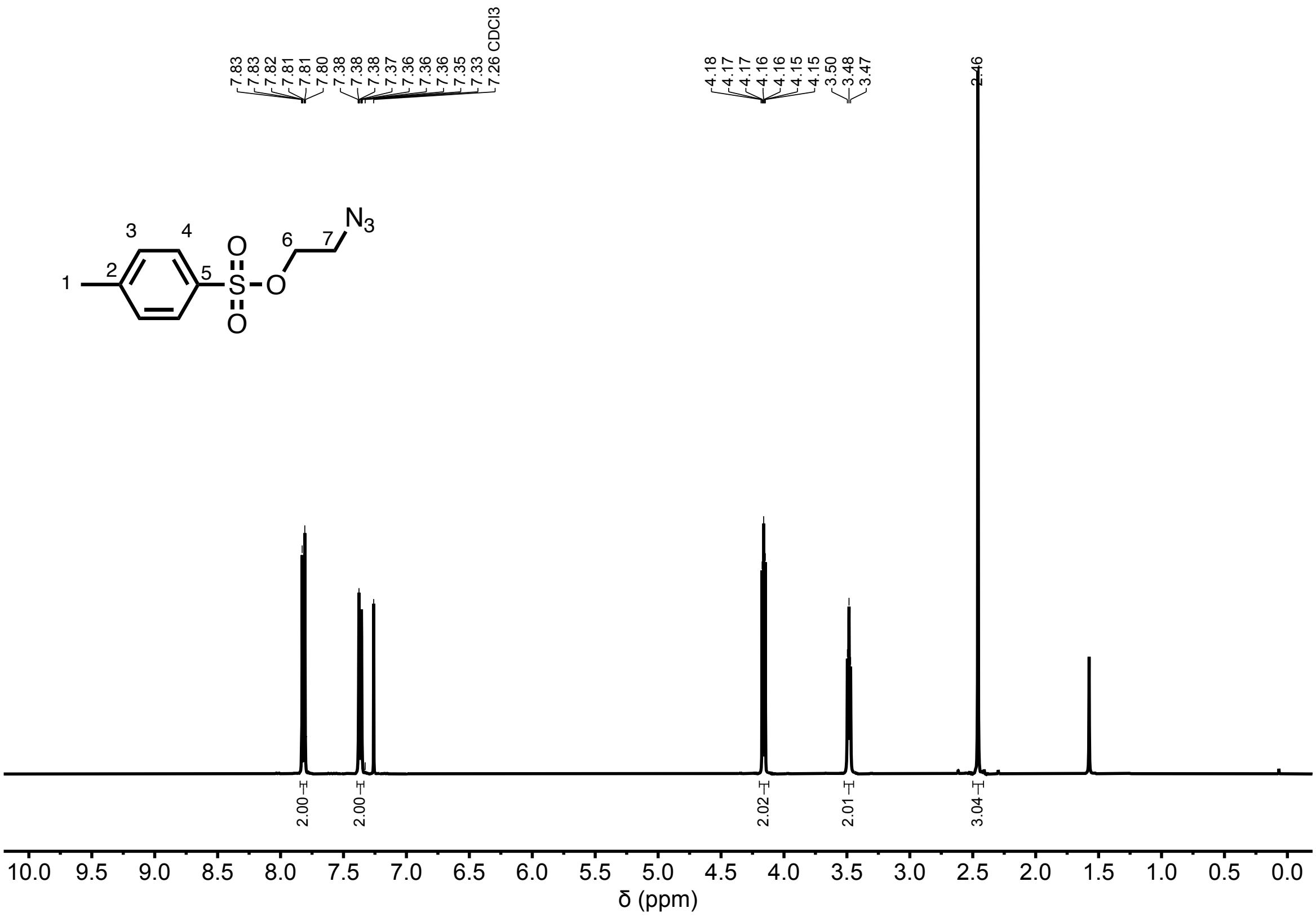

Figure S4 ^{1}H NMR (400 MHz, $CDCl_3$) spectrum of compound **7.**

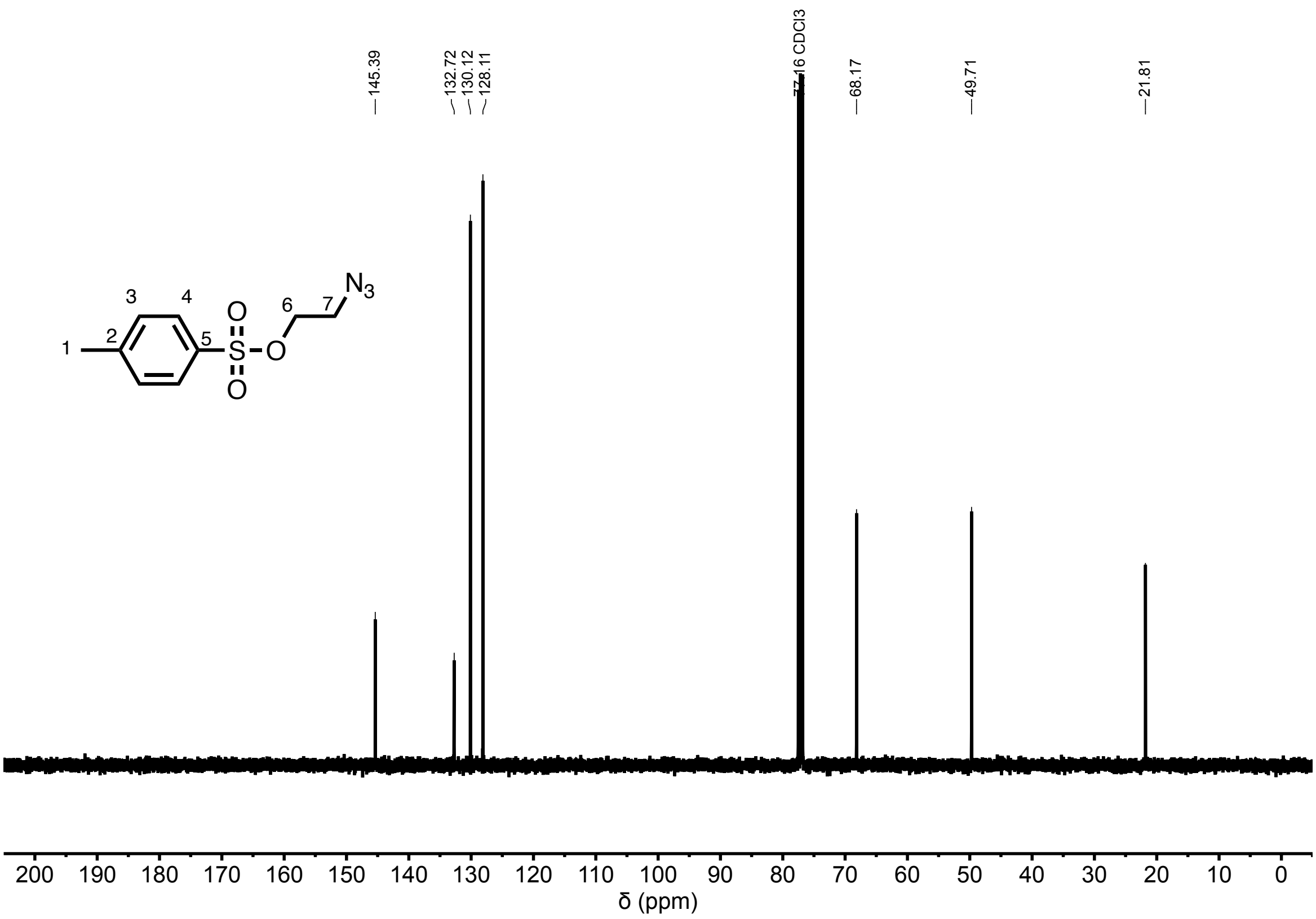

Figure S5 ^{13}C NMR (101 MHz, $CDCl_3$) spectrum of compound **7.**

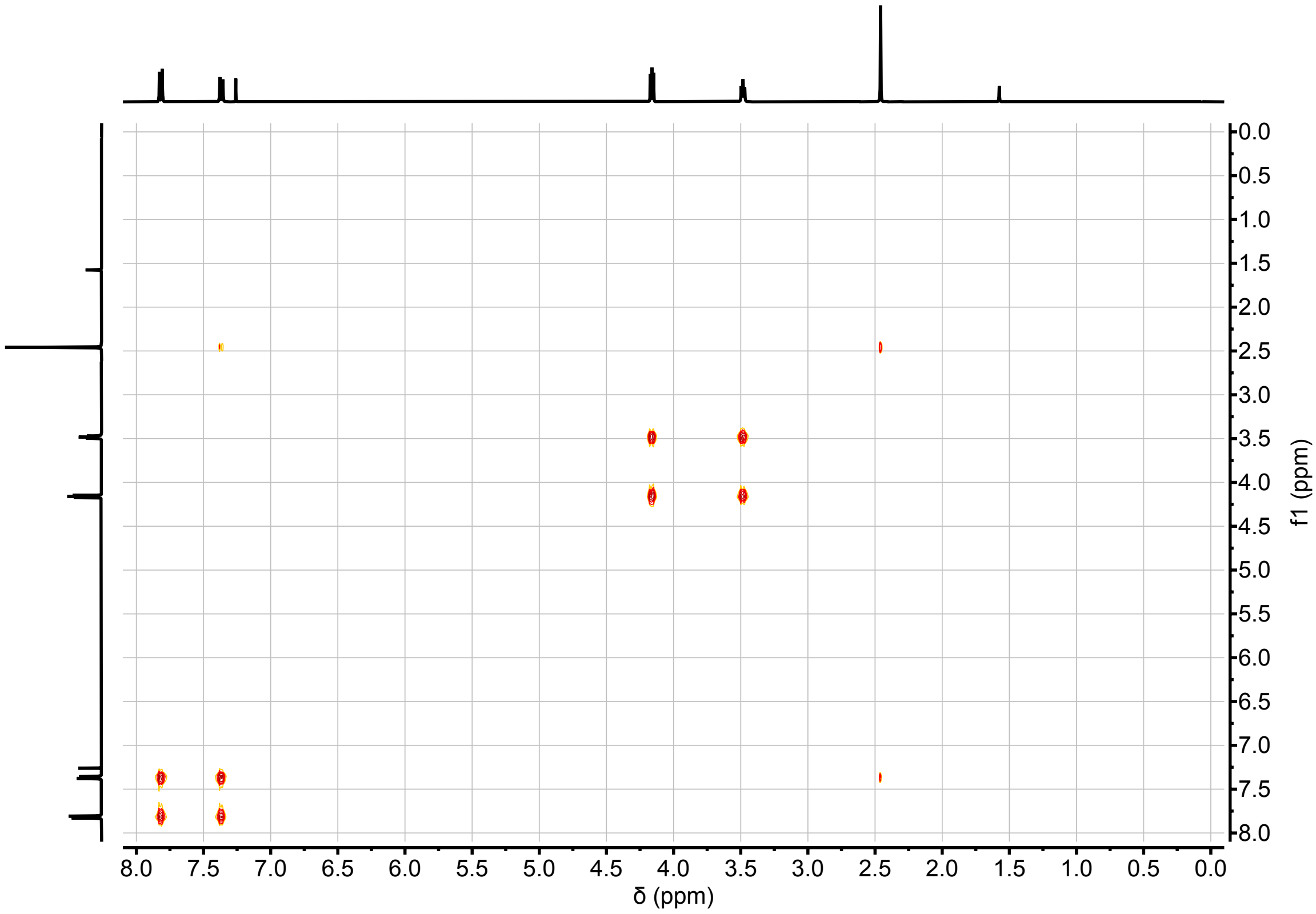

Figure S6 ^{1}H-^{13}C COSY NMR spectrum of compound **7**.

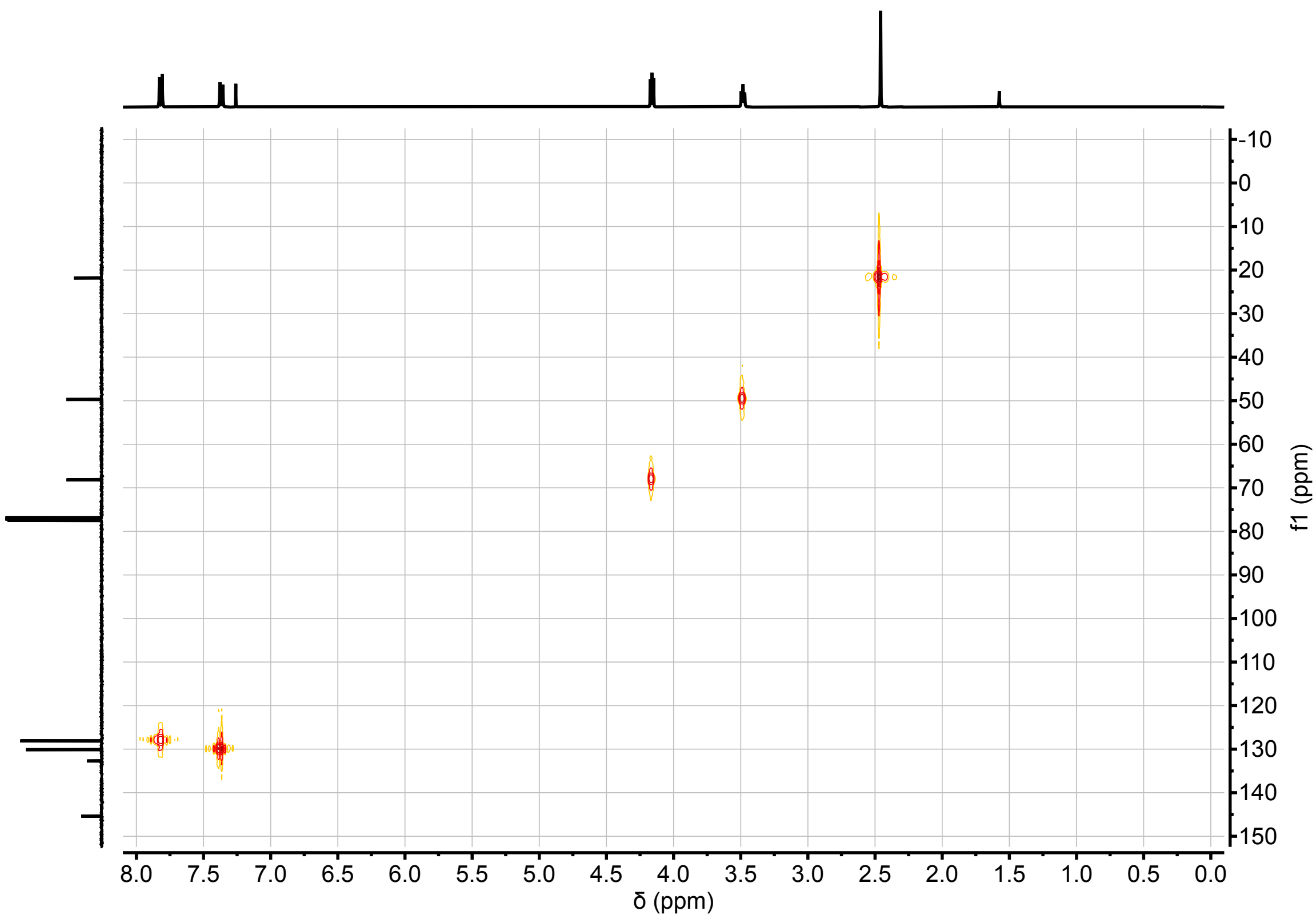

Figure S7 ^{1}H-^{13}C HSQC NMR spectrum of compound **7**.

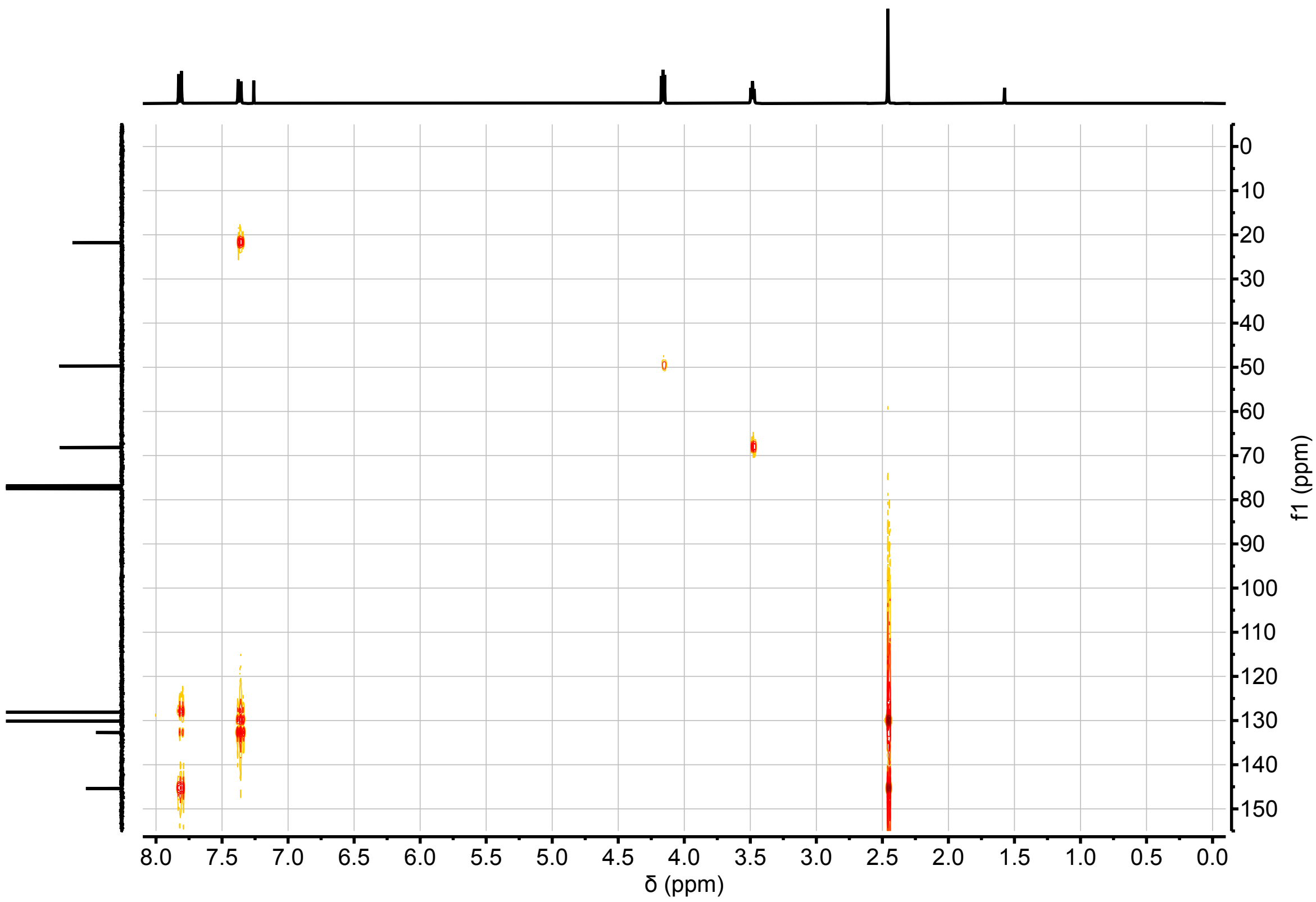

Figure S8 ^{1}H-^{13}C HMBC NMR spectrum of compound **7**.

2.1.2 Compound 1

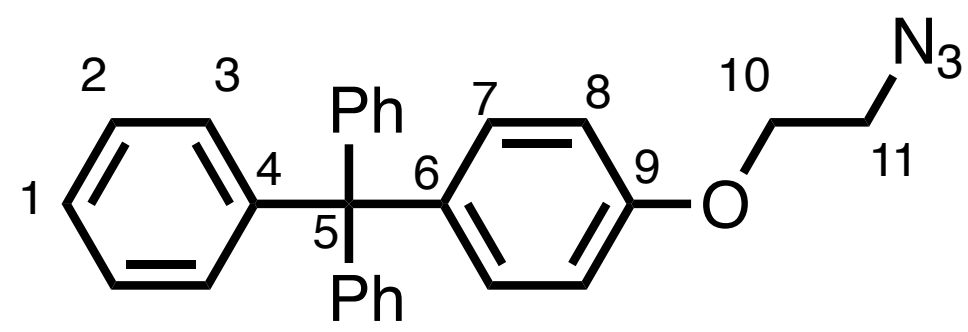

Compound **7** (1.18 g, 4.88 mmol 1 eq), 4-trityl phenol (1.64 g, 4.88 mmol, 1 eq) and K_2CO_3 (1.54 g, 11.2 mmol, 2.3 eq) were dissolved in DMF (50 mL) and stirred for 16 hours at 60°C, using a heating mantle. The solvent was removed *in vacuo* and the residue was redissolved in EtOAc (50 mL). The organic layer was washed with H_2O (50 mL) and 5% $LiCl_{(aq)}$ (50 mL). The organic layer was dried ($MgSO_4$) and the solvent was removed *in vacuo*. The compound was purified by flash chromatography (SiO_2, 0-7% EtOAc in PetEther) to afford the product as a white crystalline solid (1.34 g, 3.32 mmol, 68 %).

^{1}H NMR (400 MHz, $CDCl_3$) δ 7.25 – 7.16 (m, 15H, C1H, C2H and C3H), 7.15 – 7.09 (m, 2H, C8H,), 6.84 – 6.76 (m, 2H, C7H), 4.13 (dd, *J* = 5.4, 4.6 Hz, 2H, C10H_2), 3.58 (t, *J* = 5.0 Hz, 2H, C11H_2).

^{13}C NMR (101 MHz, $CDCl_3$) δ 156.3 (C9), 147.1 (C4), 139.9 (C6), 132.4 (C9), 131.3 (C3), 127.6 (C2), 126.0 (C1), 113.5 (C7), 67.0 (C10), 64.5 (C5), 50.4 (C11).

FT-IR (ATR): v_{max}/cm^{-1} 3083, 3057, 2931, 2870, 2108, 1607, 1508, 1492, 1444, 1288, 1245, 1184, 1063, 924, 828, 747, 702, 630.

HRMS (ES$^+$): calculated for $C_{27}H_{23}N_3O^+$ is 405.1841, found 405.1860.

m.p.: 156.7 - 158.0 °C

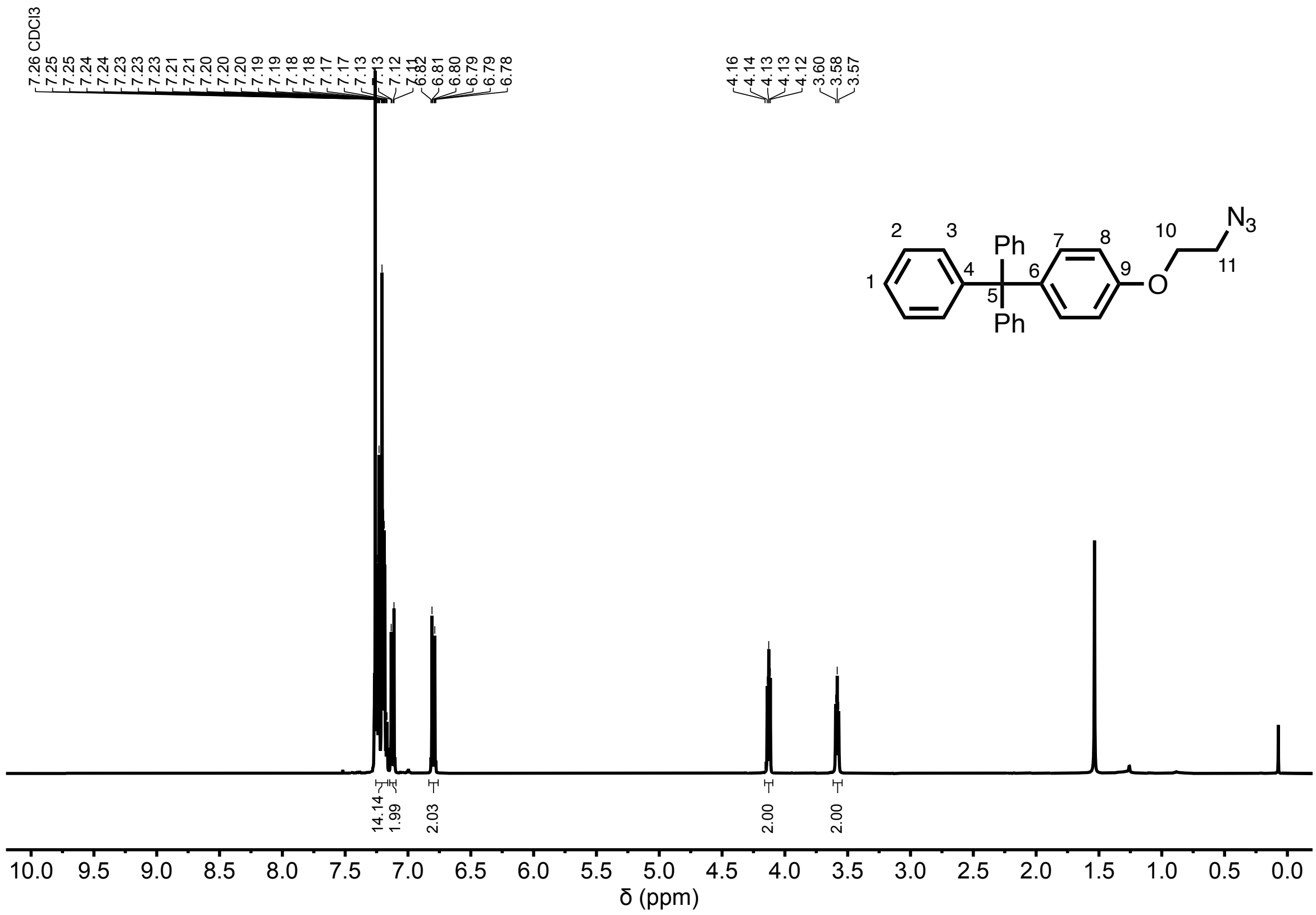

Figure S9 ^{1}H NMR (400 MHz, $CDCl_3$) spectrum of compound **1.**

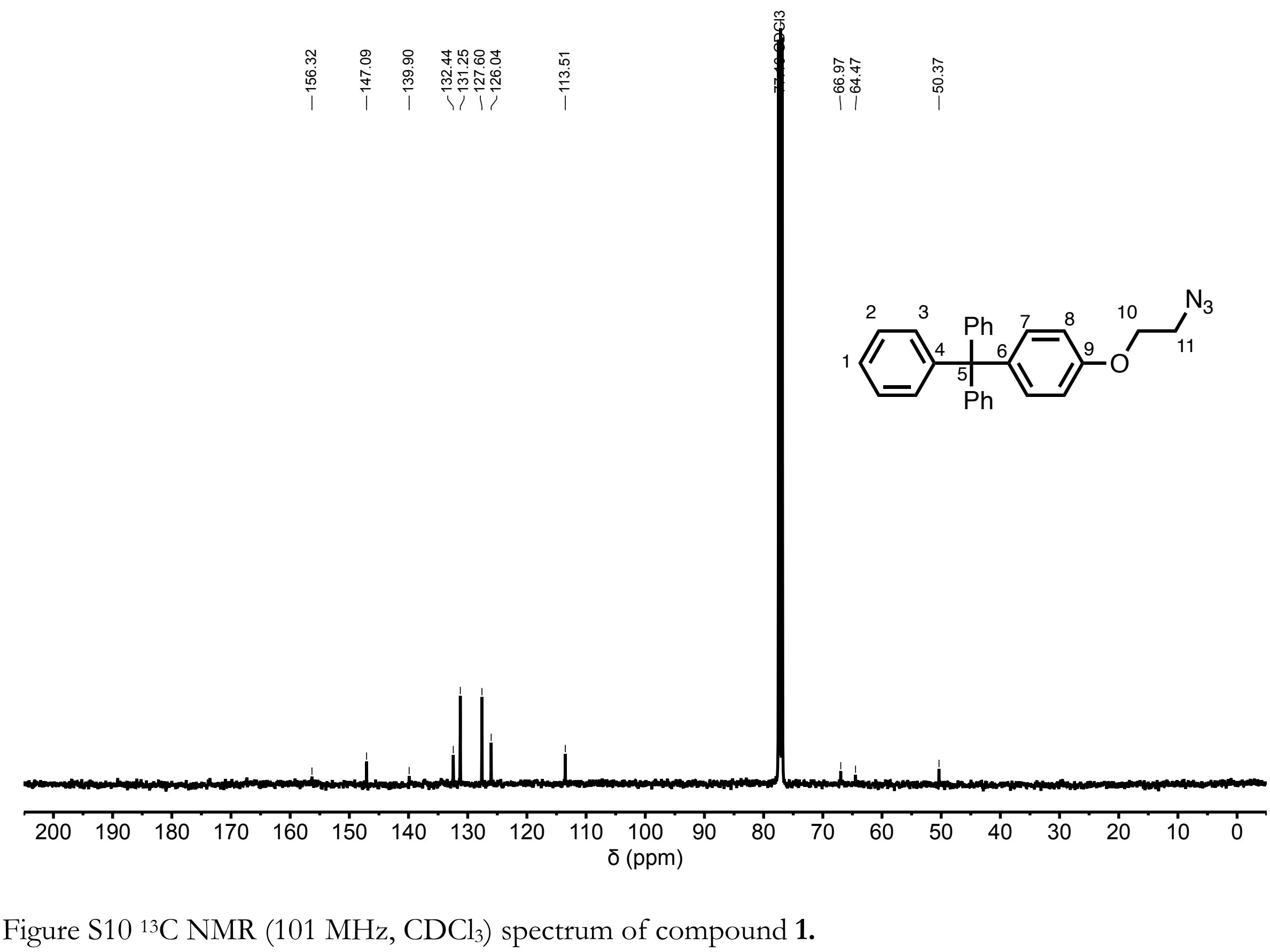

Figure S10 ^{13}C NMR (101 MHz, $CDCl_3$) spectrum of compound **1.**

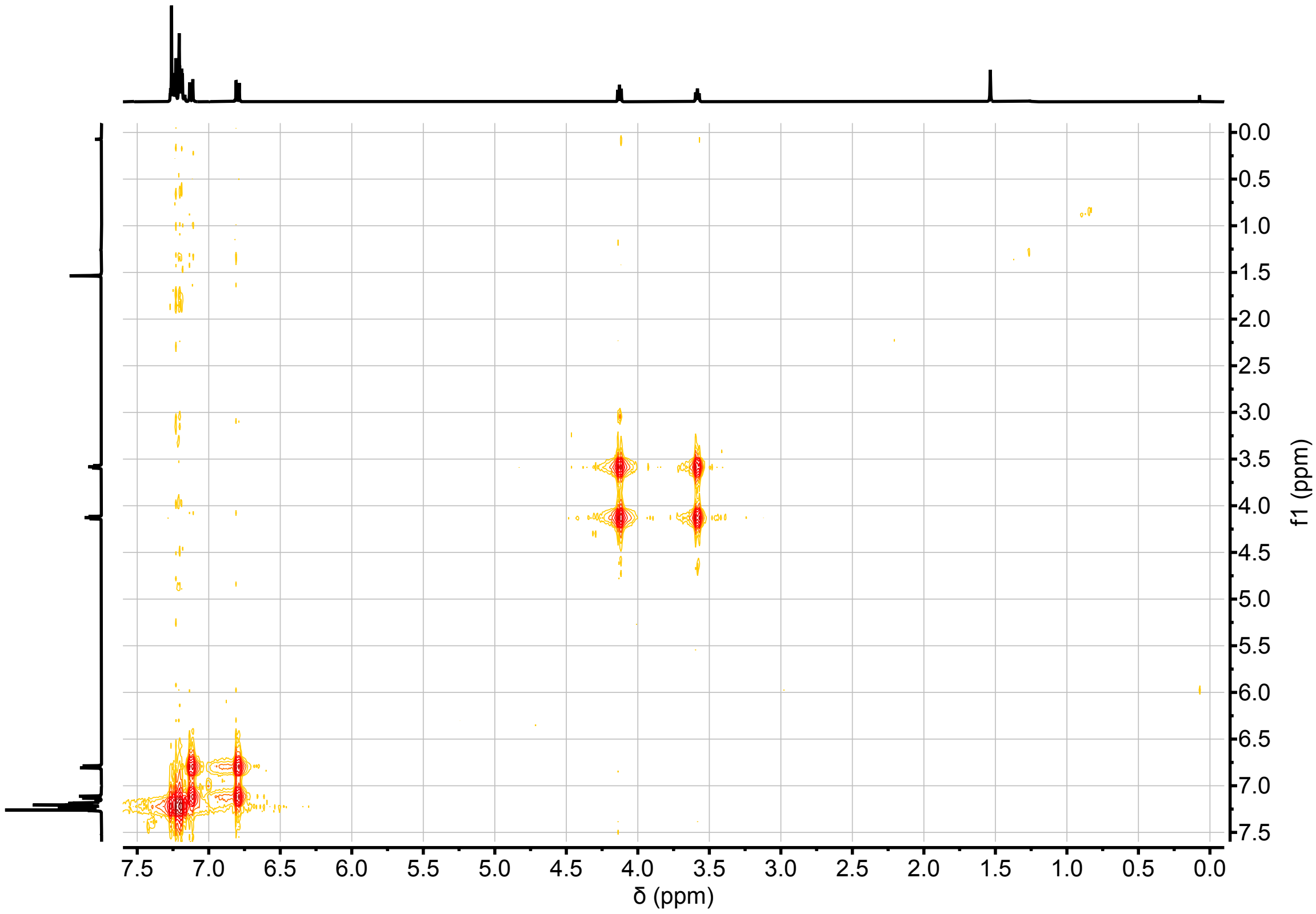

Figure S11 ^{1}H-^{13}C COSY NMR spectrum of compound **1**.

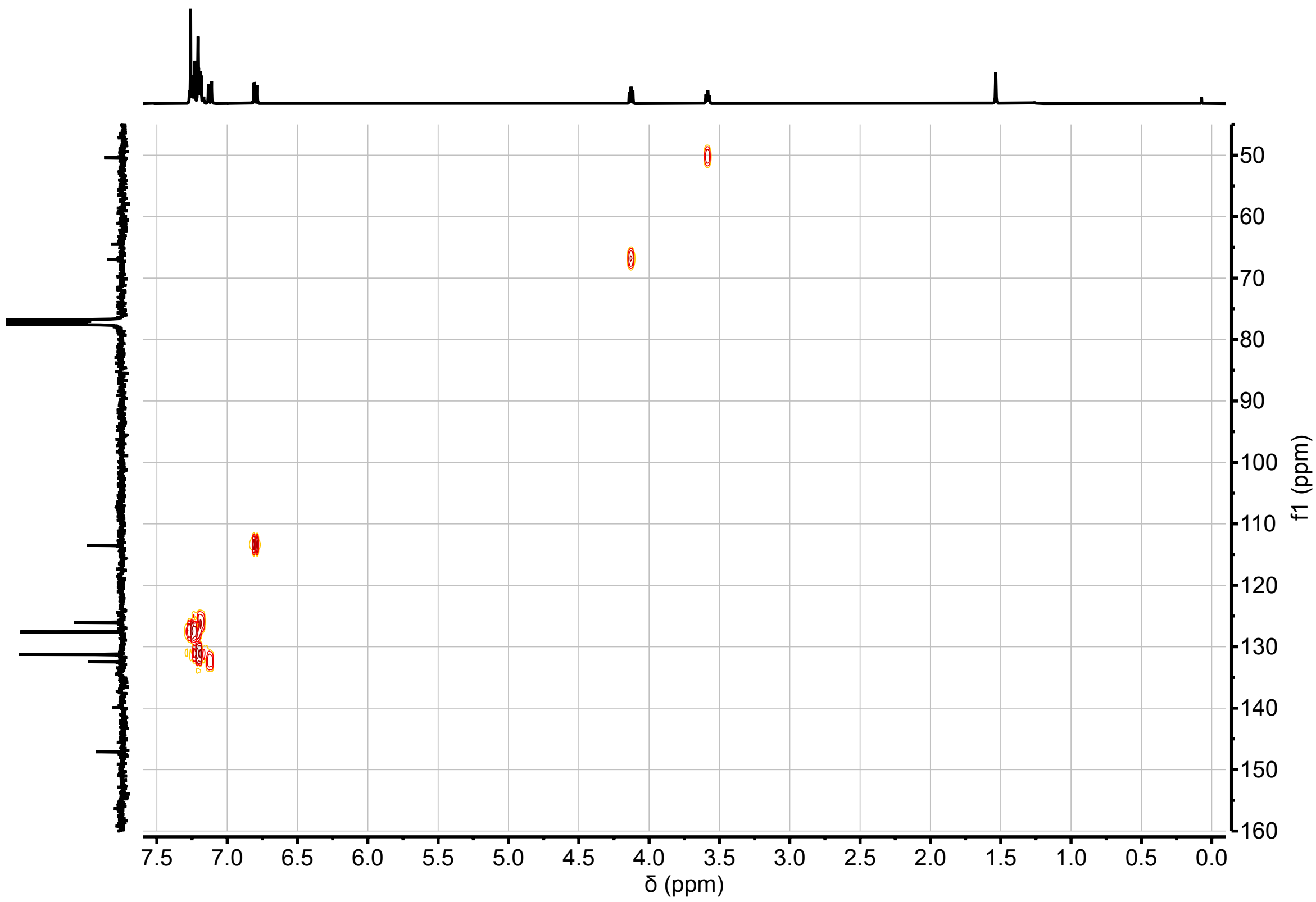

Figure S12 ^{1}H-^{13}C HSQC NMR spectrum of compound **1**.

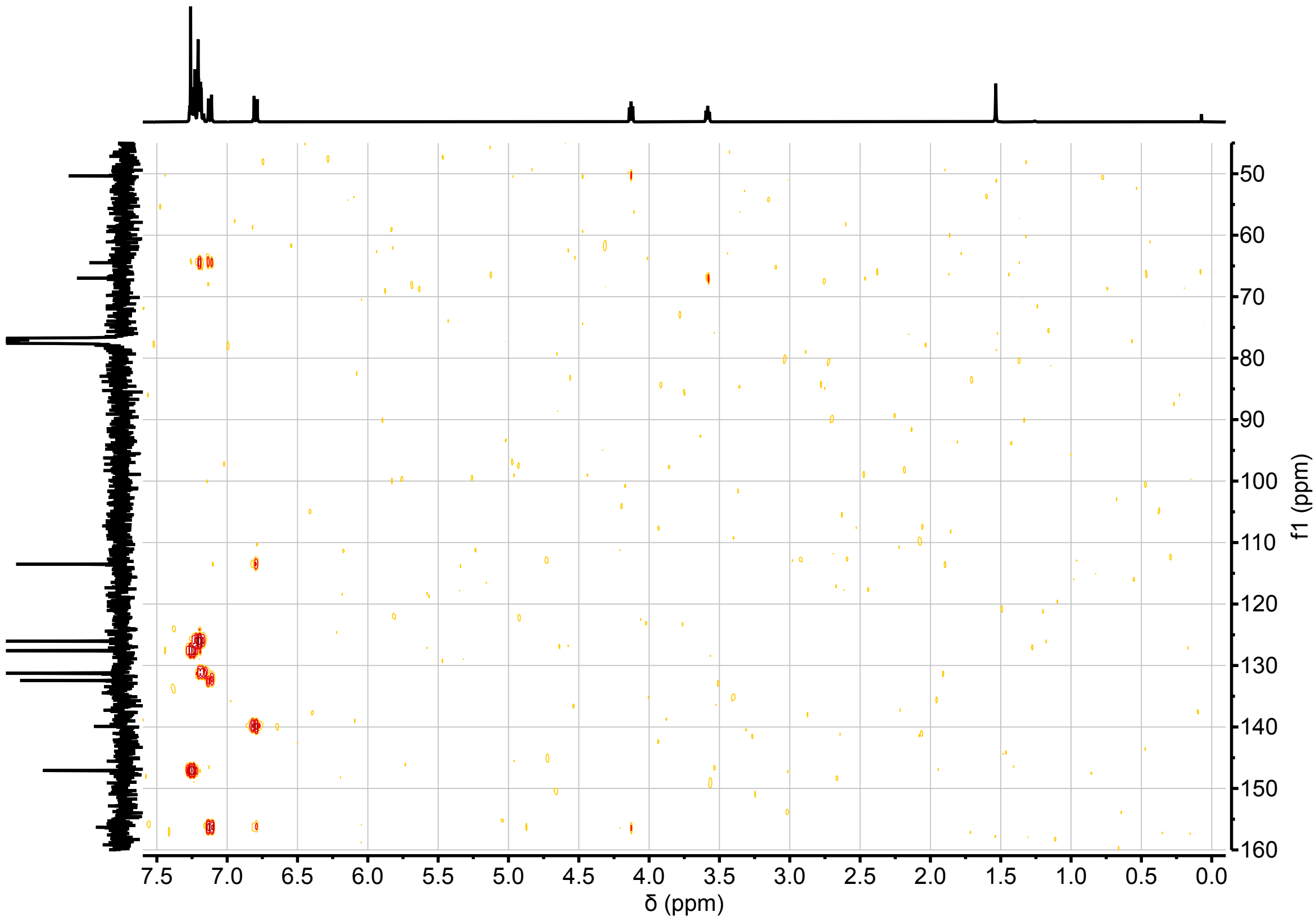

Figure S13 ^{1}H-^{13}C HMBC NMR spectrum of compound **1**.

2.1.3 Compound 2

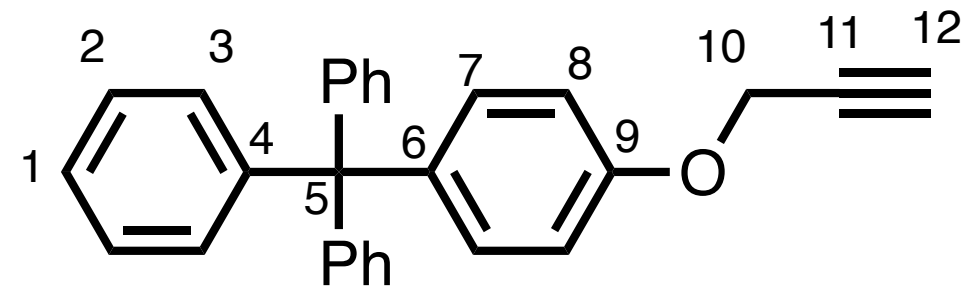

Propargyl bromide (0.625 mL, 0.50 g, 4.2 mmol 1 eq), 4-trityl phenol (1.64 g, 4.88 mmol, 1.1 eq) and K_2CO_3 (1.45 g, 10.5 mmol, 2.5 eq) were dissolved in DMF (40 mL) and stirred for 16 hours at 60°C, using a heating mantle. The solvent was removed *in vacuo* and the residue was redissolved in EtOAc (50 mL). The organic layer was washed with H_2O (50 mL) and 5% $LiCl_{(aq)}$ (50 mL). The organic layer was dried ($MgSO_4$) and the solvent was removed *in vacuo*. The compound was purified by flash chromatography (SiO_2, 0-14% EtOAc in PetEther) to afford the product as a white crystalline solid (1.13 g, 3.03 mmol, 72 %).

Characterisation agreed with the literature data.[4,5]

^{1}H NMR (400 MHz, $CDCl_3$) δ 7.33 – 7.21 (m, 15H, C1H, C2H and C3H), 7.20 – 7.16 (m, 2H, C8H), 6.93 – 6.89 (m, 2H, C7H), 4.71 (d, *J* = 2.4 Hz, 2H, C10H_2), 2.57 (t, *J* = 2.4 Hz, 1H, C12H).

^{13}C NMR (101 MHz, $CDCl_3$) δ 155.8 (C9), 147.1 (C4), 140.1 (C6), 132.4 (C8), 131.3 (C3), 127.6 (C2), 126.0 (C1), 113.8 (C7), 78.8 (C11), 75.6 (C12), 64.5 (C5), 55.9 (C10).

FT-IR (ATR): v_{max}/cm^{-1} 3286, 3056, 3029, 2862, 2121, 1596, 1505, 1491, 1444, 1294, 1223, 1183, 1083, 1030, 908, 827, 730, 700, 629, 579, 524.

HRMS (ES$^+$): calculated for $C_{28}H_{23}O + H^+$ is 375.1749, found 375.1737.

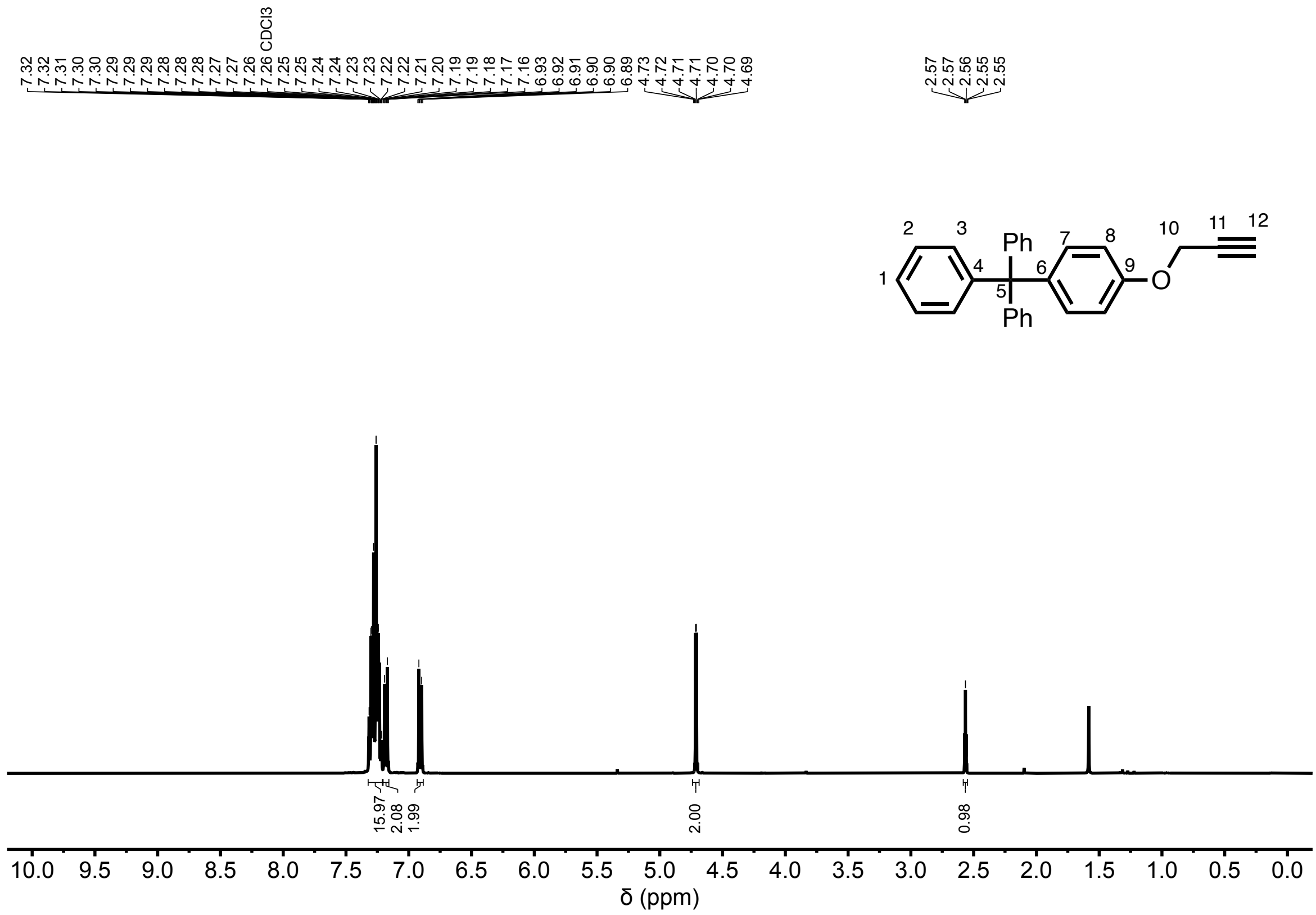

Figure S14 ^{1}H NMR (400 MHz, $CDCl_3$) spectrum of compound **2**.

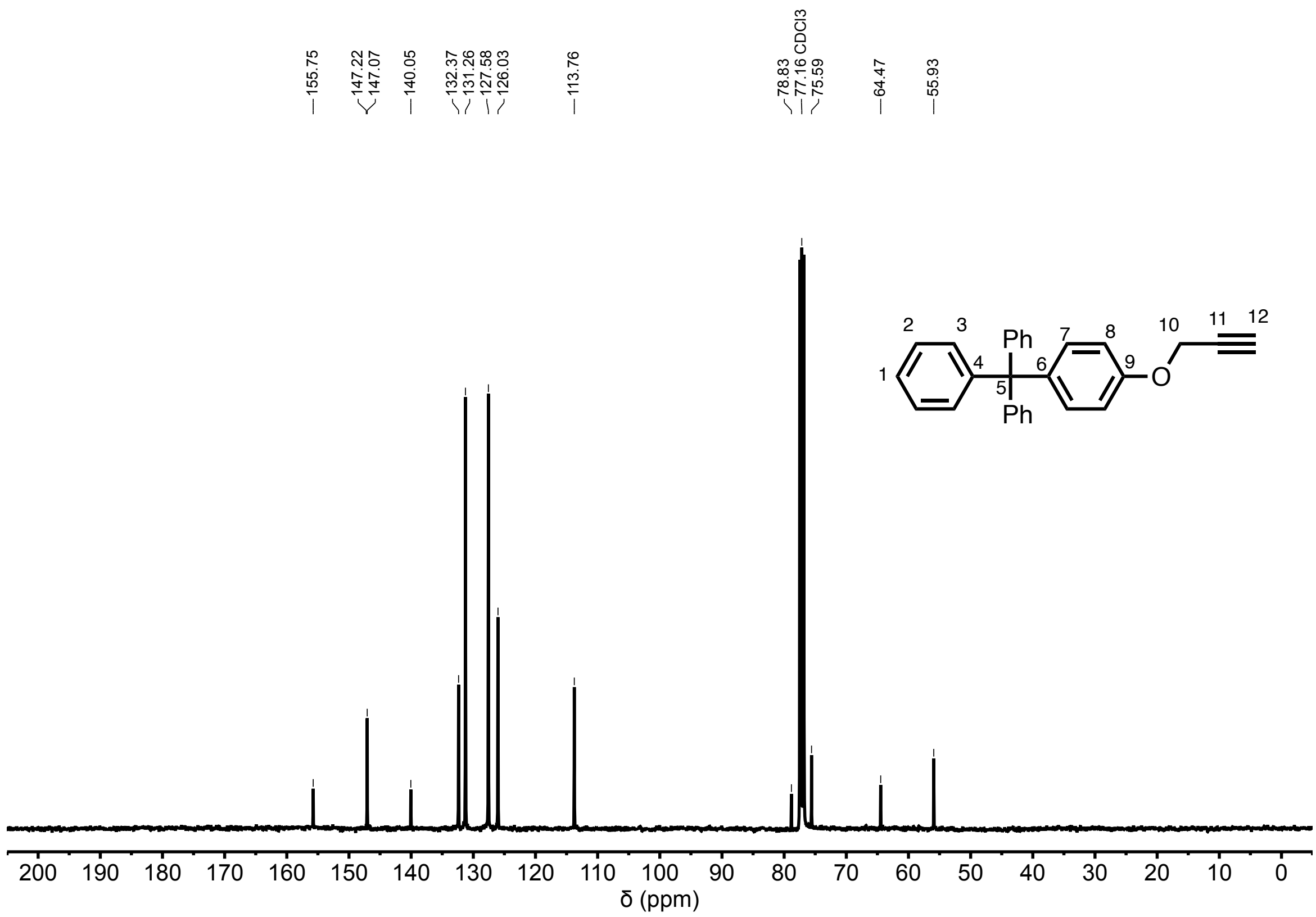

Figure S15 ^{13}C NMR (101 MHz, $CDCl_3$) spectrum of compound **2**.

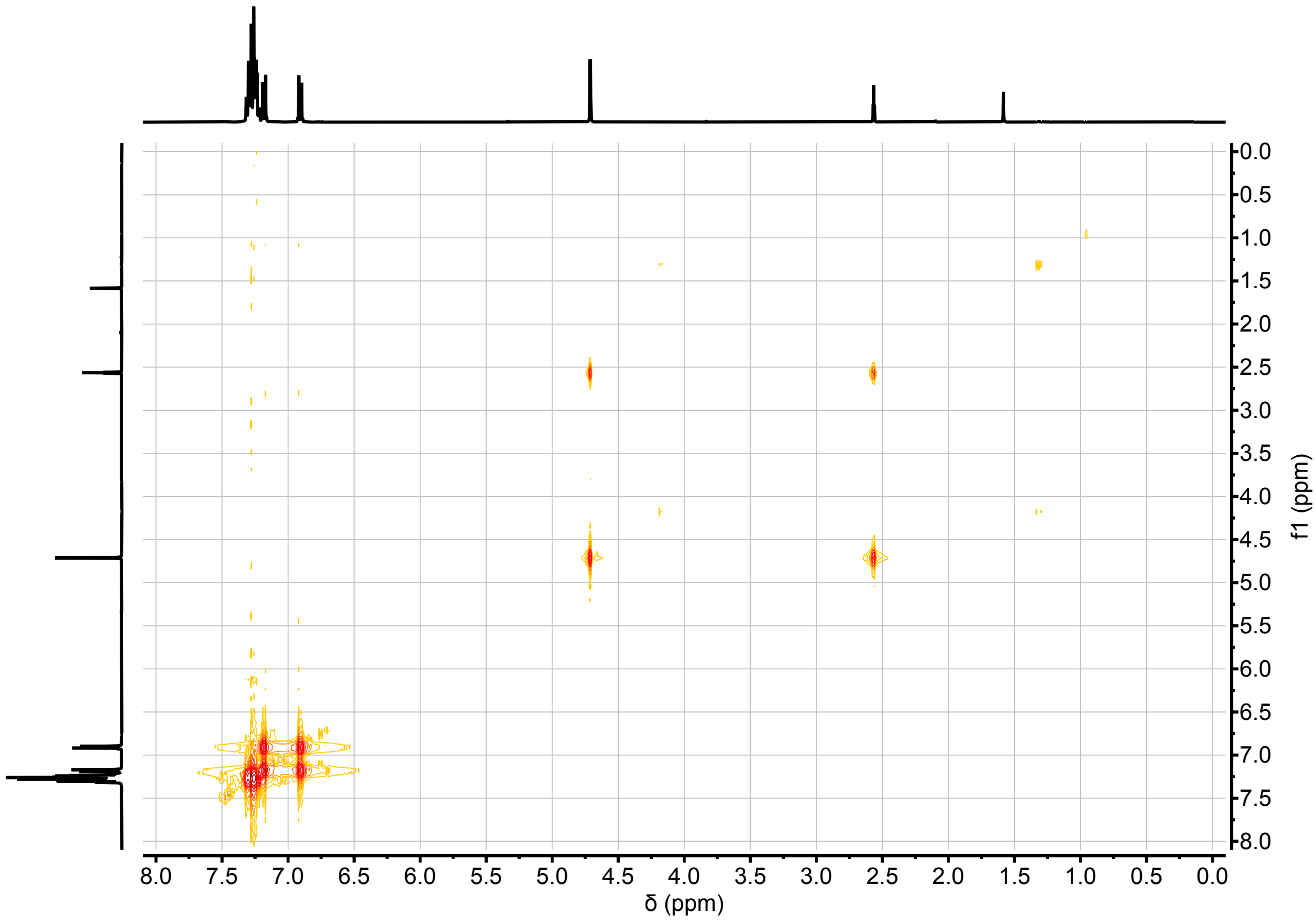

Figure S16 ^{1}H-^{13}C COSY NMR spectrum of compound **2**.

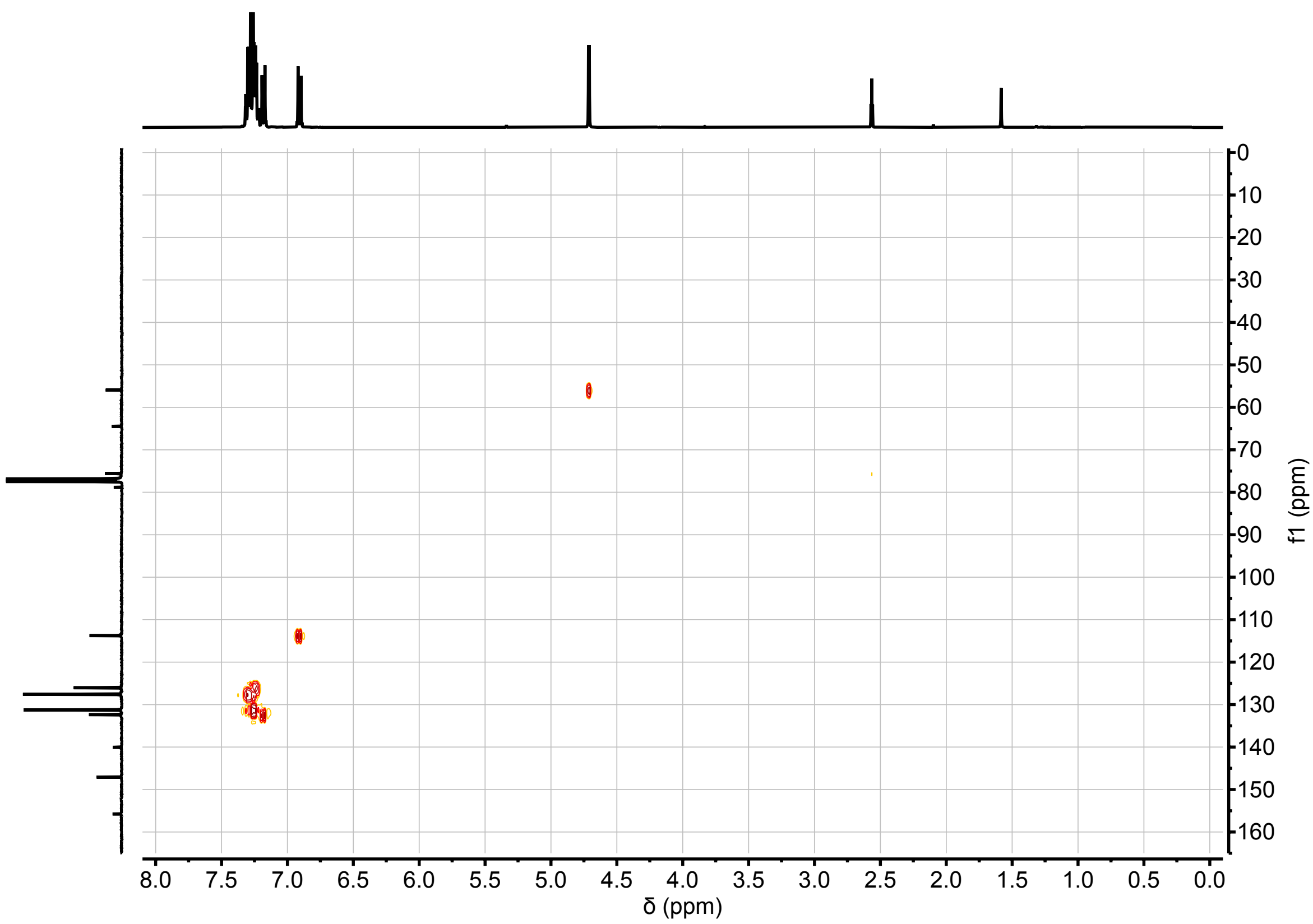

Figure S17 ^{1}H-^{13}C HSQC NMR spectrum of compound **2**.

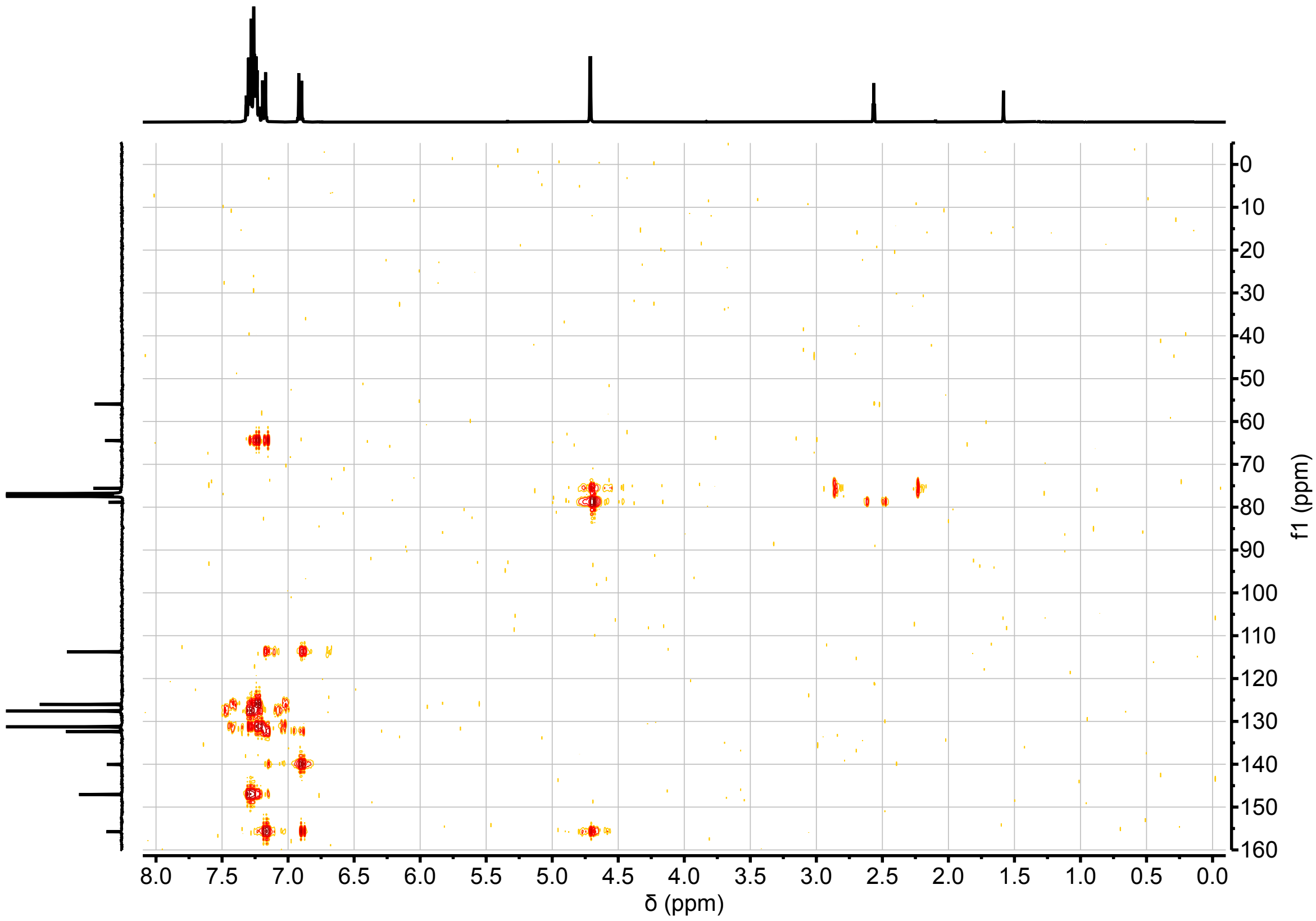

Figure S18 ^{1}H-^{13}C HMBC NMR spectrum of compound **2**.

2.1.4 Compound 8

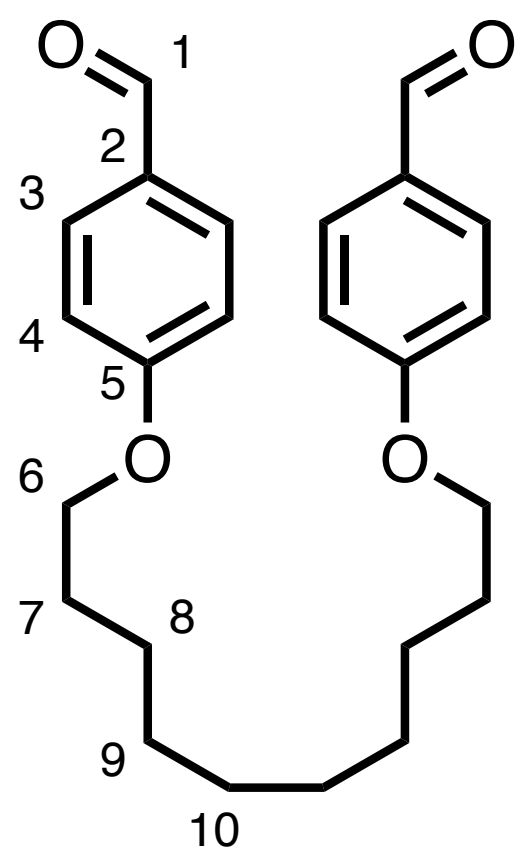

1,10-dibromodecane (2.00 g, 6.66 mmol, 1 eq), 4-hydroxybenzaldehyde (1.62 g, 13.3 mmol, 2 eq), K_2CO_3 (9.20 g, 66.6 mmol, 10 eq) and a catalytic amount of sodium iodide (100 mg) was stirred under a N_2 atmosphere in DMF for 18 hours at 70°C, using a heating mantle. The crude product was recrystallised in MeOH to yield the product as a white solid (1.9146 g, 5.01 mmol, 75 %).

Characterisation agreed with the literature data.[3]

^{1}H NMR (400 MHz, $CDCl_3$) δ 9.87 (s, 2H, C1<u>H</u>), 7.86 – 7.78 (m, 4H, C4<u>H</u>), 7.02 – 6.94 (m, 4H, C3<u>H</u>), 4.03 (t, *J* = 6.5 Hz, 4H, C6$\underline{H_2}$), 1.81 (dq, *J* = 8.2, 6.5 Hz, 4H, C7$\underline{H_2}$), 1.51 – 1.29 (m, 12H, C8-10$\underline{H_2}$).

^{13}C NMR (101 MHz, $CDCl_3$) δ 190.9 (C1), 164.4 (C5), 132.1 (C4), 129.9 (C2), 114.9 (C3), 68.5 (C6), 29.6 (C7), 29.4 (C8), 29.2 (C9), 26.1 (C10).

FT-IR (ATR): ν_{max}/cm^{-1} 2941, 2918, 2851, 1685, 1594, 1509, 1472, 1314, 1264, 1212, 1154, 1108, 1044, 1003, 856, 834, 790, 725, 640, 614, 515.

HRMS (ES$^+$): calculated for $C_{24}H_{30}O_4 + H^+$ is 383.2232, found 383.2222.

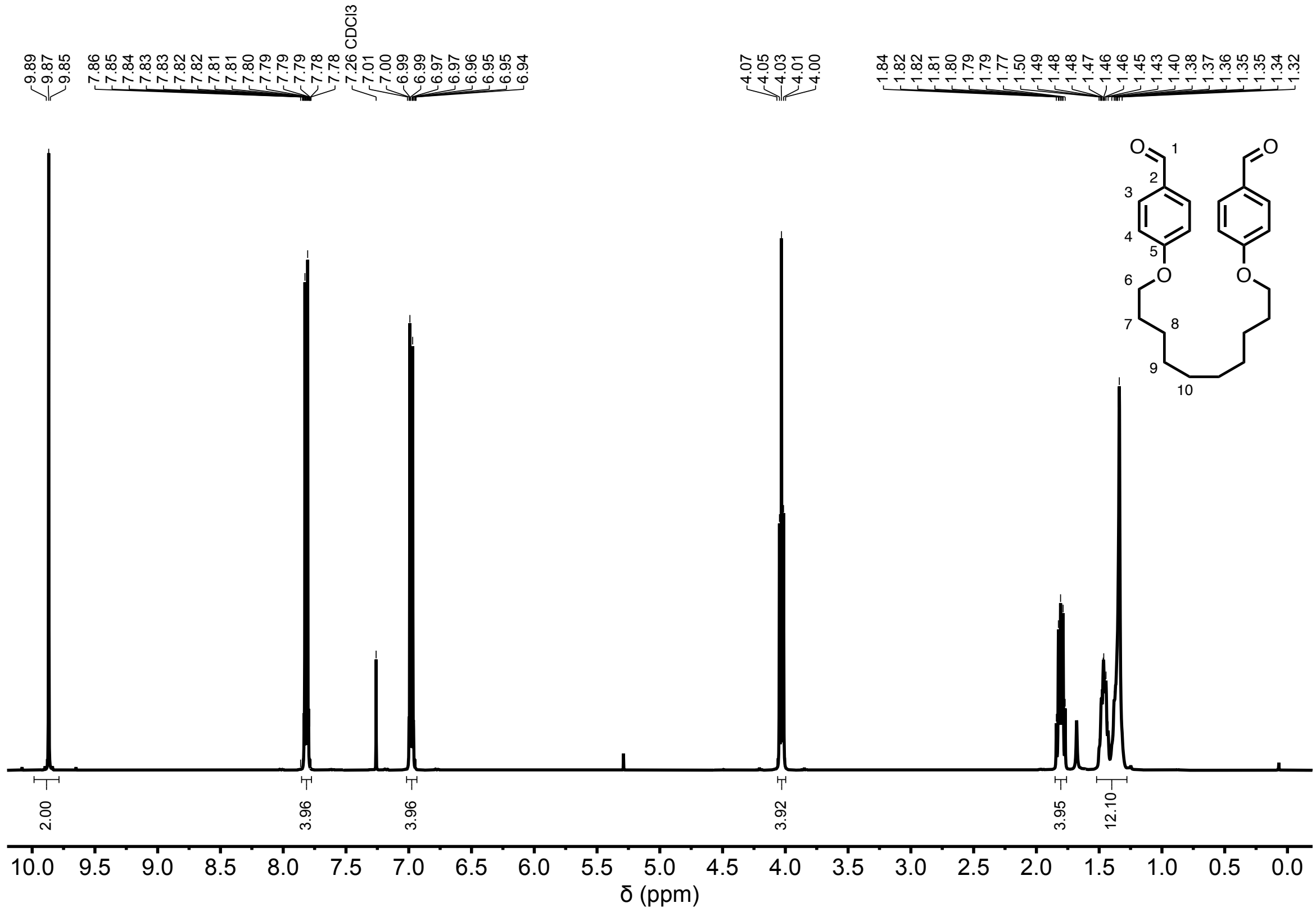

Figure S19 ^{1}H NMR (400 MHz, $CDCl_3$) spectrum of compound **8.**

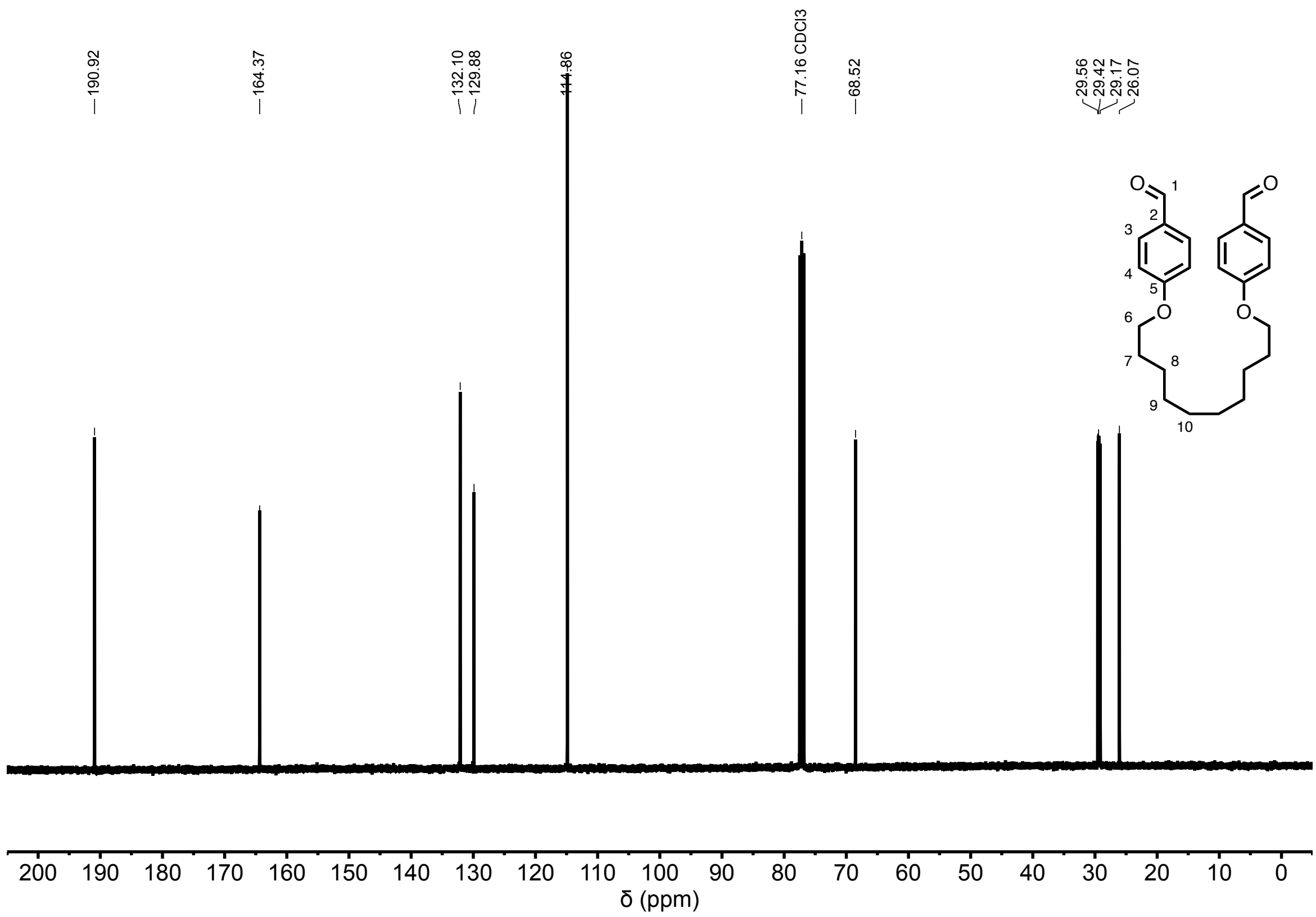

Figure S20 ^{13}C NMR (101 MHz, $CDCl_3$) spectrum of compound **8.**

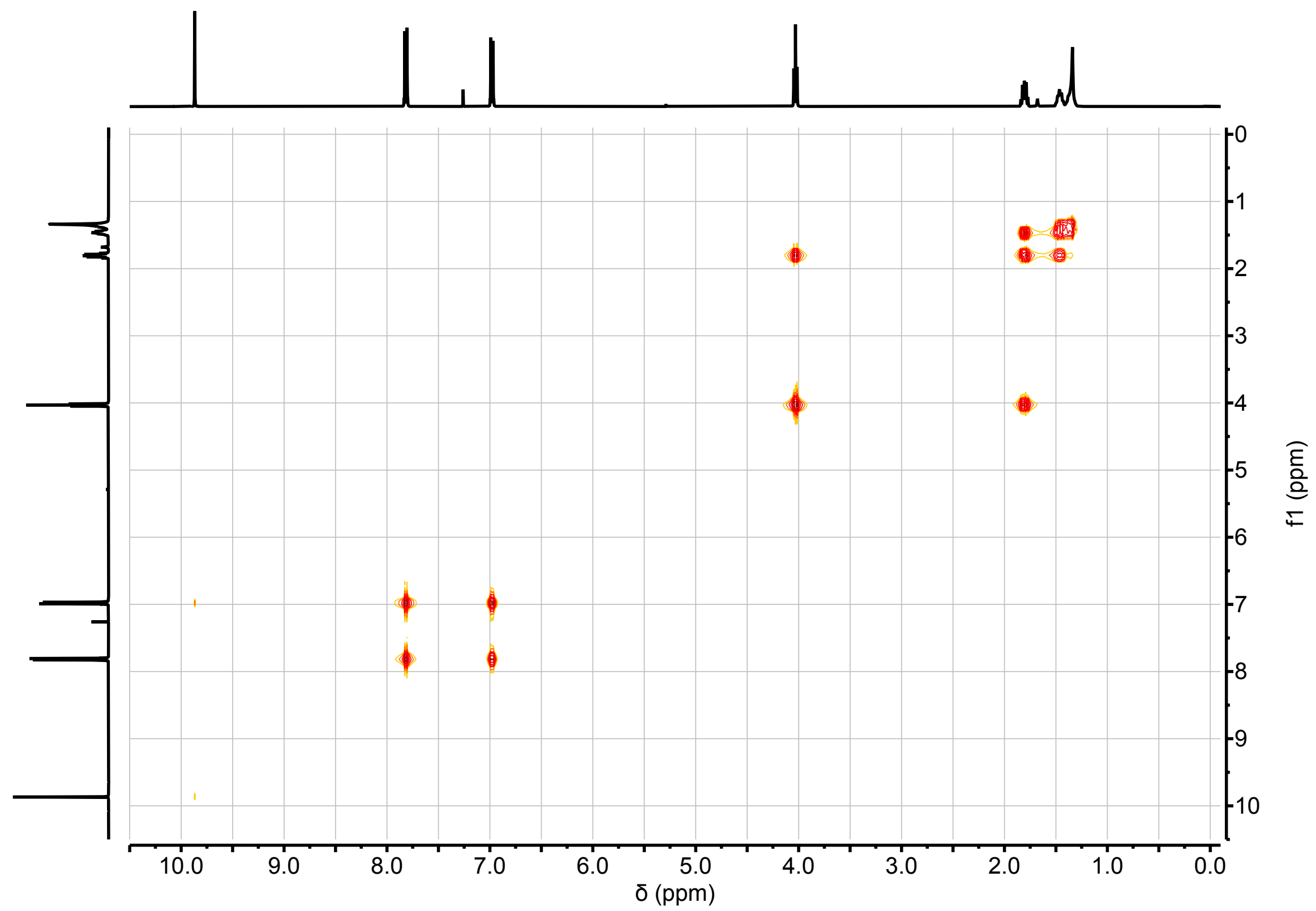

Figure S21 ¹H-¹³C COSY NMR spectrum of compound **8**.

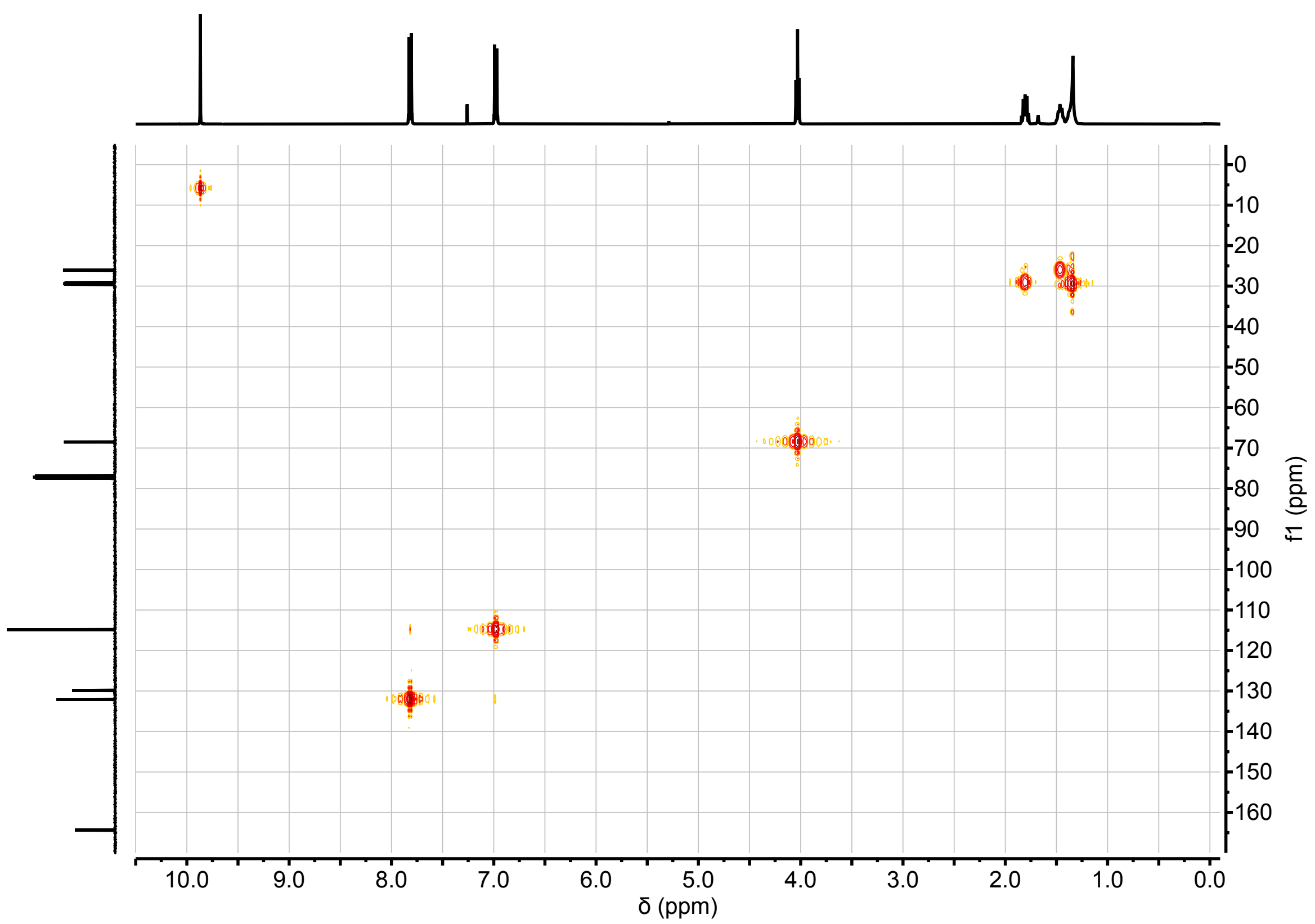

Figure S22 ¹H-¹³C HSQC NMR spectrum of compound **8**.

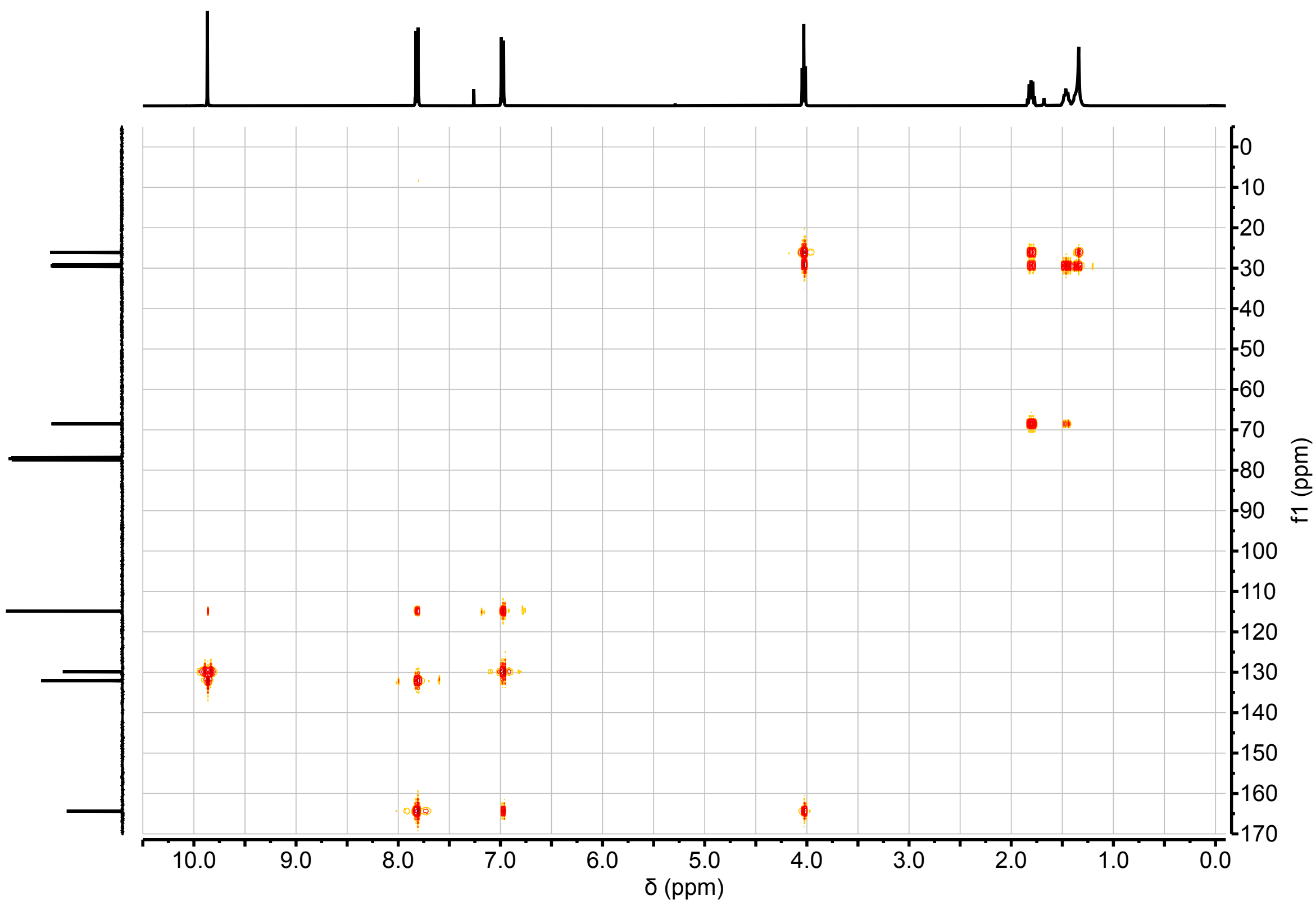

Figure S23 ^{1}H-^{13}C HMBC NMR spectrum of compound **8**.

2.1.5 Compound 3

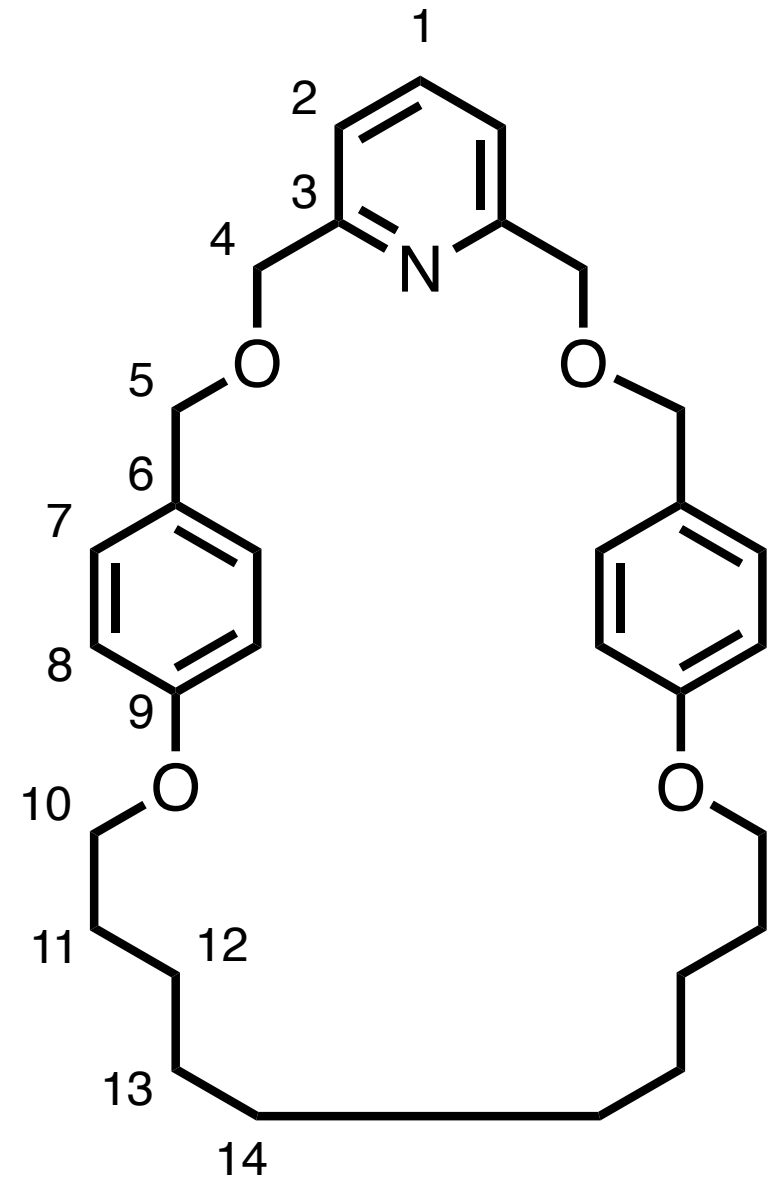

3 (0.5019 g, 1.31 mmol, 1 eq) was dissolved in $CHCl_3$/MeOH (5:1, 120 mL) followed by addition of $NaBH_4$ (0.50 g, 13.2 mmol, 10 eq) in small portions. The mixture was stirred for 1 hour and followed by the addition of 1M $HCl_{(aq)}$ (200 mL). The product precipiated and was collected by vacuum filtration and washed with H_2O (100 mL). The crude product (0.4039 g, 1.04 mmol) was used in the next step without futher purification.

The crude product (0.4039 g, 1.04 mmol) and 2,6-bis(bromomethyl)pyridine (276.0 mg, 1.04 mmol were dissolved in dry THF (300 mL) under an N_2 atmosphere. Small potions of NaH (200 mg, 8.3 mmol, 8 eq) and the resulting mixture was heated under reflux, by a heating mantle, for 5 days. Upon cooling the suspension was filtered and the solvent was removed *in vacuo*. The residue was redissolved in DCM (100 mL) and washed with H_2O (100 mL). The mixture was purified by flash column chromatography (SiO_2, 0-14 % EtOAc in DCM) to afford the product as a white solid (0.2885 g, 0.58 mmol, 31 %).

^{1}H NMR (700 MHz, $CDCl_3$) δ 7.69 (t, *J* = 7.7 Hz, 1H, C1H), 7.36 (d, *J* = 7.7 Hz, 2H, C2H), 7.25 – 7.22 (m, 4H, C8H), 6.84 – 6.80 (m, 4H, C7H), 4.60 (s, 4H, C4H_2), 4.43 (s, 4H, C5H_2), 3.97 (t, *J* = 6.3 Hz, 4H, C10H_2), 1.73 (p, *J* = 6.7 Hz, 4H, C11H_2), 1.43 (p, *J* = 7.2 Hz, 4H, C12H_2), 1.29 (d, *J* = 3.1 Hz, 8H, C13H_2 and C14H_2).
^{13}C NMR (176 MHz, $CDCl_3$) δ 158.9 (C9), 157.9 (C3), 137.2 (C1), 130.2 (C8), 129.5 (C6), 120.1 (C2), 114.7 (C7), 72.4 (C4), 71.4 (C5), 67.5 (C10), 29.5 (C11), 28.8 (C12), 28.7 (C13), 25.7 (C14).

FT-IR (ATR): v_{max}/cm^{-1} 2926, 2855, 1698, 1611, 1511, 1457, 1360, 1301, 1244, 1173, 1095, 825, 590.

HRMS (ES^+): calculated for $C_{31}H_{40}NO_4 + H^+$ is 490.2957, found 490.2971.

Characterisation agreed with the literature data.[2]

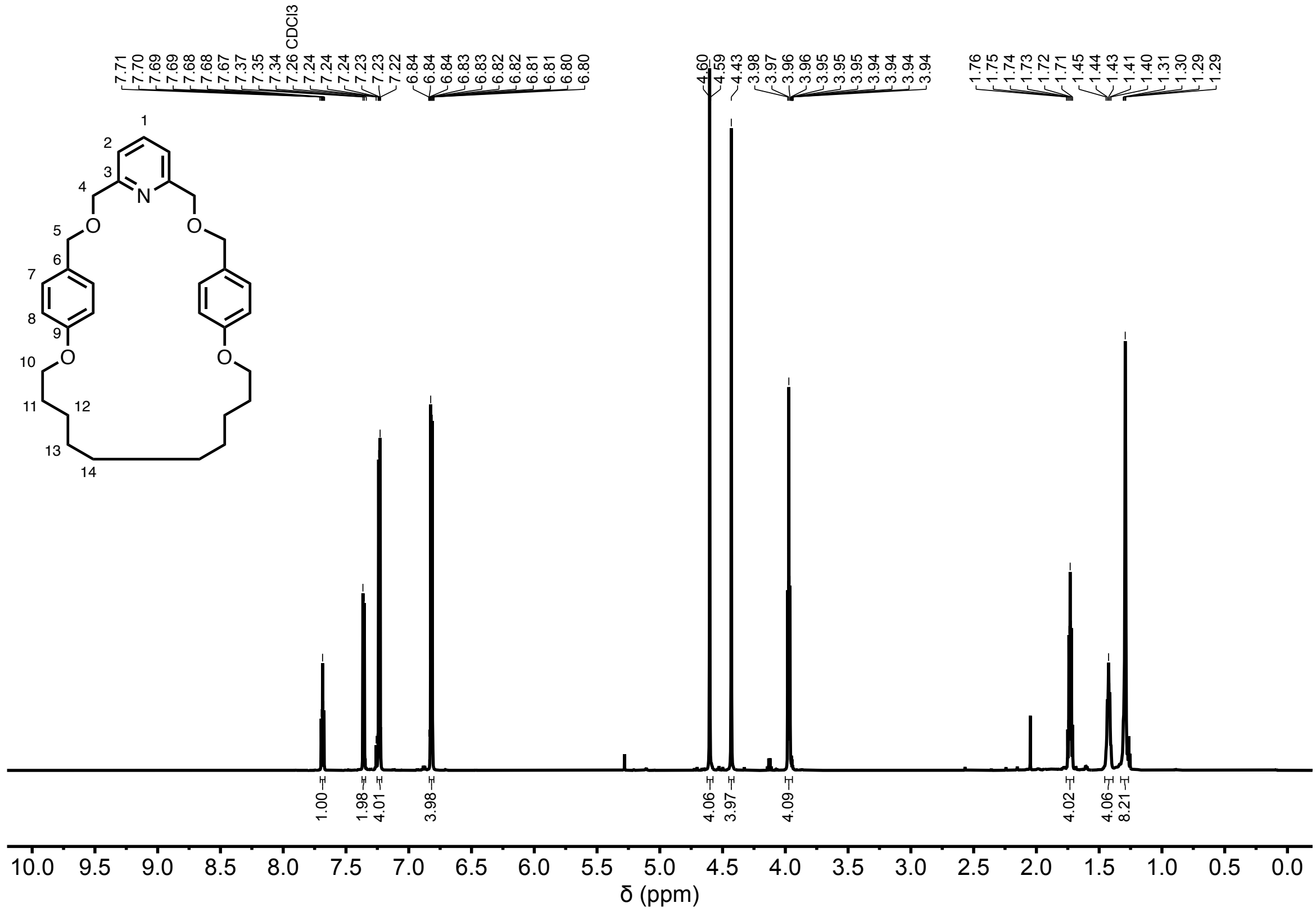

Figure S24 ^{1}H NMR (700 MHz, $CDCl_3$) spectrum of compound **3**.

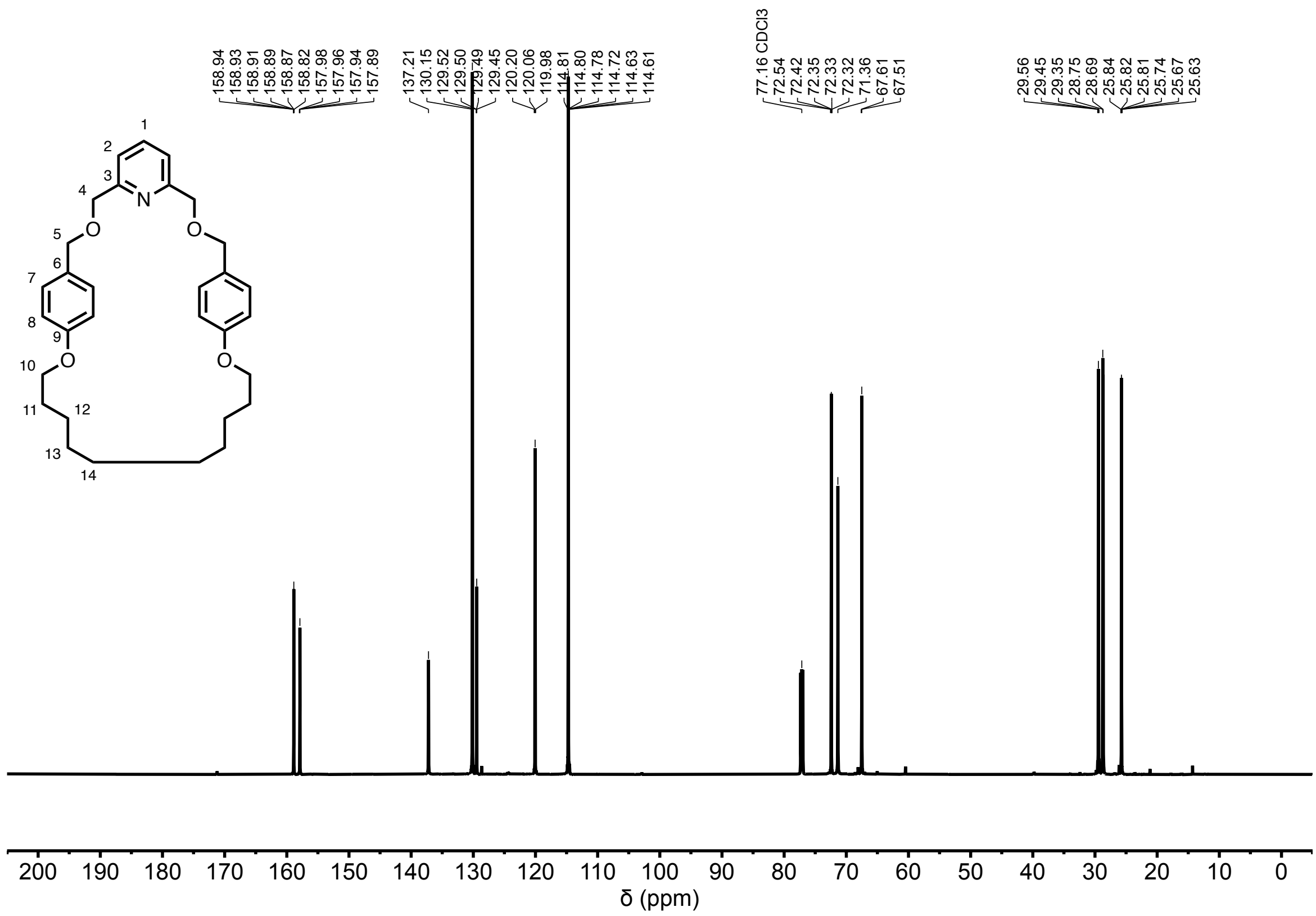

Figure S25 ^{13}C NMR (176 MHz, $CDCl_3$) spectrum of compound **3**.

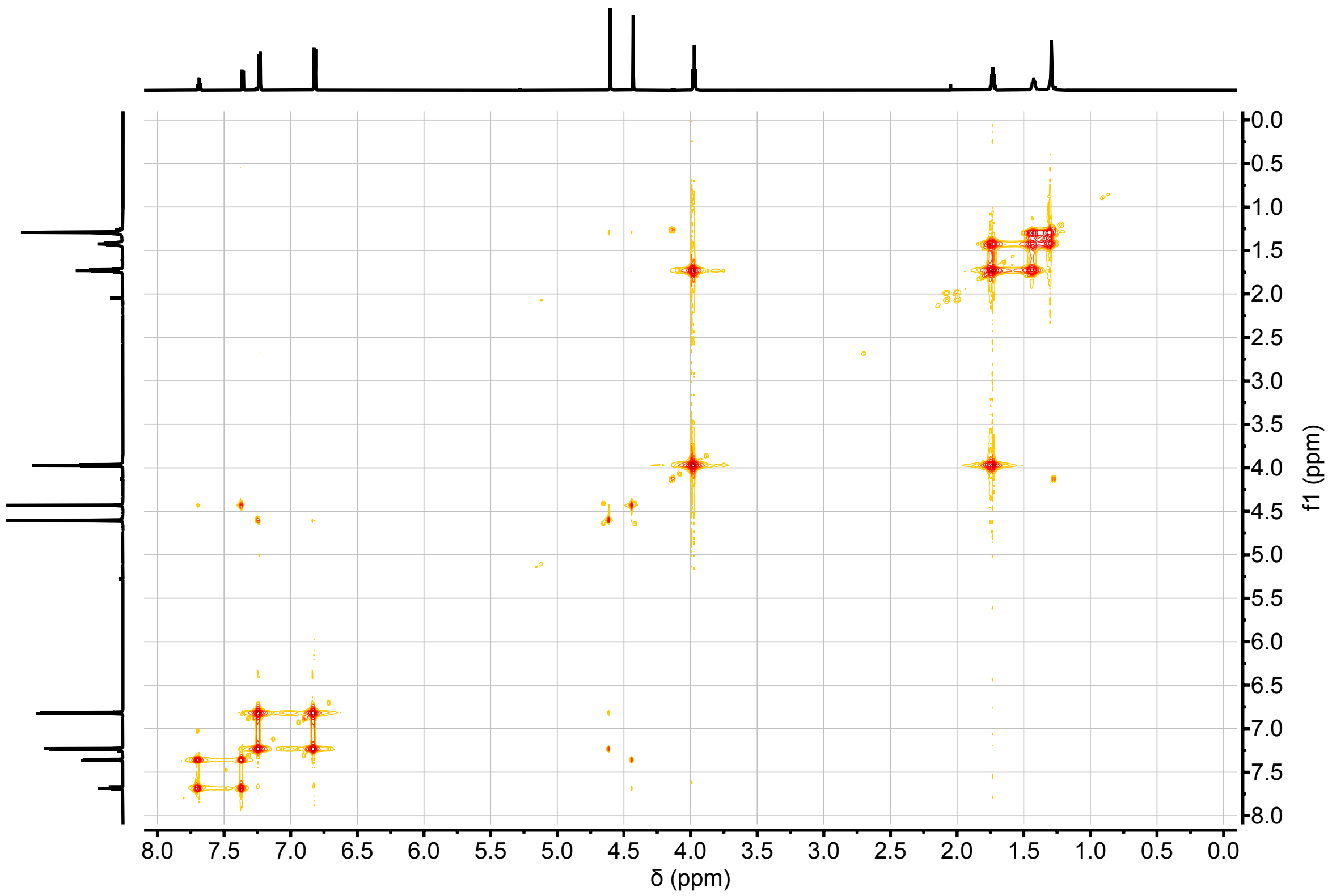

Figure S26 ¹H-¹³C COSY NMR spectrum of compound **3**.

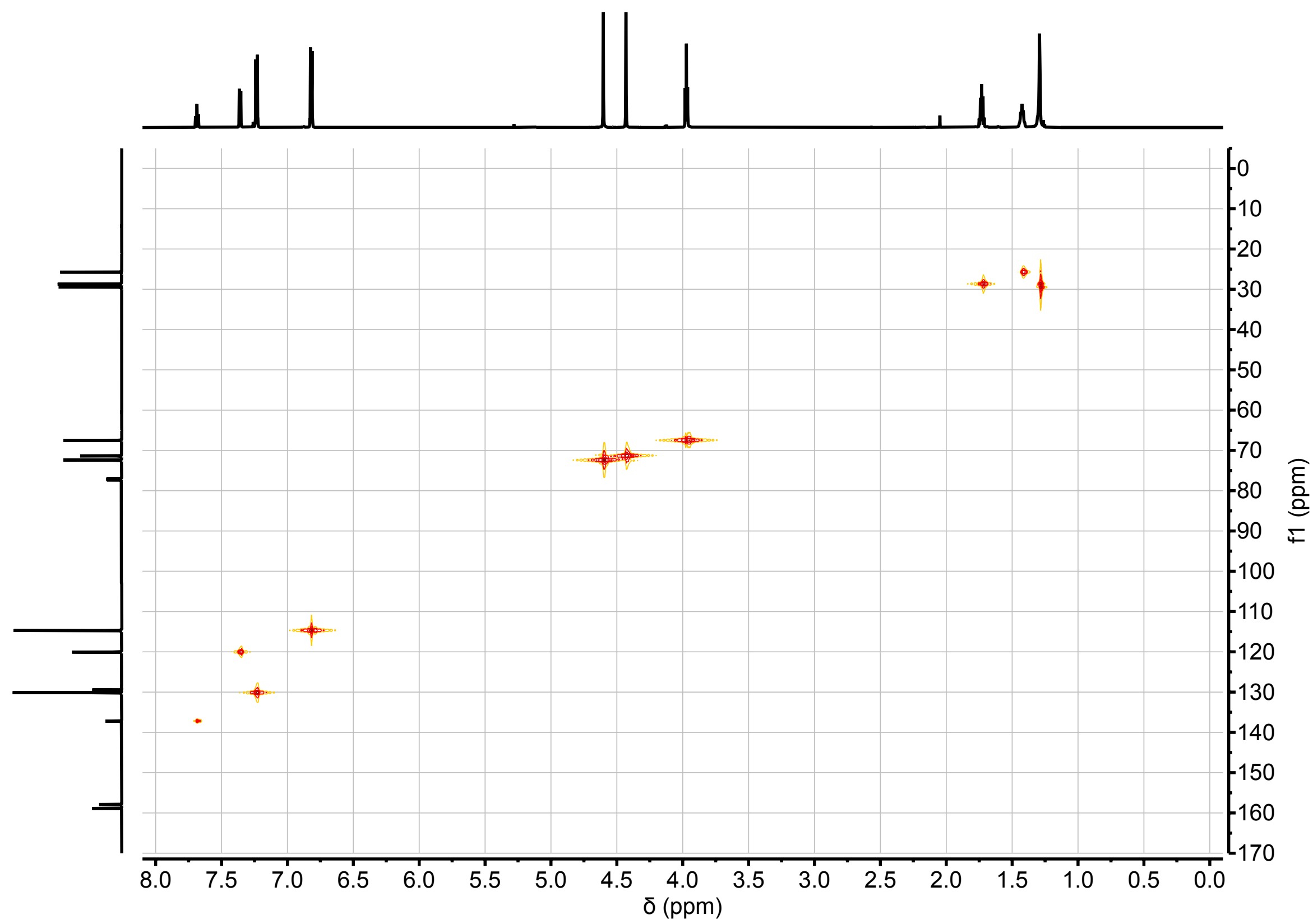

Figure S27 ¹H-¹³C HSQC NMR spectrum of compound **3**.

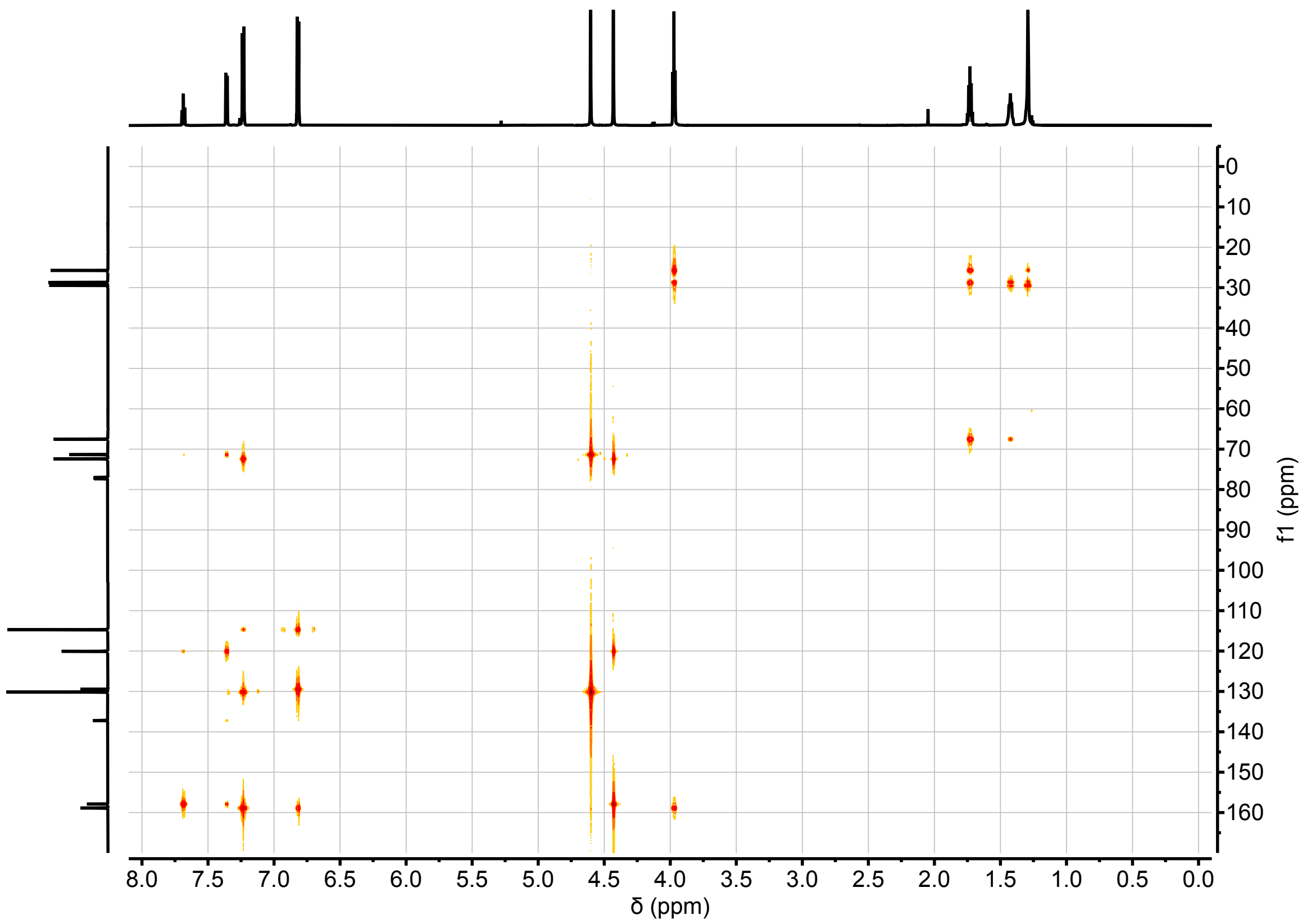

Figure S28 ^{1}H-^{13}C HMBC NMR spectrum of compound **3**.

2.2 AT-CuAAC Product Characterisation

2.2.1 Compound 4 (axle)

A reference sample of compound **4** was prepared and characterised (below). The HPLC chromatogram and ESI(+) mass spectrum of the reference compound enabled the identification of **4** as a product of AT-CuAAC

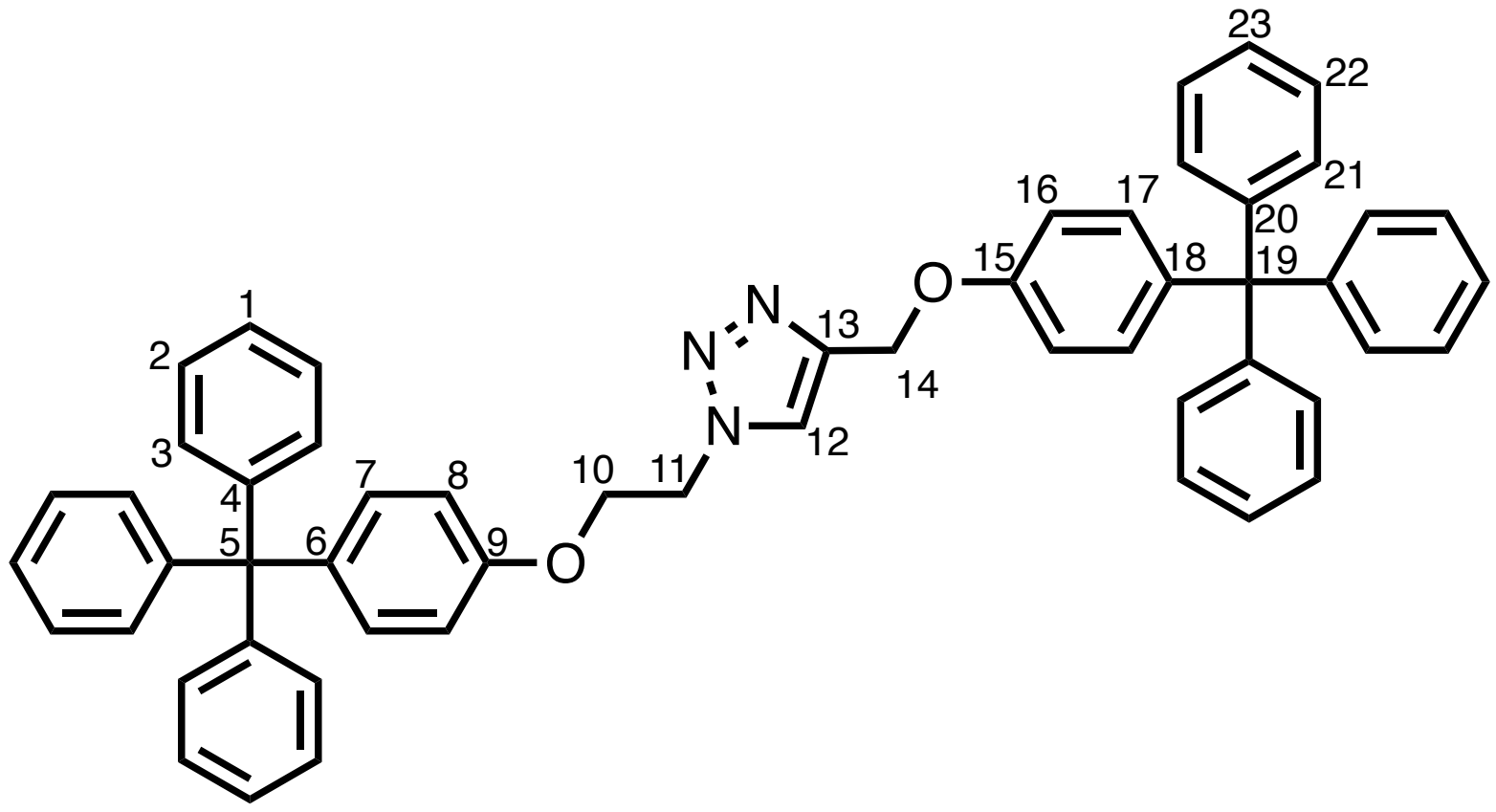

Azide **1** (0.20 g, 0.49 mmol, 1 eq), alkyne **2** (0.18 g, 0.49 mmol, 1 eq) and Cu(I)MeCN$_4$]PF$_6$ (0.18 g, 0.49 mmol, 1 eq) were dissolved in DMF (5 mL). The resulting mixture was heated in a microwave reactor at 90 °C for 1 hour. The mixture was cooled to 20 °C and H_2O (30 mL) was added. The solution was extracted with EtOAc (3 × 30 mL) and the combined organic layers were washed with H_2O (3 × 30 mL) and 0.1 M $LiCl_{(aq)}$ (30 mL). The organic layer was then dried ($MgSO_4$) and the solvent was removed *in vacuo*. The mixture was purified by flash column chromatography (SiO_2, 0-50 % EtOAc in Pet. Ether) to afford the product as a white amorphous solid (0.117 g, 0.15 mmol, 31 %).

^{1}H NMR (700 MHz, $CDCl_3$) δ 7.83 (s, 1H, C12$\underline{H}_1$), 7.29 – 7.19 (m, 32H, C1$\underline{H}_1$, C2$\underline{H}_1$, C3$\underline{H}_1$, C21$\underline{H}_1$, C22$\underline{H}_1$, and C23$\underline{H}_1$), 7.17 (overlapping d, *J* = 9.0 Hz, 4H, C7$\underline{H}_1$, and C17$\underline{H}_1$), 6.91 (d, *J* = 9.0 Hz, 2H, C18$\underline{H}_1$), 6.77 (d, *J* = 9.0 Hz, 2H, C8$\underline{H}_1$), 5.21 (s, 2H, C14$\underline{H}_2$), 4.73 (t, *J* = 5.0 Hz, 2H, C10$\underline{H}_2$), 4.33 (t, *J* = 5.0 Hz, 2H, C11$\underline{H}_2$).

^{13}C NMR (176 MHz, $CDCl_3$) δ 156.4 (C18), 155.8 (C6), 147.0 (C20), 146.9 (C4), 144.3 (C13), 140.2 (C18), 139.7 (C6), 132.4 (C17), 132.3 (C7), 131.2 (C21), 131.1 (C3), 127.6 (C22), 127.5 (C2), 126.0 (C23), 127.0 (C1), 124.0 (C12), 113.6 (C16), 113.4 (C8), 66.2 (C11), 64.4 (C5 and C19), 62.0 (C14), 49.9 (C10).

FT-IR (ATR): v_{max}/cm^{-1} 1607, 1507, 1492, 1445, 1294, 1243, 1185, 1046, 1035, 1015, 903, 828, 724, 700, 648, 629, 595, 544, 523, 506.

HRMS (ES^+): calculated for $C_{55}H_{46}N_3O_2$ + H^+ is 780.3590, found 780.3575.

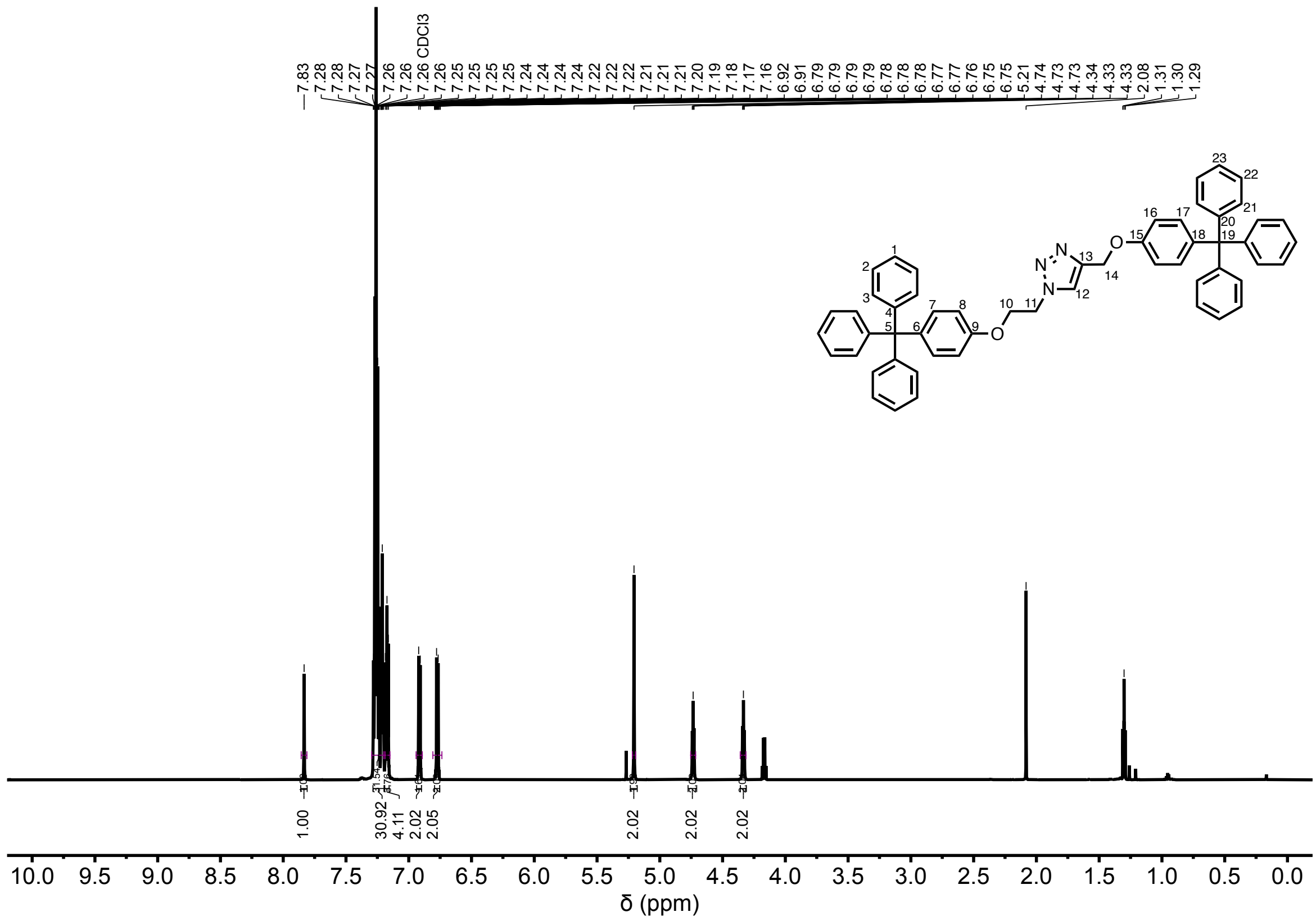

Figure S29 ^{1}H NMR (700 MHz, $CDCl_3$) spectrum of compound **4**.

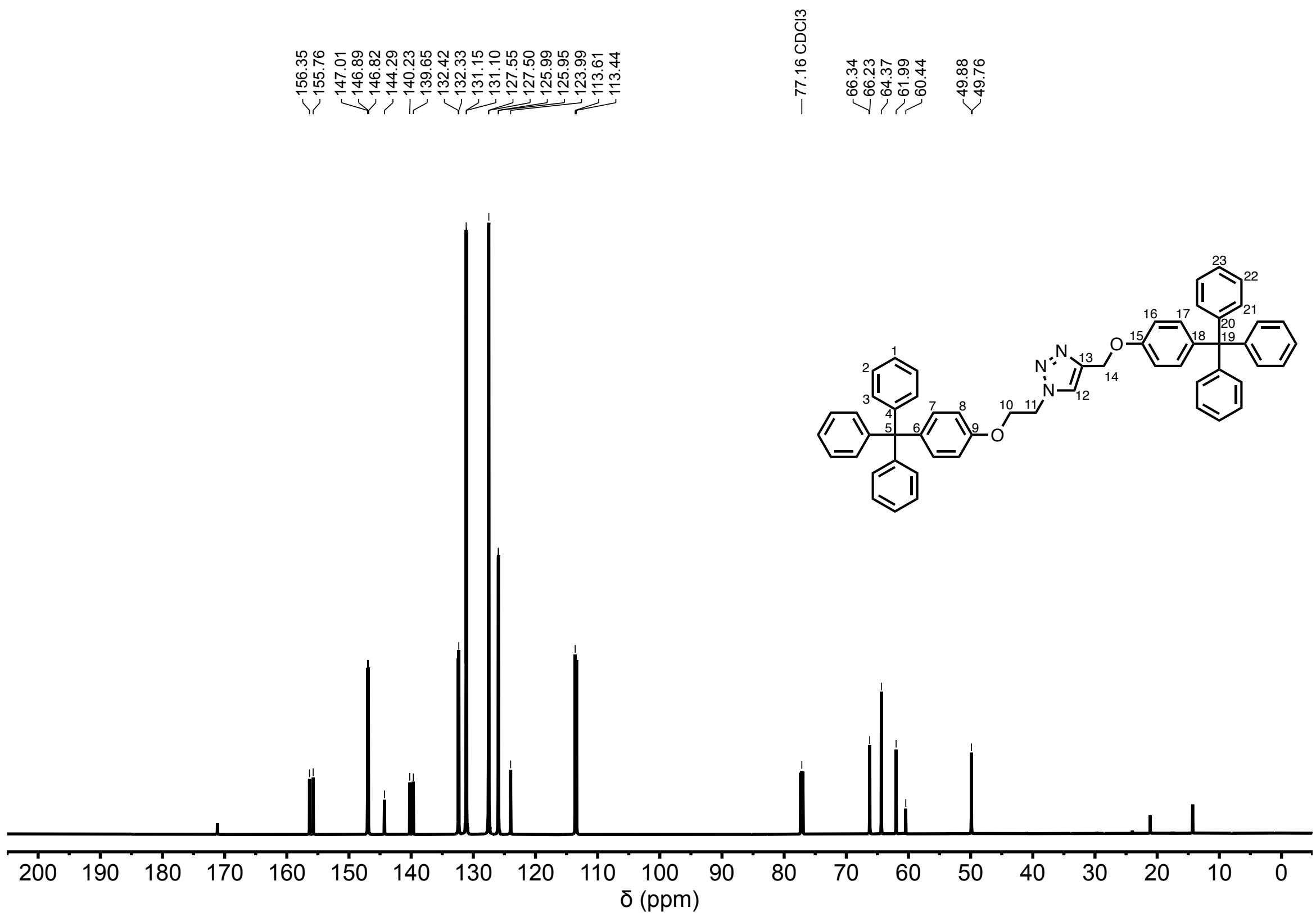

Figure S30 ^{13}C NMR (176 MHz, $CDCl_3$) spectrum of compound **4**.

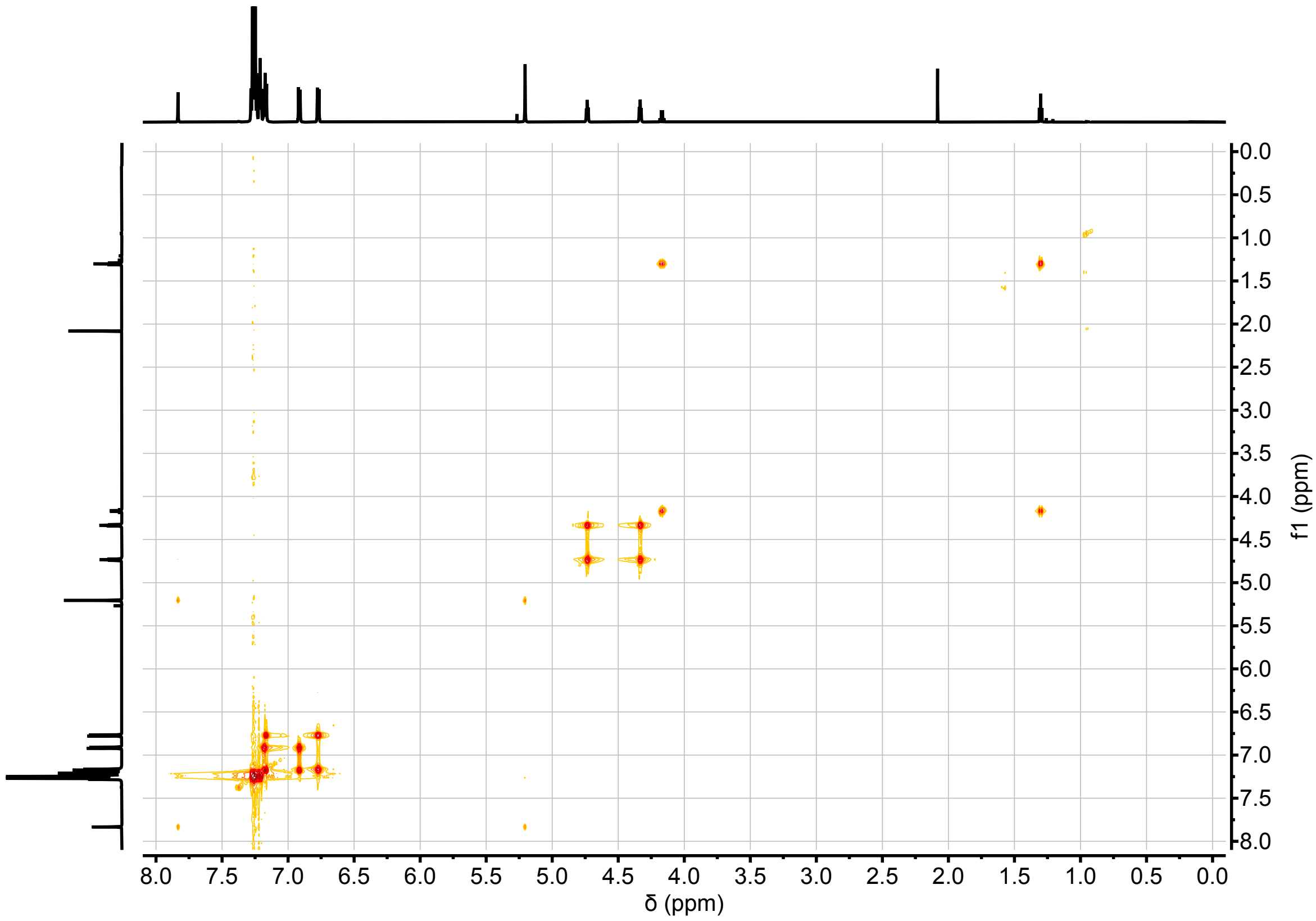

Figure S31 ¹H-¹³C COSY NMR spectrum of compound **4**.

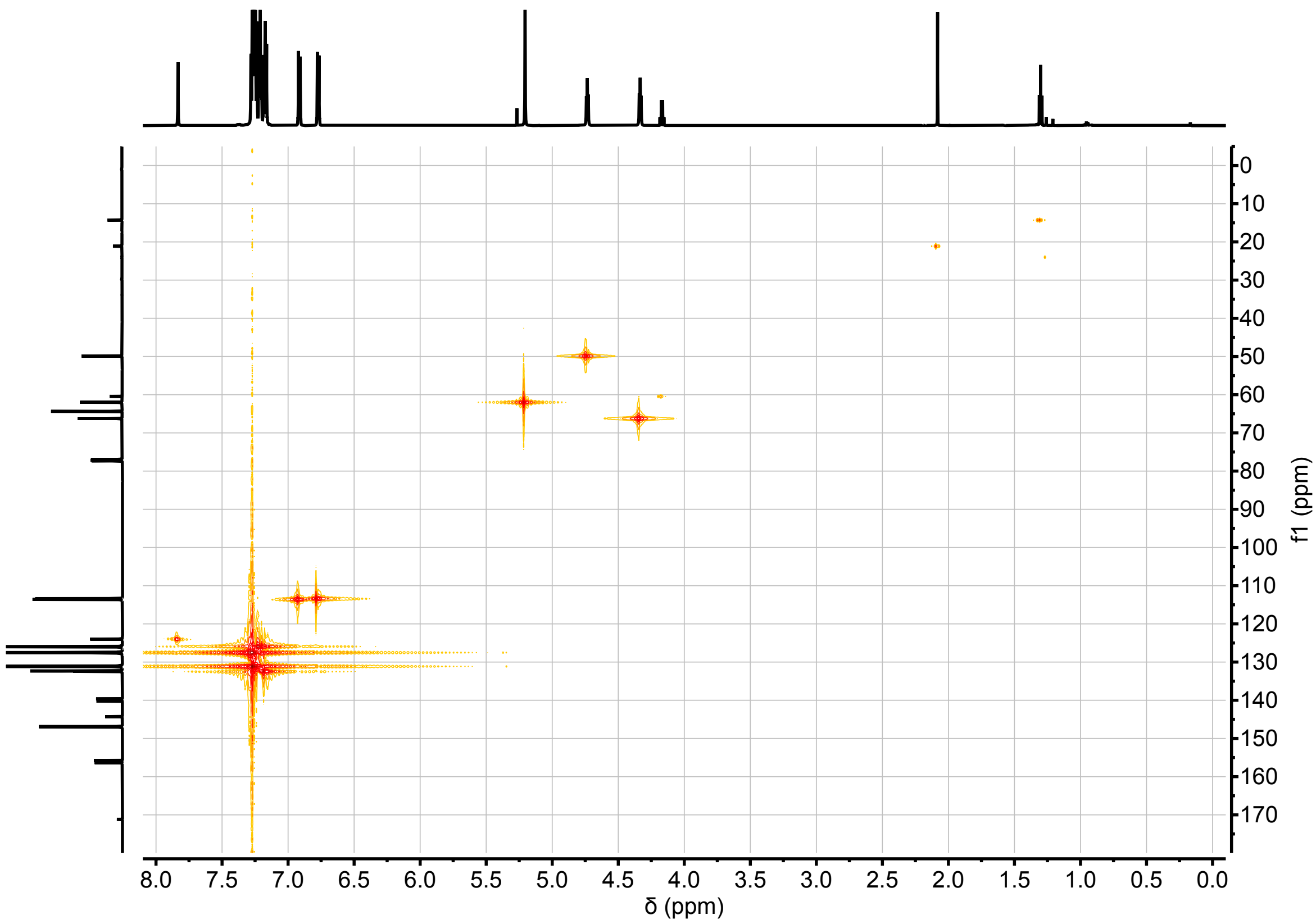

Figure S32 ¹H-¹³C HSQC NMR spectrum of compound **4**.

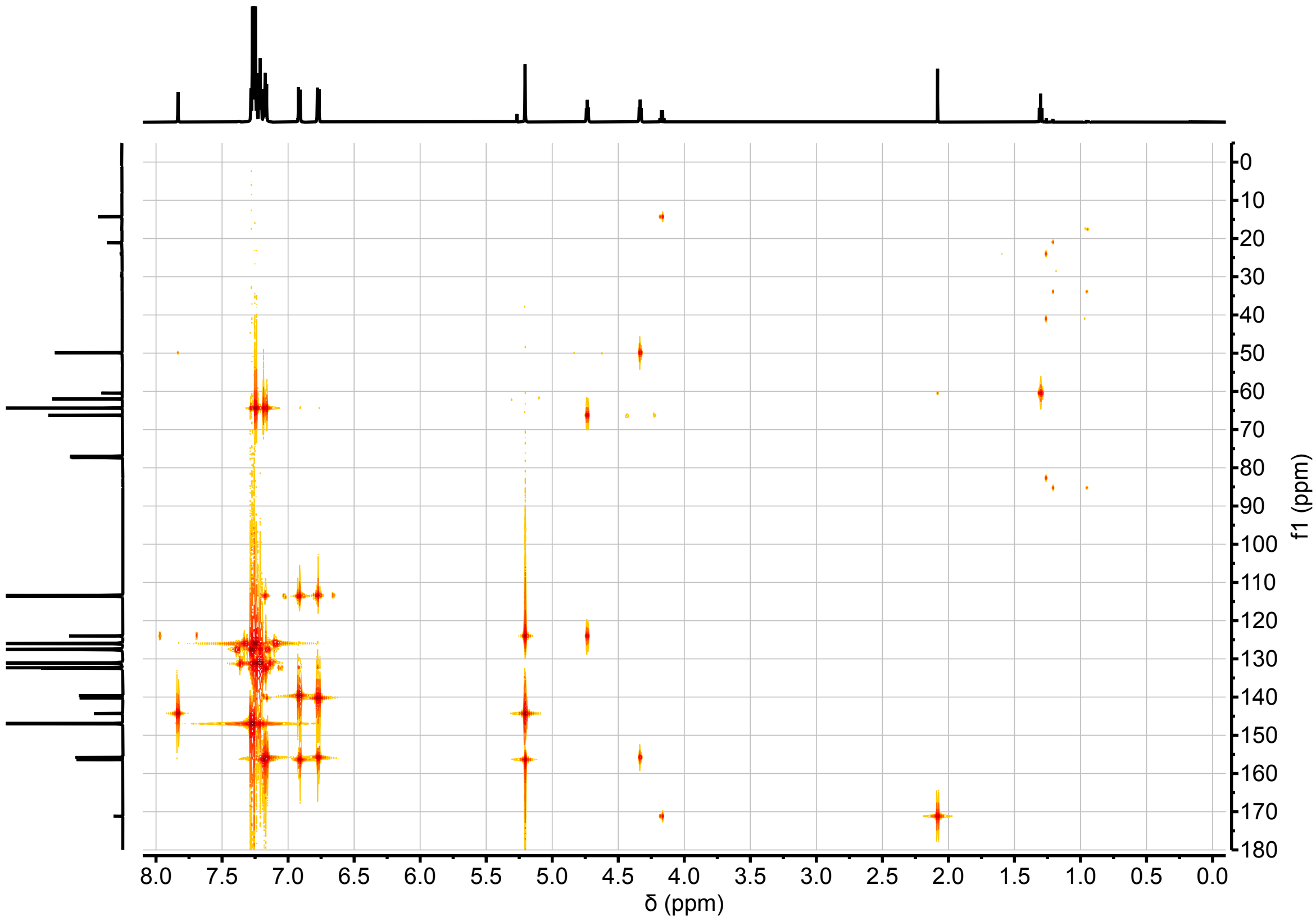

Figure S33 ^{1}H-^{13}C HMBC NMR spectrum of compound **4**.

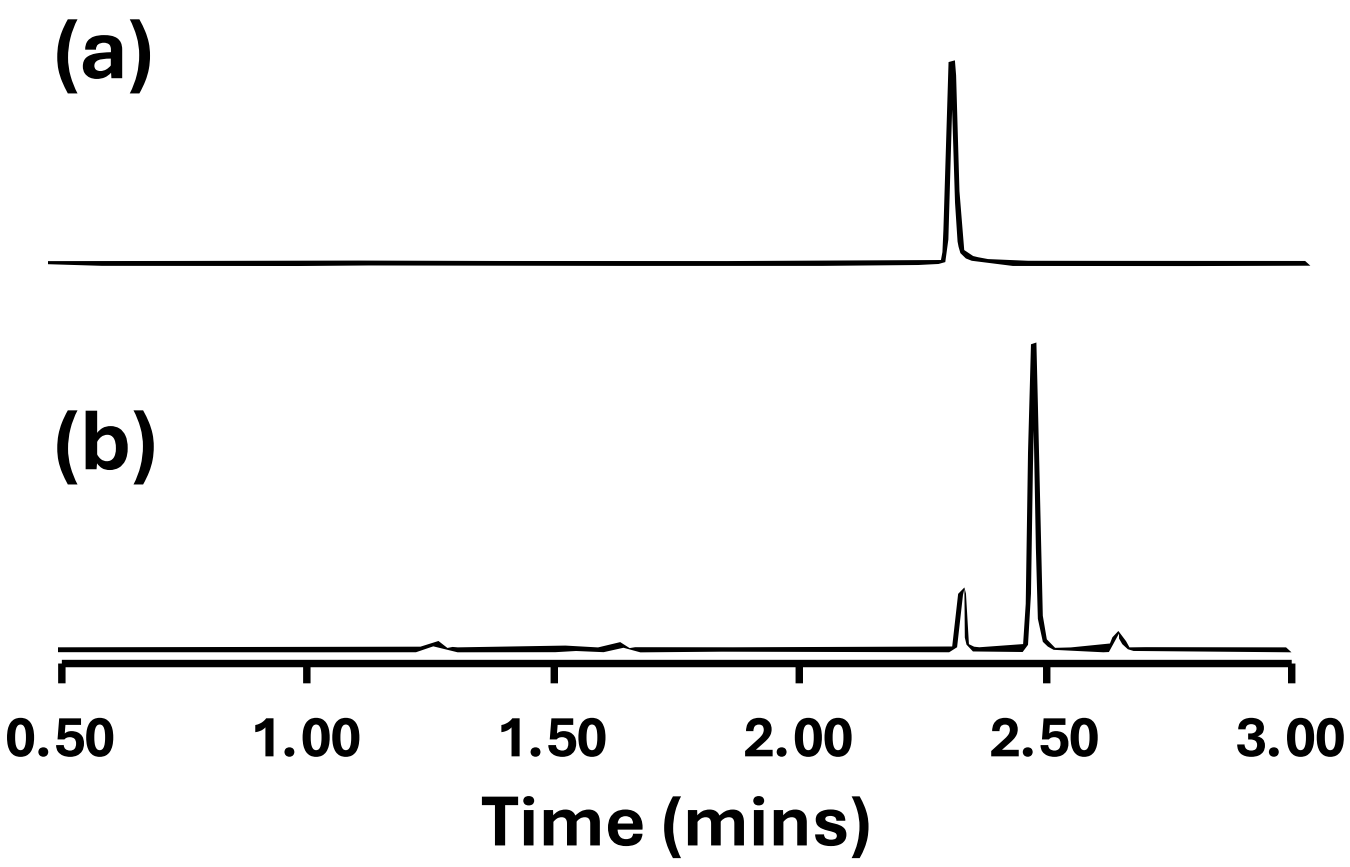

Figure S34 UPLC traces of the unthreaded axle **4**; (a) axle **4** synthesised directly as a reference compound; (b) axle **4** formed during the AT-CuAAC reaction described in Entry 2. UPLC conditions: C4 column at 40 °C using a gradient of 45–65% of MeCN/formic acid (0.1%) in water/formic acid (0.1%) over 1.5 min, then 65-100% MeCN/formic acid (0.1%) over 0.1 min, and then 100% MeCN/formic acid (0.1%) for 1.5 min. The UV–vis absorbance at 254 nm is plotted.

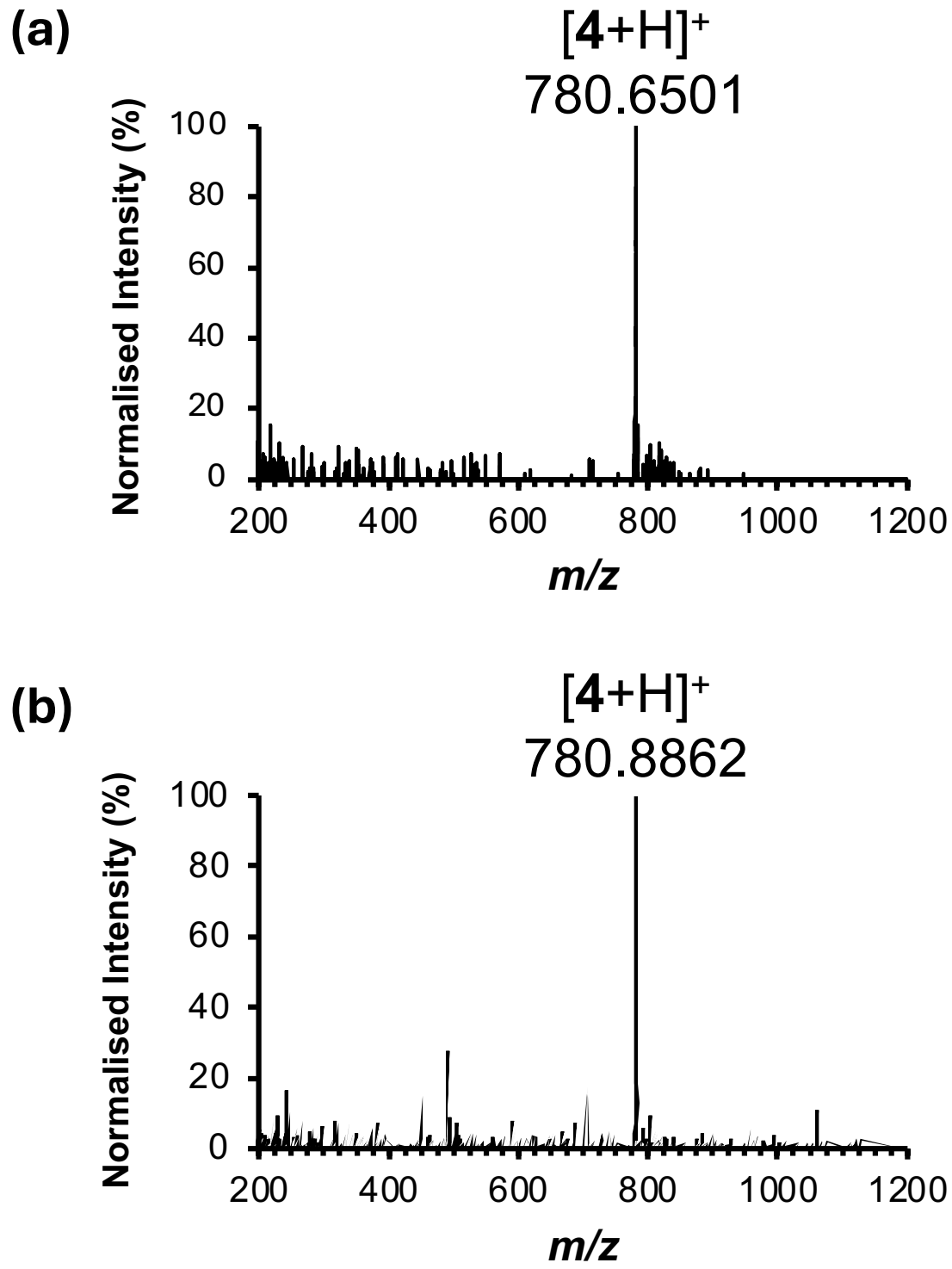

Figure S35 ESI(+) mass spectra of the unthreaded axle **4**. Calculated mass (ESI+): 780.3590 [**4**+H]+; (a) axle **4** synthesised directly as a reference compound; (b) axle **4** formed during the AT-CuAAC reaction described in Entry 2.

2.2.2 Compound 5 ([2]-rotaxane)

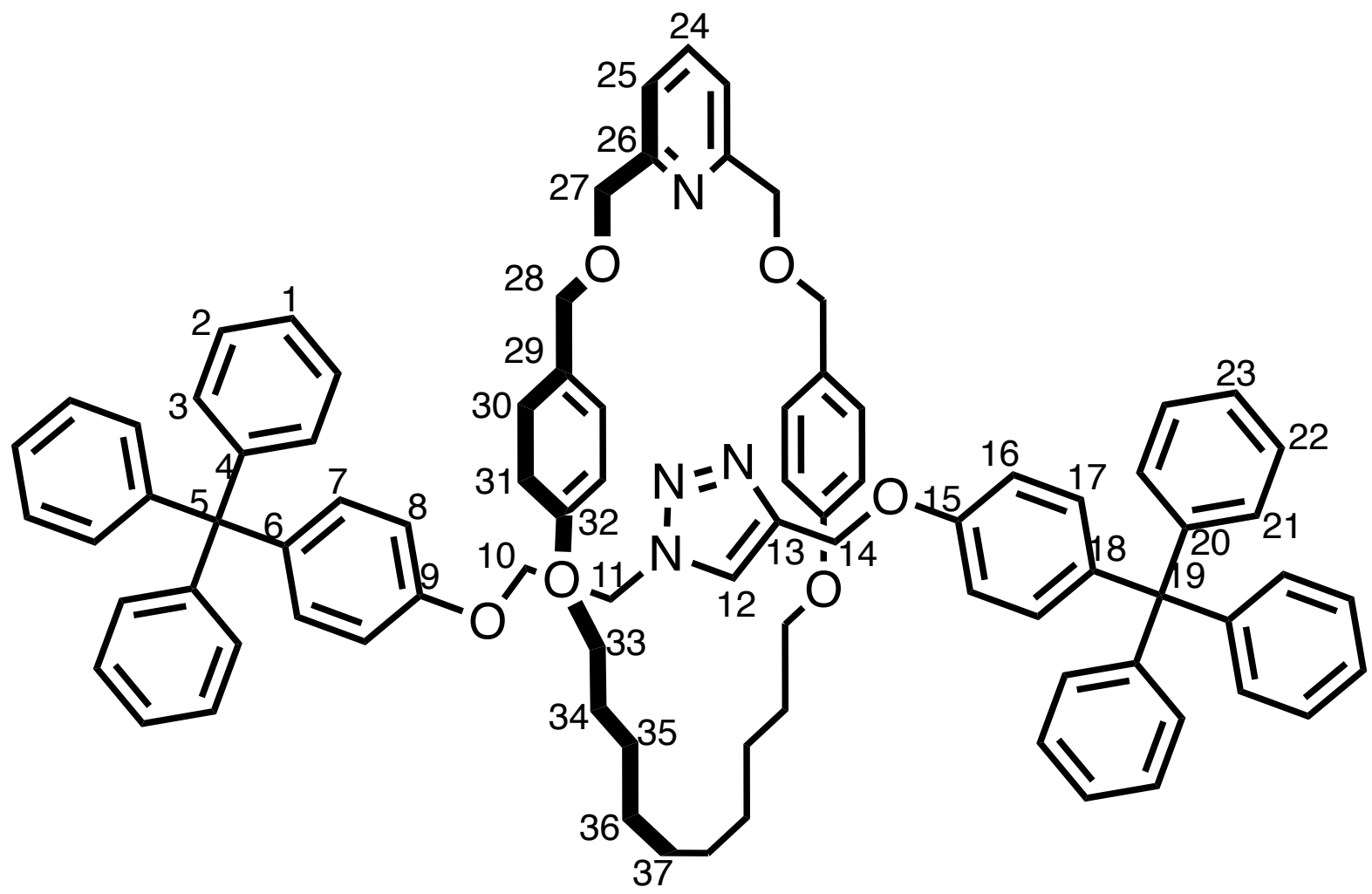

Azide **1** (41.64 mg, 0.10 mmol, 1 eq), alkyne **2** (38.65 mg, 0.10 mmol, 1 eq), macorcycle **3** (49.90 mg, 0.10 mmol, 1 eq), sodium ascorbate (101.84 mg, 0.51 mmol, 5 eq) and $[Cu(I)(MeCN)_4]PF_6$ (38.26 mg, 0.10 mmol, 1 eq) were individually weighed. The powders were transferred into a milling jar with a milling ball (see General Experimental for specifications). The jar was sealed and the reaction was milled in a horizontal oscillating ball-mill grinder (see General Experimental for specifications) at a frequency of 30 Hz for 5 hours. The solid was dissolved in DCM (10 mL) and washed with $HCl_{(aq)}$ (0.1 M, 10 mL). The organic layer was dried ($MgSO_4$) and the solvent was removed *in vacuo*. The mixture was purified by flash column chromatography (SiO_2, 0-100 % EtOAc in Pet. Ether) to afford the product as a pale-yellow amorphous solid (41.3 mg, 0.03 mmol, 33 %).

^{1}H NMR (400 MHz, $CDCl_3$) δ 7.49 (t, *J* = 7.8 Hz, 1H, C24$\underline{H}_1$), 7.45 – 7.39 (m, 1H, C12$\underline{H}_1$), 7.30 – 7.16 (m, 36H, C$\underline{H}Cl_3$, C1$\underline{H}_1$, C2$\underline{H}_1$, C3$\underline{H}_1$, C21$\underline{H}_1$, C22$\underline{H}_1$, C23$\underline{H}_1$ and C25$\underline{H}_1$), 7.09 – 7.03 (d, *J* = 8.7 Hz, 2H, C17$\underline{H}_1$), 7.02 – 6.92 (d, *J* = 8.7 Hz, 6H, C7$\underline{H}_1$ and C31$\underline{H}_1$), 6.74 (d, *J* = 8.4 Hz, 2H, C16$\underline{H}_1$), 6.56 – 6.48 (d, *J* = 8.5 Hz, 4H, C30$\underline{H}_1$), 6.29 (d, *J* = 8.4 Hz, 2H, C8$\underline{H}_1$), 4.88 (s, 2H, C14$\underline{H}_2$), 4.46 (s, 4H, C27$\underline{H}_2$), 4.30 (s, 4H, C28$\underline{H}_2$), 3.90 (s, 2H, C11$\underline{H}_2$), 3.76 (q, *J* = 6.3 Hz, 4H, C33$\underline{H}_2$), 3.51 (s, 2H, C10$\underline{H}_2$), 1.61 (p, *J* = 6.7 Hz, 4H, C34$\underline{H}_2$), 1.28 (d, *J* = 6.6 Hz, 4H, C35$\underline{H}_2$), 1.13 (d, *J* = 12.3 Hz, 8H, C36$\underline{H}_2$ and C37$\underline{H}_2$).

^{13}C NMR (101 MHz, $CDCl_3$) δ 158.8 (C32), 157.6 (C25), 156.5 (C15), 155.7 (C9), 147.2 (C4, C6, C18, C20 and C29), 143.5 (C13), 137.3 (C24), 132.2 (C17), 132.0 (C7), 131.2 (overlapping s, C3 and C21), 130.1 (C31), 127.6 (overlapping s, C2 and C22), 126.0 (C1 and C23), 124.2 (C12), 120.3 (C26), 114.5 (C30), 113.6 (C16), 113.4 (C8), 72.7 (C27), 71.5 (C28), 67.2 (C33), 65.4 (C10), 64.4 (C5 and C19), 61.6 (C14), 48.9 (C11), 29.7 (C35), 28.7 (C34), 25.7 (C36 and C37).

FT-IR (ATR): v_{max}/cm^{-1} 1608, 1508, 1492, 1463, 1445, 1296, 1243, 1184, 1083, 1034, 1015, 906, 827, 726, 700, 646, 628, 594, 585, 524.

HRMS (ES^+): calculated for $C_{86}H_{85}N_4O_6 + H^+$ is 1269.6469, found 1269.6492.

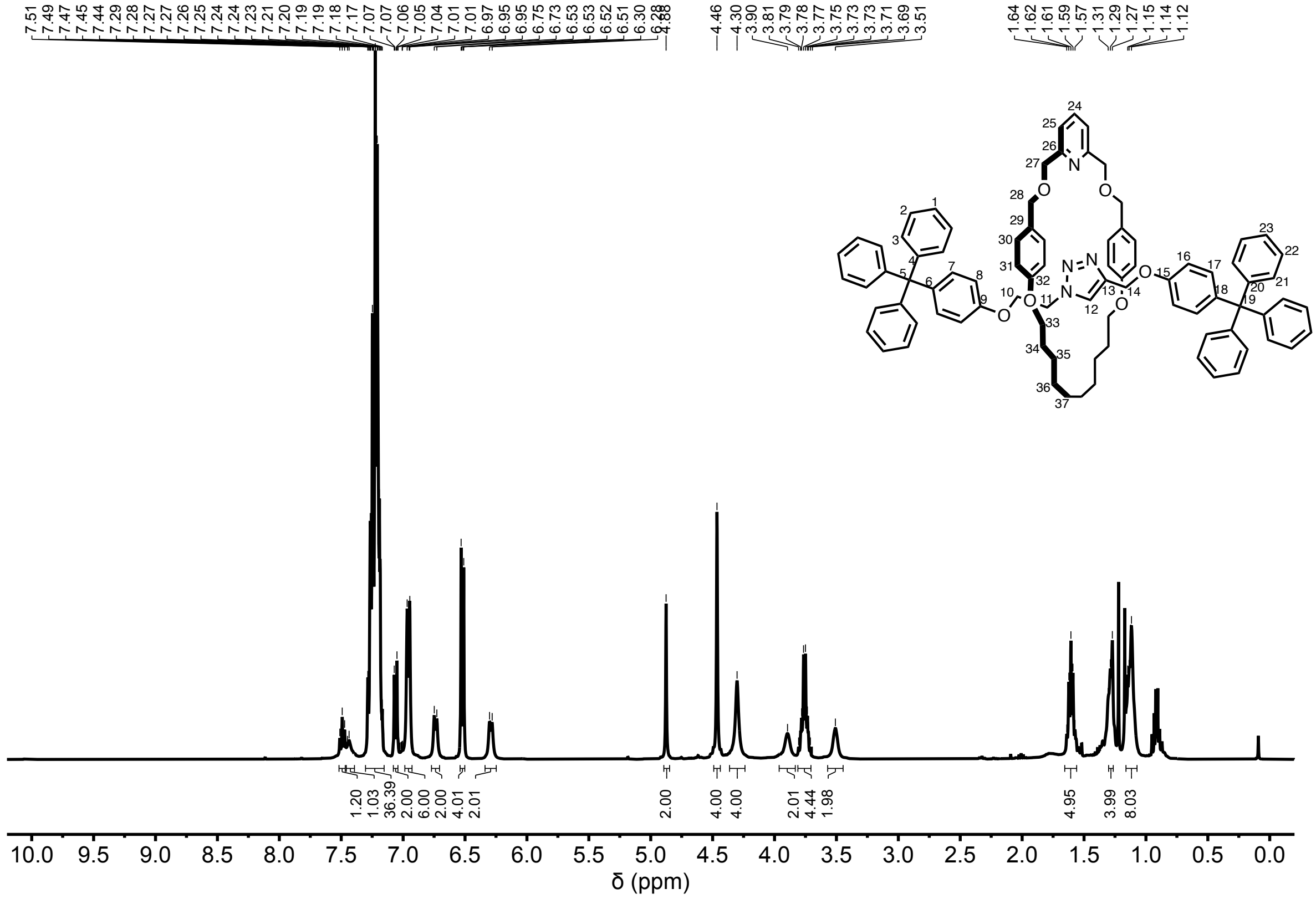

Figure S36 ¹H NMR (400 MHz, $CDCl_3$) spectrum of compound **5.**

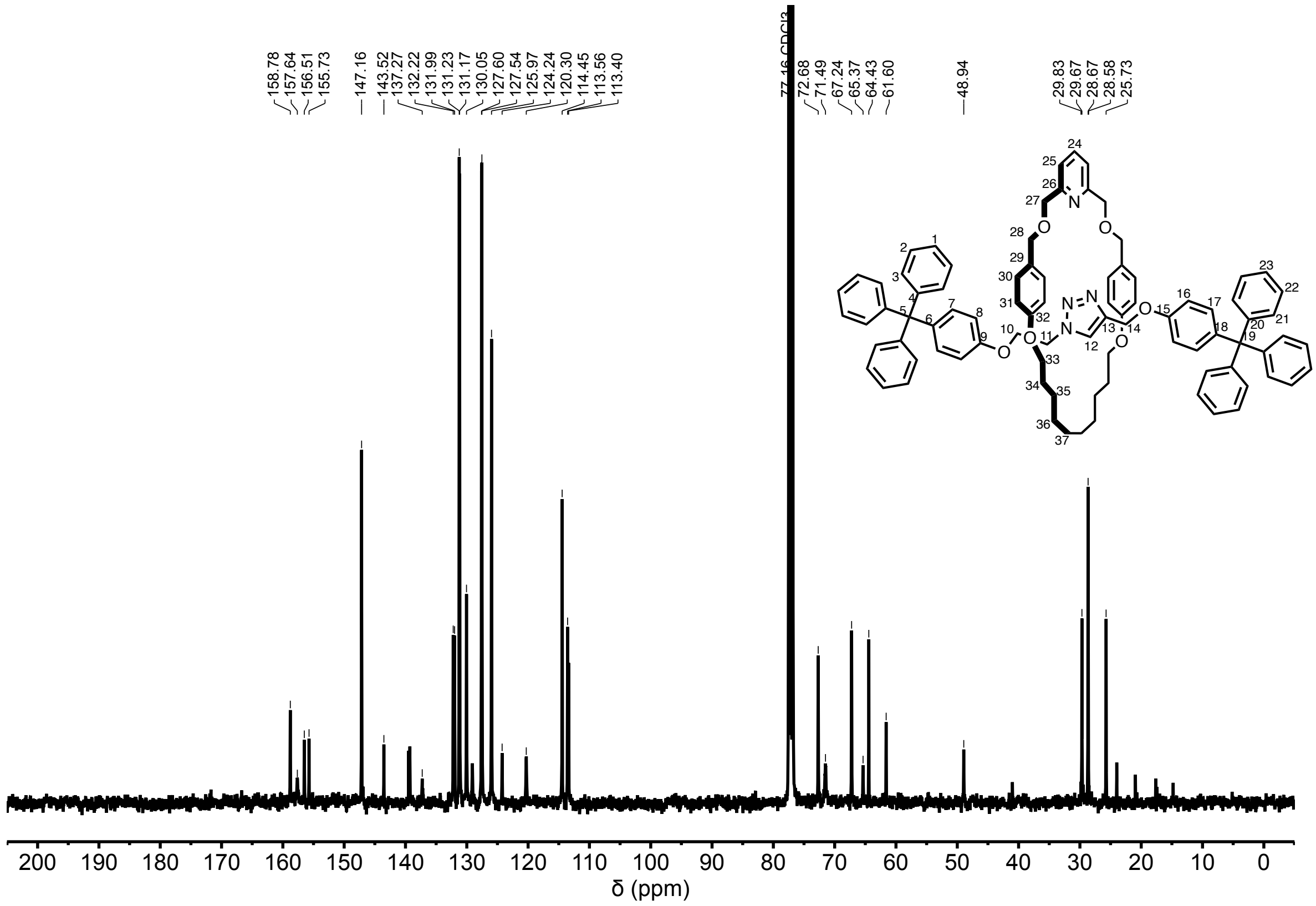

Figure S37 ¹³C NMR (101 MHz, $CDCl_3$) spectrum of compound **5**.

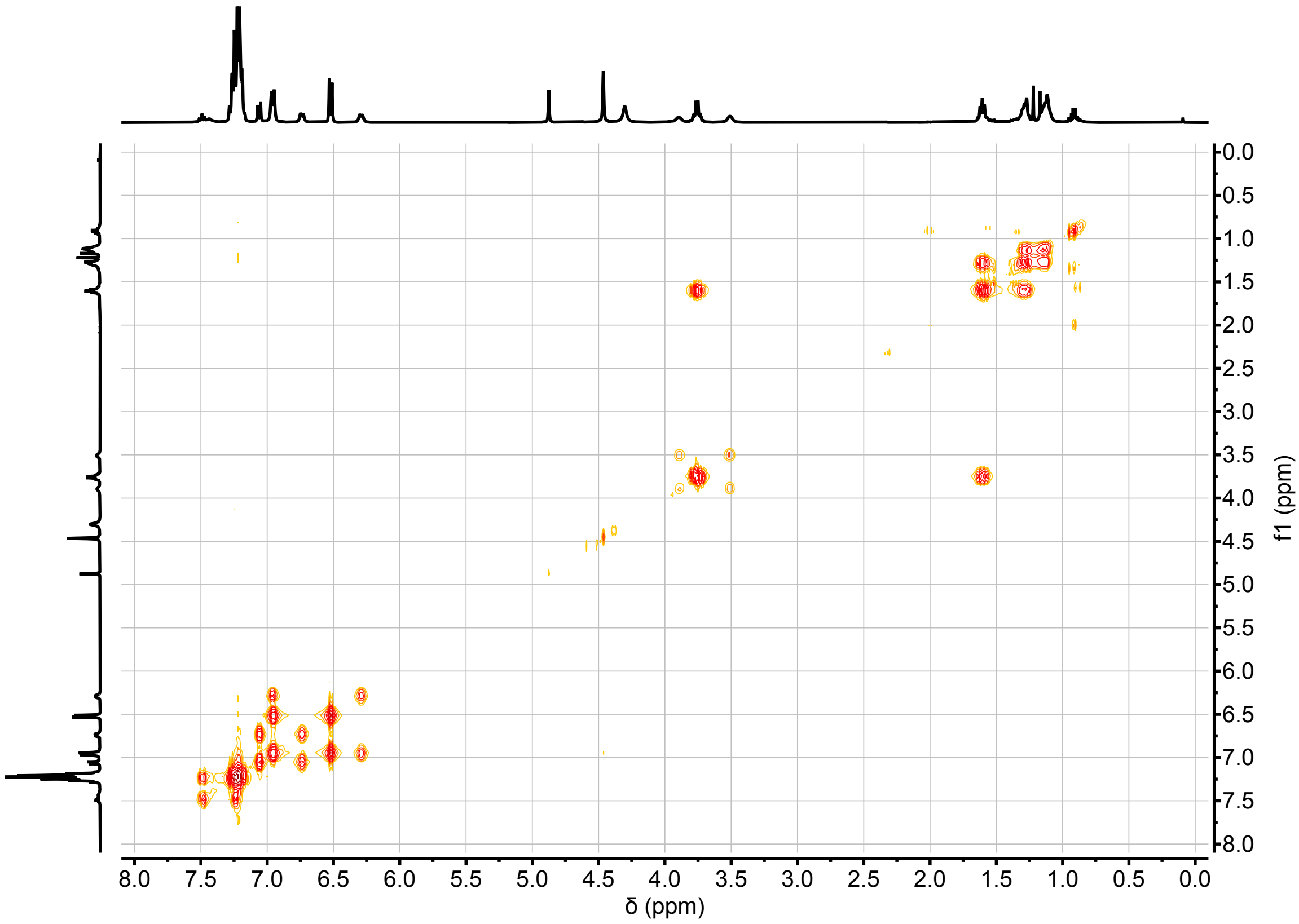

Figure S38 ¹H-¹³C COSY NMR spectrum of compound **5**.

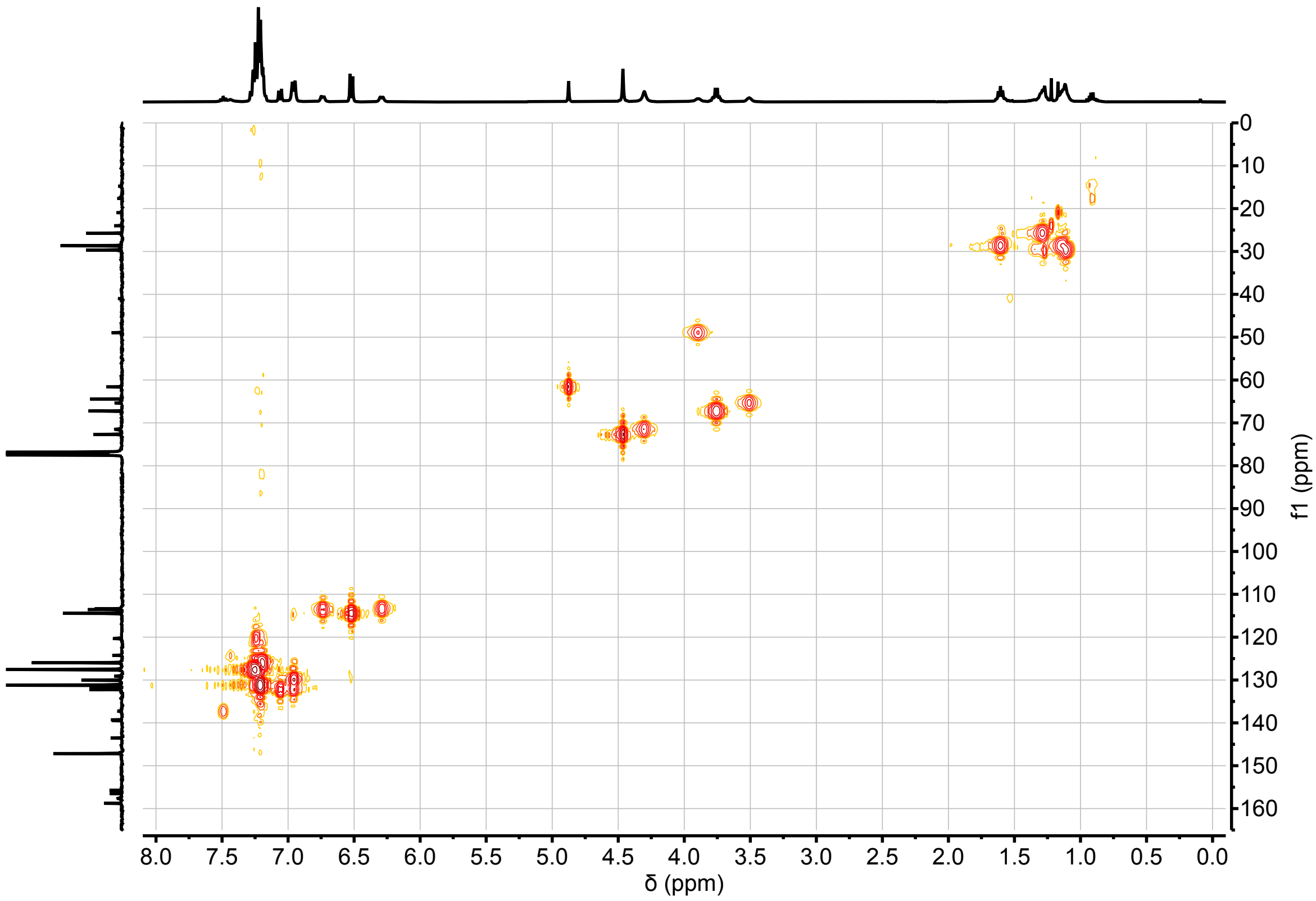

Figure S39 ¹H-¹³C HSQC NMR spectrum of compound **5**.

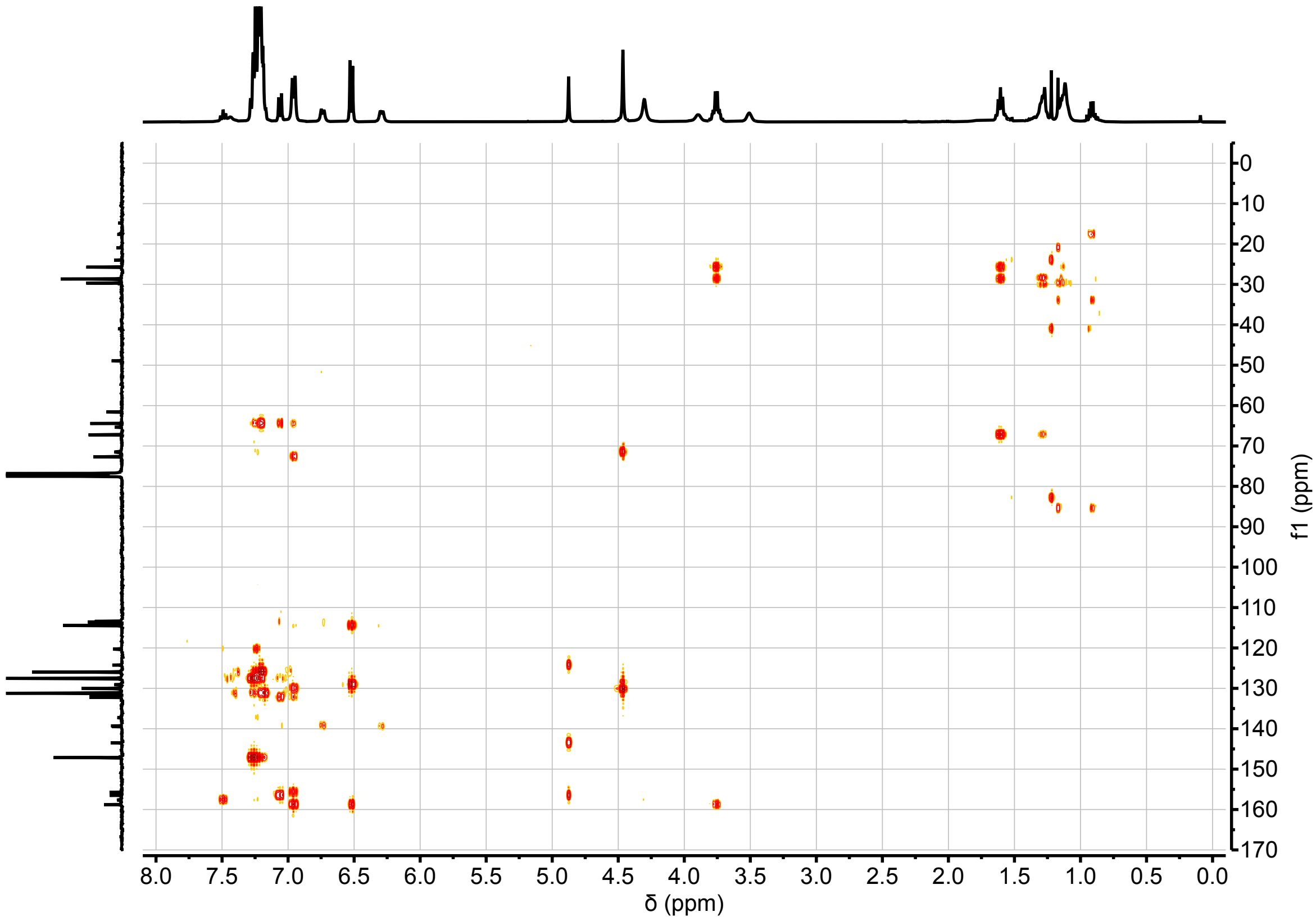

Figure S40 ^{1}H-^{13}C HMBC NMR spectrum of compound **5**.

3 General Procedures for AT-CuAAC by milling

3.1 Optimised reaction procedure

Azide **1** (1 eq), alkyne **2** (1 eq), macorcycle **3** (1 eq), sodium ascorbate (5 eq) and $[Cu(I)MeCN_4]PF_6$ (1 eq) were individually weighed. The powders were transferred into a milling jar with a milling ball (see General Experimental for specifications). The jar was sealed and the reaction was milled in a horizontal oscillating ball-mill grinder (see General Experimental for specifications) at a frequency of 30 Hz for 5 hours. The sample of the solid reaction mixture was removed from the jar and analysed by HPLC.

3.2 Reaction procedure for analytical AT-CuAAC by milling

Solid reactants (see Table S1) were individually weighed and the powders were transferred into a analytical milling jar with a milling ball (see General Experimental for specifications). The powders were mixed with a spatula; the jar was sealed and the reaction was milled in a horizontal oscillating ball-mill grinder (see General Experimental for specifications) at a frequency of 30 Hz for specified duration (see Table S1). Following the reaction, the jar was opened and a sample of the solid reaction mixture was removed from the jar and analysed by HPLC.

Entry	**1**	**2**	**3**	$[Cu(I)(MeCN)_4]PF_6$	Cu(I)I	sodium ascorbate	Total loading / mg	*t* / hours
	/equivalents							
1	1	1	1	1	-	-	33.546	4
1	1	1	1	1	-	-	33.546	6
1[a]	1	1	1	2	-	-	41.163	8
2	1	1	1	1	-	5	45.292	4
2[b]	1	1	1	1	-	5	269.952	5.5
3	1	1	1	-	1	5	34.795	4
4	1	1	5	1	-	5	28.925	4
5[c]	1	1	1	1	-	5	46.107	5
6[d]	1	1	1	-	-	-	36.403	5

Table S1 Variations in the reactant stoichiometry and experimental conditions in the synthesis of [2]-rotaxane **7**. [a] One equivalent of $[Cu(I)(MeCN)_4]PF_6$ was added to the jar described in Entry 1, and the jar was subsequently milled for 2 hours. [b] Reaction was repeated at a larger preparatory scale. [c] Liquid-assisted grinding (LAG) with MeCN (η = 0.1 µL/mg). [d] Reaction was carried out using a copper ball and a copper milling jar in the absence of copper salt.

3.3 Reaction procedure for kinetic study of AT-CuAAC by milling

Reaction procedure for analytical AT-CuAAC by milling (above) using previously described reaction conditions (Table S1 Entry 1). The mixture was milled for 10 minutes at 30 Hz and following the reaction, the jar was opened, a photo of the powder rheology was taken and a sample of the solid reaction mixture was removed from the jar, which was analysed by UPLC.

Following this, the milling jar was resealed, and the mixture was milled for 10 minutes at 30 Hz. This process was repeated for 300 minutes, taking samples at regular time intervals.

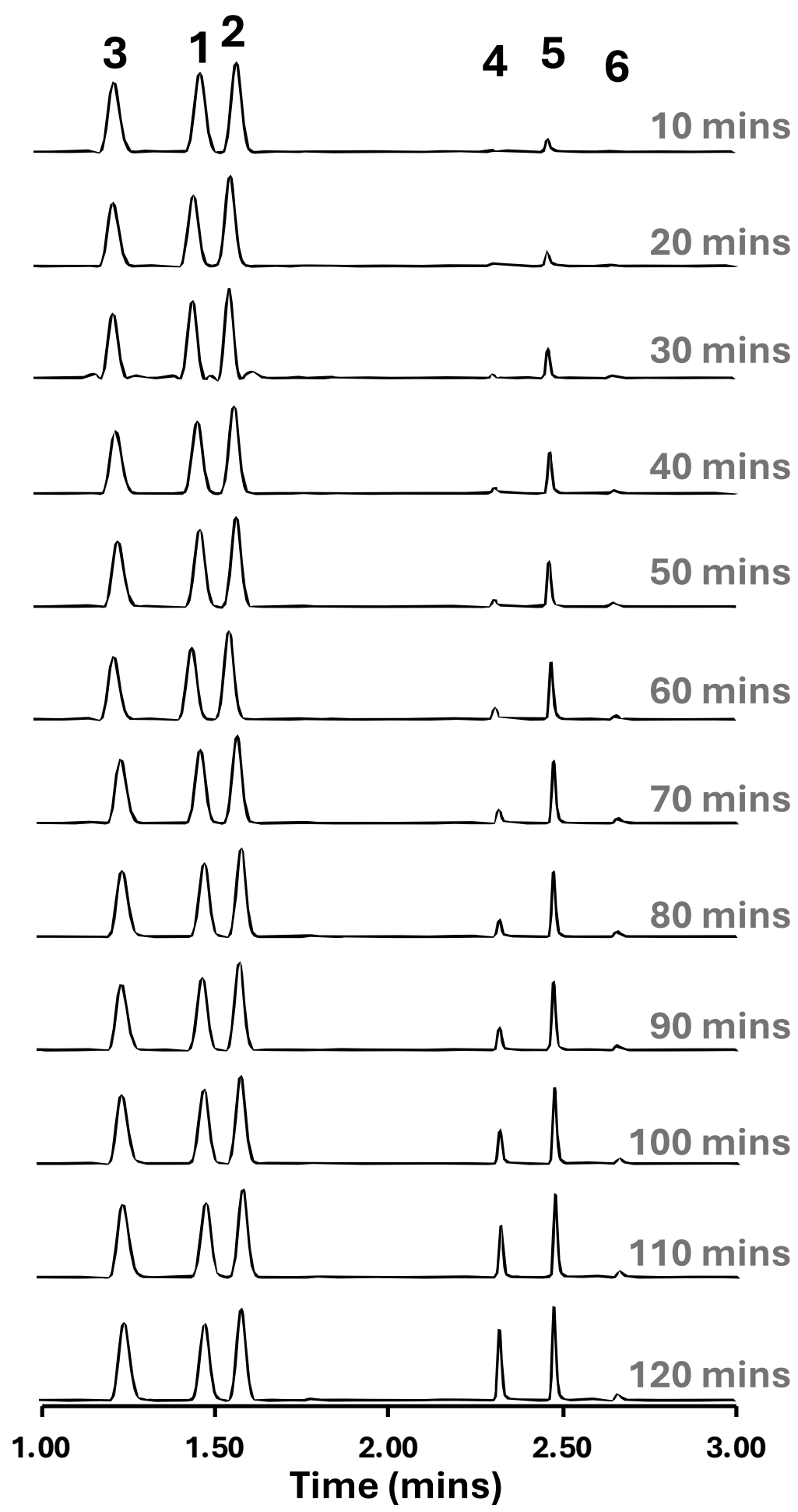

Figure S41 UPLC traces of the crude reaction mixture (Entry 1) of **1** (1 eq), **2** (1 eq), **3** (1 eq), Cu(I)[MeCN]$_4$PF$_6$ (1 eq) taken at regular time intervals. UPLC conditions: C4 column at 40 °C using a gradient of 45–65% of MeCN/formic acid (0.1%) in water/formic acid (0.1%) over 1.5 min, then 65-100% MeCN/formic acid (0.1%) over 0.1 min, and then 100% MeCN/formic acid (0.1%) for 1.5 min. The UV–vis absorbance at 254 nm is plotted.

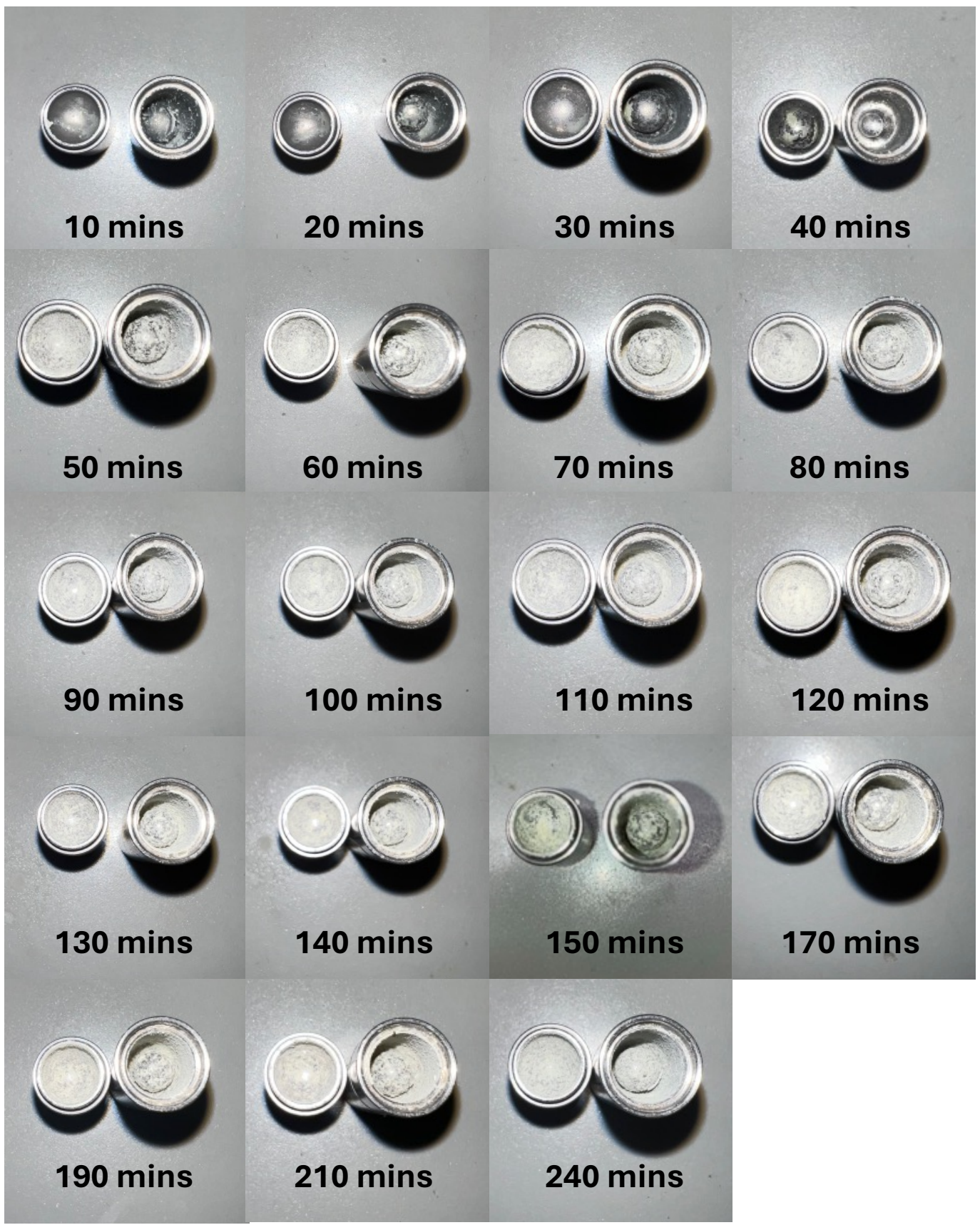

Figure S42 Photographs taken of the milling jar during the kinetic study. A distinct transition in powder morphology was observed between 40 mins and 50 mins.

4 References

(1) Choi, H.; Shirley, H. J.; Hume, P. A.; Brimble, M. A.; Furkert, D. P. Unexpected Direct Synthesis of *N* -Vinyl Amides through Vinyl Azide–Enolate [3+2] Cycloaddition. *Angew Chem Int Ed* **2017**, *56* (26), 7420–7424. https://doi.org/10.1002/anie.201702727.

(2) Aucagne, V.; Hänni, K. D.; Leigh, D. A.; Lusby, P. J.; Walker, D. B. Catalytic "Click" Rotaxanes: A Substoichiometric Metal-Template Pathway to Mechanically Interlocked Architectures. *J. Am. Chem. Soc.* **2006**, *128* (7), 2186–2187. https://doi.org/10.1021/ja056903f.

(3) Fuller, A.-M. L.; Leigh, D. A.; Lusby, P. J.; Slawin, A. M. Z.; Walker, D. B. Selecting Topology and Connectivity through Metal-Directed Macrocyclization Reactions: A Square Planar Palladium [2]Catenate and Two Noninterlocked Isomers. *J. Am. Chem. Soc.* **2005**, *127* (36), 12612–12619. https://doi.org/10.1021/ja053005a.

(4) Yasukouchi, T.; Kanematsu, K. The Total Synthesis of (±)--Trikentrin B Allene Intramolecular Cycloaddition. *Tetrahedron Letters* **1989**, *30* (47), 6559–6562. https://doi.org/10.1016/S0040-4039(01)89021-3.

(5) Druais, V.; Hall, M. J.; Corsi, C.; Wendeborn, S. V.; Meyer, C.; Cossy, J. A Convergent Approach toward the C1−C11 Subunit of Phoslactomycins and Formal Synthesis of Phoslactomycin B. *Org. Lett.* **2009**, *11* (4), 935–938. https://doi.org/10.1021/ol8029142.
